# Deciphering cell type-specific causal genetic effects on brain imaging-derived phenotypes and disorders with single-cell Mendelian randomization

**DOI:** 10.1371/journal.pcbi.1014422

**Published:** 2026-06-17

**Authors:** Anyi Yang, Xingzhong Zhao, Xing-Ming Zhao, Yucheng T. Yang

**Affiliations:** 1 Department of Neurology, Zhongshan Hospital and Institute of Science and Technology for Brain-Inspired Intelligence, Fudan University, Shanghai, China; 2 College of Biomedical Engineering, Fudan University, Shanghai, China; 3 Department of Cardiovascular Medicine, RuiJin Hospital Lu Wan Branch, Shanghai Jiaotong University School of Medicine, Shanghai, China; 4 Huzhou Central Hospital, Affiliated Central Hospital Huzhou University, Huzhou, Zhejiang, China; 5 State Key Laboratory of Brain Function and Disorders, Institutes of Brain Science, Fudan University, Shanghai, China; 6 MOE Frontiers Center for Brain Science, Fudan University, Shanghai, China; University of Wisconsin Madison, UNITED STATES OF AMERICA

## Abstract

Reconstructing causality routes from genetic effects to complex phenotypes in particular cell types is crucial for understanding biological mechanisms underlying the brain-associated phenotypes including imaging-derived phenotypes (IDPs), and brain disorders and behaviors (DBs). Here, we develop a single-cell Mendelian randomization framework to infer cell type-specific causal relationships between gene expression and diverse brain-associated complex phenotypes by integrating single-cell expression quantitative trait loci (*cis*-eQTLs) and genome-wide association study findings. We identifiy a set of 254 and 217 *cis*-eQTL target genes (eGenes) that may have causal effects on 112 IDPs and 26 DBs in eight cell types, respectively. These causal eGenes exhibit strong cell type specificity and varied pleiotropy among different types of brain-associated phenotypes. Further integrative analysis reveals putative causality routes among cell type-specific causal eGenes and brain-associated complex phenotypes. Finally, we characterize the spatiotemporal expression patterns of these causal eGenes, and highlight the coordinated associations of the brain-associated phenotypes based on the expression of their causal eGenes. Overall, our study presents a large-scale analysis of the genetic effects of brain structures, disorders and behaviors, providing a catalog of cell type-specific causal eGenes.

## Author summary

Our understanding of how genes influence brain phenotypes at the cell type level remains limited. Although genome-wide association studies (GWASs) have identified numerous genetic variants associated with brain structure and disease, the majority reside in non-coding regions and do not directly implicate specific genes or cell types. Here, we integrate GWAS summary statistics with *cis*-expression quantitative trait loci (*cis*-eQTL) data from eight brain cell types to identify genes whose expression levels may causally influence 149 brain phenotypes spanning structural features, disorders, and behaviors. Using Mendelian randomization, we detect hundreds of such genes with cell type–specific effects. These eGene–phenotype associations are highly cell type–specific, and many putatively causal genes are shared across phenotype categories. Integrating these findings, we reconstruct putative routes linking cell type–specific causal genes to brain structure and disease. We further reveal spatiotemporal expression patterns of these genes across brain phenotypes and developmental stages. Together, these findings provide a cell type–specific portrait of the genetic contributions to brain phenotypes.

## Introduction

Recent large-scale genome-wide association studies (GWASs) have identified thousands of genetic variants associated with brain-related complex phenotypes [1,2]. However, interpreting the regulatory roles of these loci remains challenging, as the majority reside in non-coding regions and do not directly implicate specific genes [3]. Expression quantitative trait loci (*cis*-eQTLs), which link genetic variants to the expression levels of target genes, provide a powerful means of bridging this gap [4,5]. By integrating *cis*-eQTL data with GWAS findings, Mendelian randomization (MR)—a framework for genetic causal inference—can pinpoint genes whose expression causally influences complex neurological, psychological, and behavioral phenotypes [6–9].

Previous MR studies have largely relied on bulk-tissue *cis*-eQTLs to identify causal genes for complex phenotypes [7,10,11]. Yet cell type–level *cis*-eQTLs exhibit larger effect sizes and target more evolutionarily constrained genes than their tissue-level counterparts [12]. suggesting that cellular resolution is critical for accurate causal inference. Recent single-cell eQTL atlases—including OneK1K [13], Bryois et al [12], and MetaBrain [14]—have revealed substantial cell type–specific effects of genetic variants on gene expression regulation. Moreover, cell type–level analyses have uncovered disorder-specific genetic controls that remain invisible to bulk tissue approaches [12,13,15–17]. For instance, most genetic variants associated with Alzheimer’s disease (AD) risk appear to act through *cis*-eQTLs in microglia, whereas schizophrenia (SCZ) exhibits a more polygenic architecture with signals spanning multiple cell types [12]. These observations indicate that cellular-level regulation is more closely coupled to disease pathogenesis than bulk-level regulation. Nevertheless, how genetic variants contribute to causal gene–phenotype relationships at the cell type level remains poorly understood, motivating the cell type–specific causal inference pursued in this study.

An additional layer of complexity lies in the genetic and cellular links between brain imaging–derived phenotypes (IDPs) and brain disorders and behaviors (DBs). Extensive genetic correlations between imaging features and complex phenotypes have been documented [18–23], and recent large-scale GWASs have identified overlapping loci and genes associated with both brain imaging phenotypes and diverse disorders [19,22,24,25]. Notably, causal eGenes shared across psychiatric, neurodegenerative, and structural brain phenotypes often exhibit cell type–specific expression patterns. For example, *AKT3*—which is implicated in cortical surface area, Parkinson’s disease (PD), and major depressive disorder—shows astrocyte-specific expression [18], whereas DIP2B, shared between SCZ and brain stem volume, is enriched in excitatory neurons [18]. Consistent with these gene-level observations, the genetic heritability of both IDPs and DBs is significantly enriched in common cell types: oligodendrocytes for white matter microstructure and depression [22,26], and excitatory neurons for total surface area [27] and schizophrenia [28]. Collectively, these findings suggest that shared cellular-level genetic architectures may mediate the relationships between brain structural phenotypes and brain disorders. Characterizing these putative pathways could therefore advance the development of mechanistically informed therapeutic targets.

In this study, we systematically inferred cell type–specific causal relationships between gene expression and diverse brain-associated complex phenotypes. We developed a single-cell MR framework that integrates a recently published *cis*-eQTL atlas from eight brain cell types with large-scale GWAS findings for 26 DBs (8 behavioral-cognitive phenotypes, 10 psychiatric disorders, and 8 neurological disorders) and 123 IDPs (101 brain regional volumes and 22 white matter tracts). For the identified causal *cis*-eQTL target genes (eGenes), we examined their cell type specificity and pleiotropic patterns across different phenotype categories. We further reconstructed putative causality routes linking cell type–specific causal eGenes to brain-associated complex phenotypes, and characterized the spatiotemporal expression dynamics of these genes using external single-cell data. Our findings illuminate how cell type–specific gene expression shapes brain structure, disorders, and behaviors through genetically driven regulatory mechanisms, offering insights into both healthy and pathological brain states.

## Results

### Overview of the study

**Fig 1:**
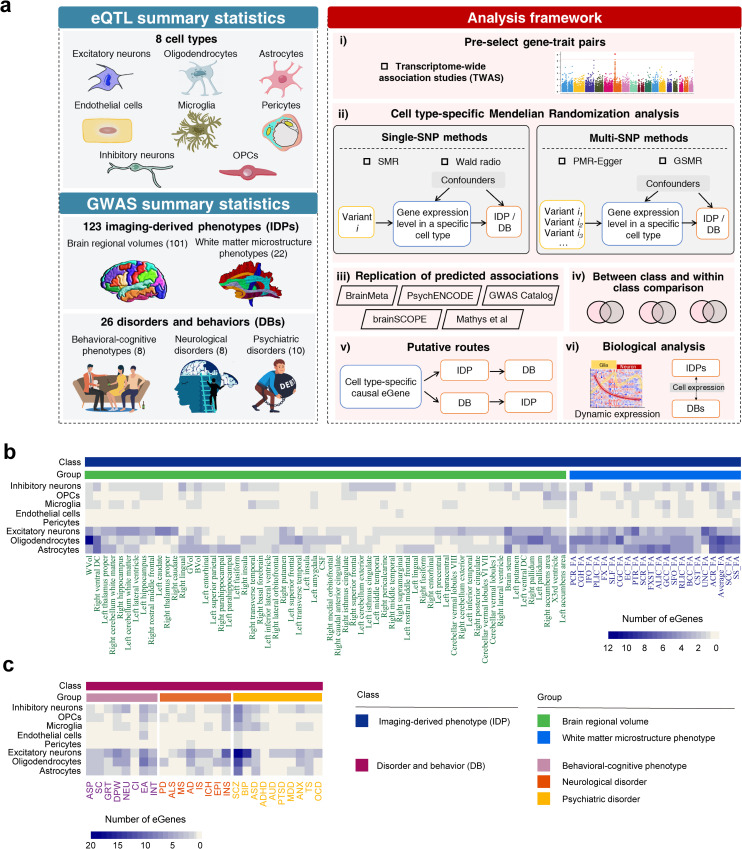
Study workflow and cell type–specific causal eGene counts across brain-associated phenotypes. **(a)** Workflow. The *cis*-eQTL data from eight brain cell types were integrated with GWAS summary statistics for 123 IDPs and 26 DBs. TWAS pre-screening was followed by cell type–specific Mendelian randomization to infer putatively causal eGenes for 149 phenotypes, classified into two classes (IDPs and DBs) and five groups (psychiatric disorders, neurological disorders, behavioral–cognitive phenotypes, brain regional volumes, and white matter tracts). Downstream analyses included shared eGene characterization, single-cell expression profiling, and causal route reconstruction. Brain region schematics adapted from the Desikan-Killiany Atlas [34]; white matter tract schematics from the Atlas Track [35]. **(b)** Heatmap of cell type–specific causal eGene counts across IDPs (only IDPs with >3 eGenes are shown). **(c)** Heatmap of cell type–specific causal eGene counts across DBs.

Dysregulation of gene expression in specific brain cell types is a hallmark of psychiatric and neurological disorders [12,13,15,16,29]. To systematically infer cell type–specific causal relationships between gene expression and brain-associated complex phenotypes, we performed two-sample Mendelian randomization (MR) by integrating GWAS summary statistics for 26 disorders and behaviors (DBs) and 123 imaging-derived phenotypes (IDPs) with a recently published cell type–specific *cis*-eQTL atlas encompassing eight brain cell types [12] (**S1****–****S3 Tables**). The eight cell types were astrocytes, endothelial cells, excitatory neurons, inhibitory neurons, microglia, oligodendrocytes, oligodendrocyte precursor cells (OPCs), and pericytes. The 26 DBs comprised three groups: 8 behavioral-cognitive phenotypes, 8 neurological disorders, and 10 psychiatric disorders (mean GWAS sample size: 284,168; **S1 Table**). The 123 IDPs comprised two groups: 101 brain regional volumes and 22 white matter microstructure phenotypes measured by mean fractional anisotropy (mean GWAS sample size: 22,072; **S2 Table**). The overall study design is depicted in **Fig 1a**.

Given evidence of bidirectional causal effects between brain disorders and imaging phenotypes [20,30], we first screened for potential eGene–phenotype associations using transcriptome-wide association study (TWAS) [31], retaining 43,494 eGene–DB pairs and 284,927 eGene–IDP pairs at nominal significance (*P* < 0.05) for subsequent MR analysis. To ensure instrument validity, we selected as instrumental variables (IVs) only those single nucleotide polymorphisms (SNPs) that achieved genome-wide significance (*P* < 5 × 10^-8^) in the *cis*-eQTL data. We manually verified the sample descriptions of all datasets and confirmed the absence of participant overlap between the *cis*-eQTL catalog and the GWAS cohorts.

To enhance the robustness of causal inference, we applied four complementary MR methods: SMR [6], Wald ratio [32], PMR-Egger [8] and GSMR [33]. These methods differ in their IV selection strategies (single-SNP versus multi-SNP; independent versus correlated SNPs), tolerance for horizontal pleiotropy, and analytical assumptions (**S1 Fig** and **Methods**), thereby providing convergent evidence for each inferred association. We then assessed the reliability of the predicted cell type–specific causal eGenes using external functional genomic datasets from the human brain. Next, we characterized the pleiotropic landscape of these causal eGenes across phenotype classes and groups, reconstructed putative causality routes linking cell type–specific eGenes to DBs and IDPs, and investigated the spatiotemporal expression dynamics of these genes using independent single-cell data.

### Putative causal effects of eGenes on IDPs and DBs

We identified 254 eGenes with putative causal effects on 112 IDPs across eight cell types (FDR < 0.05, corrected across all eGene–cell type–IDP tests), yielding 760 significant eGene–cell type–IDP combinations (**Fig 1b** and **S4 Table**). Among these, 184 and 112 eGenes were implicated in 90 brain regional volumes and 22 white matter microstructure phenotypes, respectively. To assess reproducibility, we replicated the MR analyses using cortical eQTLs from BrainMeta [36] and cell type–specific eQTLs from brainSCOPE [37]. Of the eGene–IDP pairs, 64.9% (430/663) and 49.2% (326/663) were replicated in BrainMeta at nominal and FDR < 0.05 levels, respectively (both *P* < 10^-16^, hypergeometric test; **S5 Table**and **S2 Fig**). For eGene–cell type–IDP triples, 41.9% (315/751) and 33.7% (253/751) were replicated in brainSCOPE at nominal and FDR < 0.05 levels, respectively (both *P* < 10^-16^, hypergeometric test; **S5 Table** and **S3 Fig**).

These associations were strongly cell type–specific: 91.4% (605/662) of eGene–IDP pairs were significant in only one cell type, consistent with the notion that brain structure is shaped by distinct cellular programs [38–40]. This specificity persisted after accounting for eQTL availability—among genes with valid instrumental *cis*-eQTLs in two or more cell types, 80.6% (237/294) remained significant in exactly one cell type (**S4 Fig**). White matter microstructure phenotypes harbored more causal eGenes in oligodendrocytes and astrocytes than brain regional volumes did (**Fig 1b**), mirroring prior evidence that white matter heritability is enriched in glial cells [22]. The strongest enrichment occurred between white matter volume and oligodendrocytes (12 eGenes), eight of which (*ANKRD44*, *ZCWPW1*, *SLC16A8*, *PILRB*, *RUNX2*, *LARP6*, *C7orf61* and *SUPT3H*) have been linked to neuroimaging phenotypes in the GeneCards database [41]. Collectively, these results nominate candidate cell type–specific regulators of brain structural phenotypes. Nevertheless, because the *cis*-eQTL data were derived from the prefrontal cortex, temporal cortex, and deep white matter—not all anatomical regions represented by the imaging outcomes—these findings should not be interpreted as pinpointing the precise brain region where each regulatory effect operates [42–44].

Turning to DBs, we identified 217 eGenes with putative causal effects on 26 DBs across eight cell types (FDR < 0.05, corrected across all eGene–cell type–DB tests), comprising 298 eGene–cell type–DB combinations (**Fig 1c** and **S6 Table**). Of these, 109, 61, and 85 eGenes were associated with 10 psychiatric disorders, 8 neurological disorders, and 8 behavioral-cognitive phenotypes, respectively. We validated these findings using three independent resources: BrainMeta cortical eQTLs [36], PsychENCODE differential expression data [29], and the GWAS Catalog [1]. Replication rates were 35.7% (87/244) and 23.0% (56/244) in BrainMeta at nominal and FDR < 0.05 levels, respectively; 29.5% (23/78) in PsychENCODE; and 23.8% (57/240) in the GWAS Catalog (all *P* < 10^-4^, hypergeometric test; **S7 Table** and **S2 Fig**). For eGene–cell type–DB triples, replication in brainSCOPE [37] was 41.9% (315/751) nominally and 33.7% (253/751) at FDR < 0.05 (both with *P* < 10^-16^, hypergeometric test; **S7 Table** and **S3 Fig**). Notably, 12 of the 14 AD-associated cell type–specific causal eGenes identified here were corroborated by Mathys et al. [45].

As with IDPs, the eGene–DB associations were highly cell type–specific: 82.1% (197/240) of pairs were restricted to a single cell type, and among genes with valid instruments in multiple cell types, 69.2% (92/133) were significant in exactly one (**S4 Fig**). These patterns support the view that most genes influencing brain disorders and behaviors exert their effects through discrete cell types [12,46]. Excitatory neurons harbored the largest number of causal eGenes across nearly all phenotype groups, notably in SCZ, bipolar disorder (BIP), and insomnia (INS). Inhibitory neurons contained fewer causal eGenes overall but remained prominently involved in SCZ. The SCZ–excitatory neuron pair yielded the greatest number of causal eGenes (20), four of which (*ANKRD27*, *LIN28B*, *UROS* and *CCHCR1*) have been linked to SCZ in the GeneCards database [41]. Moreover, five of the six eGenes predicted to influence AD in excitatory neurons—including *MSH3* [47], *ICA1L* [48], *RGS14* [49], *C17orf97* [50] and *ZSCAN31* [51]—have established roles in AD and other neurodegenerative disorders, suggesting their convergent contributions to neurodegenerative pathogenesis.

### Pervasive phenotype pleiotropy of causal eGenes

**Fig 2:**
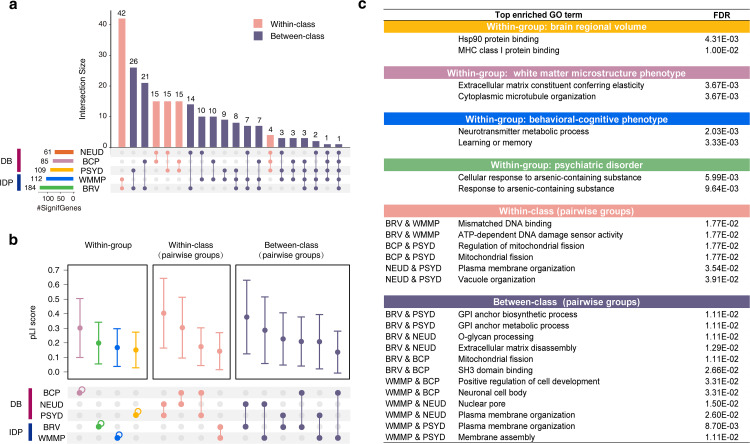
Causal eGenes shared across IDPs and DBs. **(a)** Upset plot of cell type–specific causal eGenes shared across phenotype classes and groups. **(b)** pLI scores of pairwise shared causal eGenes. **(c)** Top two enriched gene sets for pairwise shared eGenes. Within-group: shared by ≥2 phenotypes within a group. Within-class: shared across groups of the same class. Between-class: shared between the DB and IDP classes. BRV: Brain regional volume; WMMP: White matter microstructure phenotype; BCP: Behavioral-cognitive phenotype; NEUD: Neurological disorder; PSYD: Psychiatric disorder.

To characterize the pleiotropic landscape of the identified causal eGenes, we systematically compared their sharing patterns across phenotype classes (IDPs versus DBs) and phenotype groups (brain regional volumes and white matter microstructure phenotypes within IDPs; behavioral-cognitive phenotypes, neurological disorders, and psychiatric disorders within DBs). Significant eGene overlap was observed between every pair of phenotype groups (all with *P* < 10^-10^, hypergeometric test; **S5 Fig**). Within the IDP class, the strongest overlap occurred between brain regional volumes and white matter microstructure phenotypes (42 shared eGenes, 37.5%), with the inferior fronto-occipital fasciculus (IFO) contributing the largest fraction of white matter phenotypes to this overlap (22.81%; **Figs 2a** and **S6**). Notably, this pleiotropy was not fully explained by genetic correlations among IDPs. Across 7,503 IDP pairs, the absolute genetic correlation was only weakly associated with eGene overlap (Spearman’s rank correlation coefficient *r* = 0.092, *P* = 1.95 × 10^-15^; **S7b Fig**), and the association remained weak for between-group pairs (Spearman’s rank correlation coefficient *r* = 0.073, *P* = 6.10 × 10^-4^). Indeed, even the most strongly correlated between-group pair (PLIC_FA and right lateral ventricle, *r*_*g*_ = 0.49) shared no causal eGenes. Within the DB class, four eGenes exhibited pleiotropic effects across all three DB groups—*DDHD2* and *LIN28B* in excitatory neurons, and *MAP2K5* and *XKR6* in inhibitory neurons (**S8 Table**)—all of which have been implicated in multiple psychiatric disorders [52–55].

Between-class pleiotropy was similarly extensive. The brain regional volume group overlapped most strongly with the behavioral-cognitive phenotype group (21 shared eGenes, 24.7%), driven primarily by drinks per week (DPW) (10.45%; **Figs 2a** and **S6**); and with the psychiatric disorder group (26 shared eGenes, 23.85%), driven primarily by attention-deficit/hyperactivity disorder (ADHD) (15.38%; **Figs 2a** and **S6**). More broadly, 17 eGenes were shared between two IDP groups and one DB group, nine between one IDP group and two DB groups, four across four groups (*XKR6* in inhibitory neurons, *MAPT* in astrocytes, *ZSCAN31* in astrocytes, and *MSH3* in excitatory neurons), and one eGene—*XKR6* in inhibitory neurons—was shared across all five phenotype groups (**Fig 2a** and **S8 Table**). Collectively, these findings indicate that cell type–specific genetic susceptibilities are shared across diverse categories of brain-associated phenotypes.

We next examined the evolutionary constraint and functional profiles of shared causal eGenes using probability of loss-of-function intolerance (pLI) scores from ExAc [56] and gene-set enrichment analysis [57]. Among genes shared within individual phenotype groups, those in the behavioral-cognitive phenotype group exhibited the highest constraint (*μ* = 0.302, *σ*^*2*^ = 0.203), followed by the brain regional volume group (*μ* = 0.20, *σ*^*2*^ = 0.14; **Fig 2b**, left panel). Functionally, the behavioral-cognitive shared eGenes were enriched in pathways governing memory, cognition, and neurotransmission (**Fig 2c** and **S9 Table**), consistent with their established roles in learning and cognitive performance [58,59]. By contrast, brain regional volume–shared eGenes were enriched in protein-binding functions, notably Hsp90 and MHC class I protein binding (**Fig 2c** and **S9 Table**), which regulate synapse formation and [60,61].

For eGenes shared within the same phenotype class but across groups, the neurological disorder–psychiatric disorder overlap exhibited the highest pLI scores (*μ* = 0.409, *σ*^*2*^ = 0.235), whereas the brain regional volume–white matter microstructure overlap showed the lowest (*μ* = 0.153, *σ*^*2*^ = 0.125; **Fig 2b**, middle panel). Concordantly, the neuropsychiatric shared eGenes were enriched in cell maintenance and signaling pathways such as plasma membrane organization (**Fig 2c** and **S9 Table**), which have been linked to the etiology of neuropsychiatric disorders [62,63].

Finally, for between-class shared eGenes, those bridging brain regional volumes and DB groups (behavioral-cognitive, neurological, and psychiatric) exhibited higher pLI scores than those bridging white matter microstructure and DB groups (**Fig 2b**, right panel). This suggests that volumetric measures may capture more fundamental and evolutionarily conserved genetic determinants of brain disorders than microstructural measures—a pattern consistent with evidence that microstructural changes typically precede volumetric changes during disease progression [64,65], and that the genetic drivers of such early microstructural alterations are correspondingly less constrained. Notably, the brain regional volume–neurological disorder shared eGenes, which displayed the highest between-class pLI scores, were enriched in four pathways related to extracellular matrix dynamics (**Fig 2c** and **S9 Table**). Given the central role of the extracellular matrix in brain volume regulation, neural development, and disease pathophysiology [66–68], these results underscore the importance of extracellular matrix biology in the shared genetic architecture of brain structure and neurological disorders.

### Shared cell type specificity of causal eGenes between different phenotype classes

**Fig 3:**
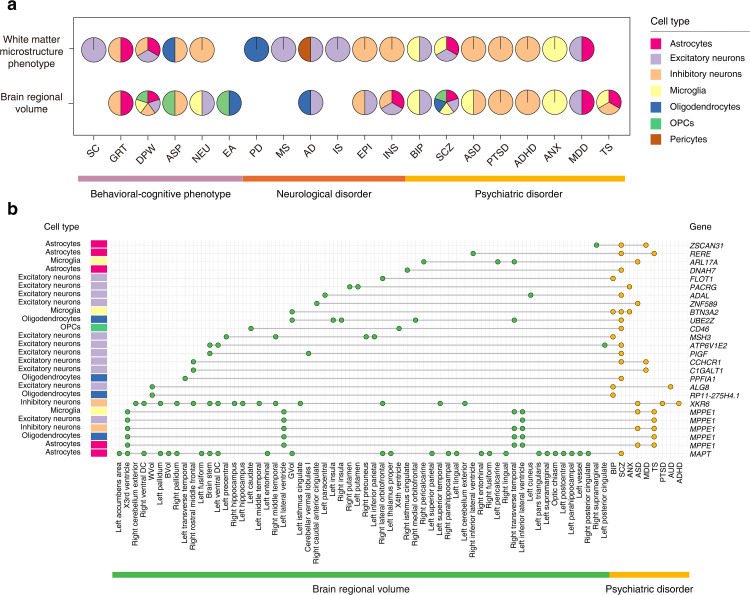
Shared cell type–specific eGenes between IDPs and DBs. **(a)** Brain cell types showing significant sharing of causal eGenes between IDP groups and individual DB phenotypes. Cell types were tested by hypergeometric test within each cell type and selected at FDR < 0.05. **(b)** Causal eGenes shared between brain regional volumes and psychiatric disorders.

Having established the pervasive sharing of causal eGenes across phenotype classes, we next examined the specific cell types that mediate these shared associations between IDPs and DBs. The cell type landscape differed markedly across DB–IDP pairs (**Fig 3a**). SCZ and drinks per week (DPW) each shared causal eGenes with brain regional volumes across a broad spectrum of cell types—including astrocytes, excitatory neurons, microglia, oligodendrocytes, and OPCs for SCZ, and astrocytes, excitatory neurons, microglia, inhibitory neurons, and OPCs for DPW—consistent with their polygenic architecture [69,70]. By contrast, neurodevelopmental disorders such as autism spectrum disorder (ASD), post-traumatic stress disorder (PTSD), and ADHD showed restricted sharing confined to inhibitory neurons (**Fig 3a**), supporting the hypothesis that disrupted inhibitory neurotransmission underlies both the structural and functional abnormalities seen in these conditions [71–73]. Notably, several risky behaviors—general risk tolerance (GRT), automobile speeding propensity (ASP), and DPW—also shared inhibitory neuron eGenes with both brain regional volumes and white matter microstructure phenotypes (**Fig 3a**). Furthermore, OPC-specific sharing with brain regional volumes was observed exclusively for SCZ and the behavioral-cognitive phenotypes DPW, ASP, and educational attainment (**Fig 3a**), in line with evidence that OPC gene expression shapes neural circuit development and cognitive outcomes [74,75].

These patterns point to a common cell type–specific genetic susceptibility linking brain structure to disorders and behaviors. Among psychiatric disorders, the *MAPT* gene in astrocytes emerged as a prominent shared signal, implicated in SCZ alongside 26 brain regional volumes and 15 white matter microstructure phenotypes (**Figs 3b** and **S8**). Increased astrocytic *MAPT* expression was associated with elevated SCZ risk, reduced mean fractional anisotropy in 14 white matter tracts, and decreased volume in 17 brain regions. Located at 17q21, *MAPT* is pivotal for human brain size and patterning, with established links to intracranial volume, white matter microstructure, and cortical architecture [24]. *MAPT* mutations drive brain atrophy [76], and *MAPT* dysregulation has been tied to SCZ [77], while astrocytic dysfunction itself is increasingly recognized as a causal contributor to SCZ pathophysiology [78]. The *MAPT* gene encodes tau, a protein essential for microtubule stabilization and axonal transport [79]. Given tau’s central role across both neuronal and glial compartments, and the established modulation of tau pathology in neurodegenerative disease [80], our findings raise the prospect of targeting astrocytic *MAPT* expression therapeutically to address SCZ-related structural brain abnormalities.

In the context of neurological disorders, three excitatory neuron eGenes—*ZSCAN31* [51,81], *SYT14* [82,83] and *ICA1L* [48,84]—were shared between AD and multiple IDPs (**S8** **and** **S9 Figs**), converging with prior evidence that variants in these genes influence both brain imaging measurements and AD susceptibility [48,51,81–84].

Finally, among behavioral-cognitive phenotypes, *XKR6* in inhibitory neurons was shared across ASP, GRT, 15 brain regional volumes (including right lateral orbitofrontal cortex and left isthmus cingulate), and 5 white matter microstructure phenotypes (including fornix–stria terminalis [FXST], IFO, and uncinate fasciculus [UNC]; **S8** **and** **S9 Figs**). *XKR6* belongs to the Kell blood group complex subunit–related family [85]. Large-scale GWAS of risky behavior have identified *XKR6*-related variants and implicated inhibitory neurotransmission in shaping individual variation in risk tolerance [86]. Separately, astrocytic *SRR* expression was causally linked to smoking cessation (SC), DPW, neuroticism (NEU), 5 brain regional volumes, and one white matter microstructure phenotype. Encoding serine racemase, *SRR* is expressed in excitatory glutamatergic neurons of the human forebrain [87] and plays a key role in glutamatergic synaptic signaling [88]. Its expression has been associated with substance use phenotypes, and *SRR* represents a promising pharmacological target for smoking cessation [88].

### Cell type-informed clustering of phenotypes reveals biologically meaningful patterns

To identify phenotypes with shared cell type–specific regulatory architectures, we computed a phenotype × phenotype Jaccard similarity matrix based on shared causal eGenes and their corresponding cell types (**S1 Text** and **S10 Fig**). Oligodendrocytes, excitatory neurons, and astrocytes were the dominant contributors to the global similarity structure (**S11 Fig**), reflecting their greater overall abundance of phenotype-associated causal eGenes (Fig 1b**-1c**). Hierarchical clustering identified 12 robust phenotype clusters (**S10 Fig** and **S10 Table**), of which eight (C2–C9 and C12) contained both IDP and DB phenotypes (**S12 Fig**). Clusters C6 and C7 were the most heterogeneous, each spanning all five phenotype groups, with inhibitory neurons as the principal cell type driving shared molecular regulation across these diverse phenotypic categories. Cluster C4 was significantly enriched for white matter microstructure phenotypes (FDR = 0.0014, hypergeometric test) and nominally enriched for psychiatric disorders (*P* = 0.03), with microglia as the predominant cell type—implicating microglia-mediated mechanisms in the shared genetic basis of white matter integrity and psychiatric vulnerability.

We next asked whether these molecularly defined clusters mirror clinically observed comorbidity patterns. Focusing on disorder–disorder pairs that fell within the same cluster, we surveyed the literature for documented comorbidities and epidemiological associations (**S11 Table**). All nine psychiatric–psychiatric pairs identified showed established comorbidity support; for example, BIP clustered with both SCZ and anxiety disorder (ANX) [89,90]. Cross-category pairs also displayed compelling clinical parallels. Epilepsy (EPI) and PTSD co-occurred in cluster 6, consistent with documented clinical comorbidity [91]. IS and obsessive-compulsive disorder (OCD) shared cluster 7, supported by longitudinal evidence of elevated stroke risk in OCD patients [92]. PD and alcohol use disorder (AUD) clustered together in C12, aligning with epidemiological findings of increased AUD prevalence among PD patients [93]. Together, these observations indicate that phenotype clusters derived from shared cell type–specific causal eGenes capture not only molecular convergence but also clinically meaningful disease relationships.

### Putative causality routes among cell type-specific causal eGenes and brain-associated complex phenotypes

**Fig 4:**
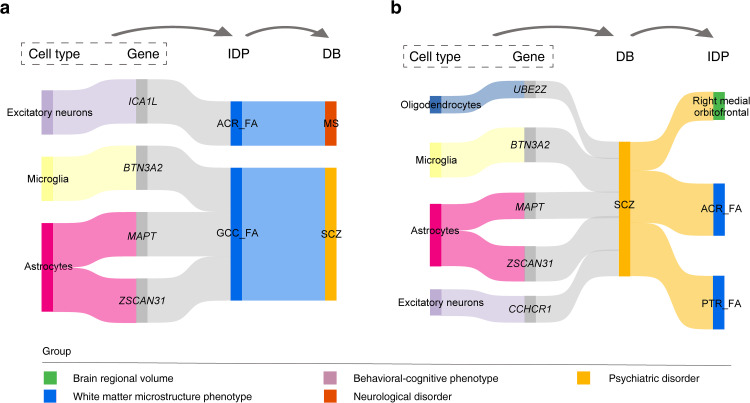
Cell type–specific causal routes linking eGenes to IDPs and DBs. **(a)** eGene–IDP–DB routes: the eGene is causal for both the IDP and the DB, and the IDP is causal for the DB. **(b)** eGene–DB–IDP routes: the eGene is causal for both the IDP and the DB, and the DB is causal for the IDP.

Genetic risk for brain disorders may operate partly through intermediate imaging phenotypes—altering brain structure to influence disease risk, or conversely, manifesting as structural changes downstream of disease processes [20,94,95]. To dissect these causal chains at cellular resolution, we performed bidirectional Mendelian randomization between IDPs and DBs (**Methods**). This identified six causal effects from IDPs to DBs and three from DBs to IDPs. By overlaying these bidirectional phenotype relationships with the cell type–specific eGene–phenotype associations identified above, we reconstructed four eGene–IDP–DB routes and seven eGene–DB–IDP routes in which the eGene was causal for both the imaging phenotype and the disorder (**Fig 4** and **S12 Table**).

One such route links ICA1L expression in excitatory neurons to ACR microstructure and MS (**Fig 4a** and **S12 Table**). *ICA1L* encodes a BAR-domain protein family member implicated in excitatory synaptic signaling [96,97]. Its dysregulation has been tied to MS pathogenesis [98], and variants in this gene are associated with ACR microstructure [19], a tract critical for executive control [99]. Diffusion tensor imaging (DTI) abnormalities of the corona radiata are also documented in MS [100]. Our results position *ICA1L* in excitatory neurons as a upstream regulator: it negatively affects ACR microstructure (SMR: *β* = -0.16, FDR = 5.42 × 10^-4^; Wald ratio: *β* = -0.17, FDR = 1.16 × 10^-36^; GSMR: *β* = -0.15, FDR = 1.91 × 10^-4^), which in turn elevates MS risk (PMR-Egger: *β* = 0.11, FDR = 0.002; GSMR: *β* = 1.06, FDR = 2.16 × 10^-15^). ICA1L also exerts a direct negative causal effect on MS (SMR: *β* = -0.20, FDR = 0.011; Wald ratio: *β* = -0.19, FDR = 1.1 × 10^-6^; GSMR: *β* = -0.19, FDR = 9.63 × 10^-3^), suggesting both indirect and potentially pleiotropic influences on disease risk.

Conversely, an eGene–DB–IDP route connects ZSCAN31 expression in astrocytes to SCZ and posterior thalamic radiation (PTR) microstructure (**Fig 4b** and **S12 Table**). ZSCAN31 encodes a C2H2-type zinc finger protein [101] with established links to both SCZ risk and neuroimaging phenotypes [81]. Thalamic radiation abnormalities are well documented in SCZ [102], and thalamocortical dysconnectivity appears to worsen progressively during psychosis onset [103]. Our results indicate that astrocytic ZSCAN31 expression contributes to the effect of SCZ progression on PTR microstructure (PMR-Egger: *β* = 0.087, FDR = 1.54 × 10^-5^; GSMR: *β* = 0.033, FDR = 1.45 × 10^-5^). ZSCAN31 is itself positively associated with SCZ (Wald ratio: *β* = 0.17, FDR = 2.38 × 10^-26^; GSMR: *β* = 0.14, FDR = 1.53 × 10^-5^; SMR: *β* = 0.16, FDR = 6.89 × 10^-5^) and with PTR microstructure (Wald ratio: *β* = 0.08, FDR = 2.81 × 10^-8^; GSMR: *β* = 0.08, FDR = 2.57 × 10^-3^; SMR: *β* = 0.08, FDR = 0.03), positioning it as a shared astrocytic regulator of both disease risk and structural brain changes.

Together, these integrated causality routes nominate cell type–specific genes through which brain structure and disorder may be causally coupled, offering testable entry points for mechanistic and therapeutic follow-up.

To identify regulatory variants underlying the 11 causality routes (**Fig 4**), we further performed SNP-level fine-mapping, colocalization, and regulatory annotation (**S2 Text**). This yielded 56 SNP–cell type–eGene–phenotype chains, each linking a regulatory variant to its cell type–specific eGene and two associated phenotypes. For example, the chain rs3130455–*CCHCR1* (excitatory neurons)–SCZ–PTR (effect direction: + , + , +) places this variant within an active promoter chromatin state (ChromHMM 1_TssA) across neural lineages, where it disrupts neurodevelopmental transcription factor motifs including Klf4 and Klf7. This supports a model in which increased rs3130455 dosage elevates *CCHCR1* expression in excitatory neurons, raising SCZ risk and enhancing white matter connectivity in the PTR. Another chain, rs55938136–*MAPT* (astrocytes)–GCC–SCZ (effect direction: + , − , +), reveals perfect colocalization of the intronic SNP with both astrocytic *MAPT* expression and genu of corpus callosum (GCC) fractional anisotropy (PP.H4 = 1; PP.H4.snp = 1). This suggests that higher rs55938136 dosage increases astrocytic *MAPT* expression, diminishes GCC structural connectivity, and thereby elevates SCZ susceptibility [104,105]. All 56 chains are catalogued in **S13 Table**.

### Spatiotemporal expression patterns of causal eGenes for IDPs and DBs

**Fig 5:**
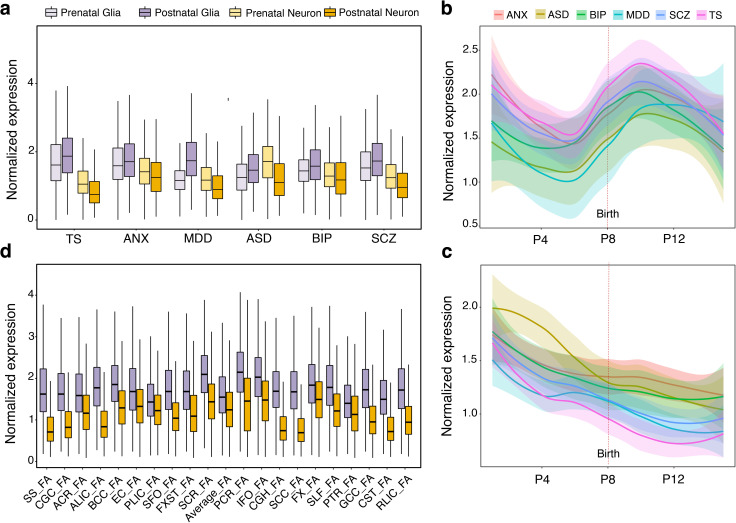
Spatiotemporal expression for causal eGenes of psychiatric disorders and white matter microstructure phenotypes. **(a)** Prenatal versus postnatal expression of psychiatric disorder causal eGenes in glial and neuronal cells. **(b-c)** Lifespan trajectories of psychiatric disorder causal eGenes in (b) glial and (c) neuronal cells; shaded regions, 95% confidence intervals. **(d)** Postnatal expression of white matter microstructure causal eGenes in glial and neuronal cells. Developmental stages: P4, 13 PCW ≤ Age < 16 PCW; P8, Birth ≤ Age < 6 Months; P12, 12 ≤ Age < 20 Years. ANX, anxiety disorder; ASD, autism spectrum disorder; BIP, bipolar disorder; MDD, major depressive disorder; SCZ, schizophrenia; TS, Tourette syndrome. Glia: microglia, astrocytes, oligodendrocytes, and OPCs. Neurons: inhibitory and excitatory neurons.

We next examined the expression patterns of causal eGenes in human brain single-cell data (**Methods**). For DBs, the causal eGenes were markedly enriched in glial cells relative to neurons (*P* < 2.2 × 10^-16^, Wilcoxon test), with the strongest enrichment in astrocytes (**S13 Fig**). Psychiatric and behavioral-cognitive causal eGenes showed higher neuronal expression during the prenatal period than postnatally, whereas glial cells displayed the inverse pattern (*P* < 0.001, Wilcoxon test; **Figs 5a** and **S14a**). Most neurological disorder eGenes were elevated in neural cells after birth; notably, amyotrophic lateral sclerosis (ALS) eGenes were exclusively neuronal and showed postnatal upregulation (**S14b Fig**), consistent with prior observations [106–108].

Across the lifespan, psychiatric disorder eGenes in glial cells declined through late mid-fetal development (19–24 PCW) before rising again in early childhood (1–6 years), while their neuronal expression decreased monotonically (Figs 5b**-5c** and **S15**). Behavioral-cognitive phenotypes followed a similar trajectory (**S16 Fig**), suggesting convergent developmental programs for these related phenotype categories.

For IDPs, causal eGenes for white matter microstructure phenotypes were strongly enriched in adult glial cells—particularly astrocytes and microglia (**Fig 5d**)—mirroring the predominance of glial cells in white matter tissue [109]. These spatiotemporal expression profiles provide an independent biological layer that complements the genetically inferred causal relationships. Whereas the MR framework captures *cis*-eQTL–driven regulatory architecture, the observed cell type–specific and developmental stage–specific transcriptional dynamics offer convergent evidence of biological plausibility, pointing to coordinated regulatory and transcriptional programs underlying brain-associated complex phenotypes.

### Expression of causal eGenes indicates associations of brain-associated complex phenotypes

**Fig 6:**
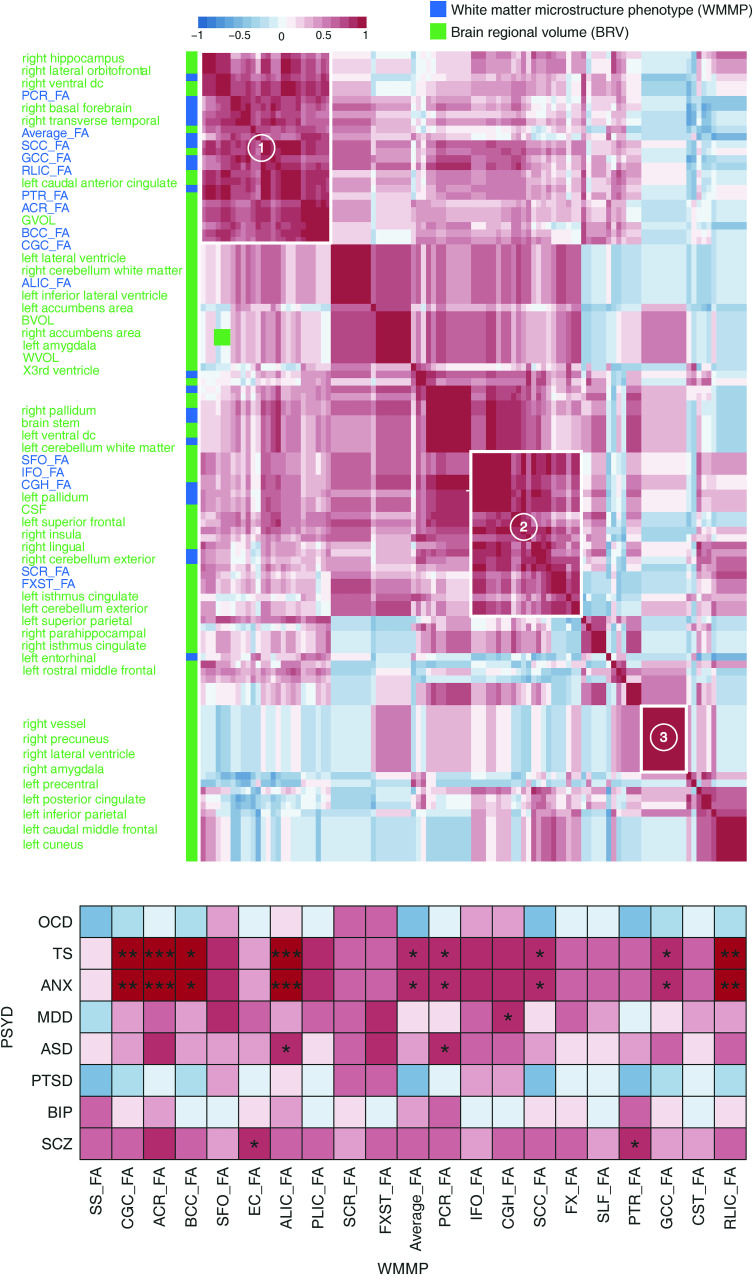
Coordinated phenotype–phenotype associations revealed by causal eGene expression. **(a)** Correlation heatmap among imaging-derived phenotypes (IDPs), with phenotype group membership indicated by color bars (brain regional volumes; white matter microstructure phenotypes). **(b)** Correlation heatmap between white matter microstructure phenotypes and psychiatric disorders. Values are Spearman’s rank correlation coefficients of mean causal eGene expression across eight cell types (**Methods**). *, FDR < 0.05; **, FDR < 0.01; ***, FDR < 0.001.

Brain function relies on the interplay between regional structure and white matter connectivity [110,111], yet how these relationships are manifest at the cellular level remains unclear. To address this, we examined the associations between brain regional volumes and white matter microstructure phenotypes using cell type–specific expression data from the STAB2 [112]. Regional volumes and white matter phenotypes formed largely distinct clusters (**Fig 6a** and **Methods**). Notably, causal eGene expression tracked cerebral hemisphere specificity: homologous regions from opposite hemispheres were assigned to different modules (Spearman’s rank correlation coefficient *r* > 0.7), indicating that genetically driven cell type–specific expression underlies hemispheric structural divergence.

A similar analysis of DBs revealed that causal eGene expression profiles carried functional associations with brain disorders and phenotypes (**S17 Fig**). For instance, the expression signatures of educational attainment and intelligence correlated strongly with those of AD (Spearman’s rank correlation coefficient *r* > 0.9), echoing epidemiological evidence that higher cognitive reserve lowers AD risk [113,114].

These expression patterns also corroborate the causal routes linking IDPs to DBs identified above (**Fig 4**). Fractional anisotropy in the PTR and the inferior fronto-occipital fasciculus (IFO) was significantly associated with SCZ and general risk tolerance (GRT), respectively (**Figs 6b** and **S18**), consistent with the bidirectional causal relationships inferred by MR. Beyond these known links, we detected significant expression associations between several regional volumes and psychiatric disorders (FDR < 0.05, Spearman’s rank correlation test). The strongest was between major depressive disorder (MDD) and the left rostral middle frontal cortex (FDR = 4.01 × 10^-45^, Spearman’s rank correlation test; **S19 Fig**), implicating this prefrontal region in the transcriptional architecture of depression.

## Discussion

In this study, we employed a single-cell Mendelian randomization framework to dissect potential causal relationships between gene expression and brain-associated complex phenotypes, leveraging complementary analytical methods to enhance reliability. We identified 254 and 217 eGenes with putative causal effects on 112 IDPs and 26 DBs, respectively, across eight brain cell types. These causal eGenes exhibited strong cell type specificity: over 90% of eGene–IDP associations and approximately 80% of eGene–DB associations were restricted to a single cell type. At the same time, the causal eGenes showed widespread phenotype pleiotropy, exemplified by *DDHD2* in excitatory neurons, *XKR6* in inhibitory neurons, and *MAPT* and *ZSCAN31* in astrocytes. Genes shared across different categories of brain-associated phenotypes displayed distinct patterns of evolutionary constraint and cell type enrichment, and were overrepresented in biological processes including memory and cognition, neurotransmitter regulation, and cellular maintenance and signaling. We further characterized putative causality routes linking cell type–specific causal eGenes to IDPs and DBs, and, by examining the spatiotemporal expression dynamics of these genes in independent single-cell data, uncovered coordinated transcriptional programs that underpin the relationships among brain-related complex phenotypes.

### Regulation by cell type-specific causal eGenes

Previous studies have pointed to an important yet underrecognized role for excitatory neurons in AD etiology [115,116]. In our analysis, six eGenes—*SYT14*, *MSH3*, *ICA1L*, *RGS14*, *C17orf97* and *ZSCAN31*—were causally associated with AD in excitatory neurons (**S6 Table**). Among these, five (*MSH3*, *ICA1L*, *RGS14*, *C17orf97* and *ZSCAN31*) showed a consistent positive causal effect. Several of these genes have established links to excitatory neuron or synaptic function: *SYT14* mediates calcium-dependent neurotransmitter release and synaptic vesicle trafficking [117,118]; *ICA1L* and *RGS14* modulate synaptic signaling pathways in excitatory neurons [97,119]; and *ZSCAN31*, a zinc finger protein, regulates neuronal excitability and intrinsic circuitry within this subtype [120,121]. This convergence suggests that these genes may influence AD progression through shared mechanisms affecting neuronal function. Moreover, we observed significant overlap between AD and brain structural phenotypes with respect to cell type–specific causal eGenes in excitatory neurons (**Fig 3**), including *ZSCAN31*, *SYT14*, and *ICA1L*. These findings collectively strengthen the mechanistic links among genes, excitatory neurons, AD, and brain structure.

*ZSCAN31* is among the most pervasively pleiotropic genes in our catalog. In astrocytes, it showed a positive causal effect on all 11 associated phenotypes spanning four groups: MDD and SCZ (psychiatric disorders), GRT (behavioral-cognitive phenotypes), right supramarginal volume (brain regional volumes), and seven white matter microstructure phenotypes—mean fractional anisotropy of the GCC (genu of corpus callosum), PTR (posterior thalamic radiation), SLF (superior longitudinal fasciculus), SCC (splenium of corpus callosum), BCC (body of corpus callosum), ACR (anterior corona radiata), and the average across all tracts (**Fig 3b** and **S4 Table**). By contrast, *ZSCAN31* in excitatory neurons exerted a negative causal effect on right hippocampal and total white matter volume. This divergence in effect direction across cell types indicates that *ZSCAN31* is subject to cell type–specific regulatory control over brain-associated phenotypes, extending prior findings [101]. We accordingly inferred three putative routes linking *ZSCAN31* in astrocytes to IDPs and DBs (**Fig 4** and **S12 Table**). For example, the route *ZSCAN31* in astrocytes → SCZ → PTR suggests that astrocytic *ZSCAN31* expression may mediate the influence of SCZ disease progression on PTR microstructural change. Consistent with this, external single-cell data indicated that causal eGenes in multiple cell types participate in the association between SCZ and PTR (**Fig 6b**), illuminating how cell type–specific eGene regulation shapes brain-associated phenotype relationships.

### Shared genetic signals between risky behaviors and IDPs

Our analysis supports genetically driven links between risky behaviors and multiple IDPs at the cellular level. We observed significant sharing of causal eGenes within inhibitory neurons between risky behaviors (GRT and ASP) and both brain regional volumes and white matter microstructure phenotypes (**Fig 3a**). Among these shared signals, *XKR6* in inhibitory neurons was implicated in both risky behaviors (GRT and ASP) and several IDPs, including cortical volumetric phenotypes and white matter phenotypes such as the FXST, IFO and UNC. Using external expression data across cell types, we also detected significant expression-profile correlations between GRT and IFO, and between GRT and FXST (**S18 Fig**), pointing to a mediating role for cell type–specific causal eGenes in these associations. These results suggest a shared cell type–specific genetic architecture linking risky behaviors to selected IDPs. They should not, however, be taken as evidence for the precise brain regions in which these effects operate; that anatomical context awaits further validation.

### Interpretation of cell type-specific associations

The high cell type specificity observed here (91.4% for eGene–IDP pairs and 82.1% for eGene–DB pairs) should be interpreted cautiously. These rates reflect the specificity of significant eGene–phenotype associations after TWAS and MR filtering, not the inherent specificity of eQTL effects. This definitional distinction may partly explain why our estimates exceed those reported in studies focused on cell type–specific eGenes or eQTLs [13,122]. In addition, methodological factors—such as significance filtering (TWAS + MR) and uneven *cis*-eQTL availability across cell types (e.g., the comparatively small number in endothelial cells and pericytes)—may inflate the apparent specificity.

It is also important to note that the *cis*-eQTL data used here were derived from a limited set of brain regions (prefrontal cortex, temporal cortex, and deep white matter) rather than the full complement of anatomical regions represented by the imaging phenotypes. Given the context dependence of molecular eQTL effects and known transcriptomic divergence across brain regions—especially between cortical and subcortical structures [44]—associations involving unmatched regions should be treated with caution. More broadly, MR captures the effect of a genetically proxied expression perturbation and does not identify the exact biological context or site of action [42]. Our results are therefore best regarded as prioritizing putative cell type–specific genes for brain phenotypes, rather than pinpointing the precise brain regions in which each regulatory effect occurs.

### Limitations

Several avenues remain open for future work. First, the comparatively small number of *cis*-eQTLs in endothelial cells and pericytes (**S3 Table**) [12], likely constrained the number of causal genes identified in these cell types (**Fig 1b**). Larger single-cell eQTL datasets with greater sample sizes and finer cell type resolution should improve power to detect eGene–phenotype associations in these populations. Second, when performing MR between IDPs and DBs, half of the DB GWASs were excluded because they included participants from UK Biobank [123], limiting the completeness of the inferred causality routes among eGenes, IDPs, and DBs (**Fig 4**). In addition, shared causal eGenes identified across IDPs and/or DBs may be influenced by unavoidable sample overlap between some GWAS datasets. Future studies should assemble more fully independent GWAS resources for brain-associated phenotypes to strengthen the robustness and coverage of these causal inferences. Third, although our findings highlight putative causal relationships, they do not establish definitive causality and require confirmation by randomized controlled trials, the gold standard for causal inference. Considerable work remains ahead in treatment development, and our results should be interpreted accordingly.

### Outlook

In summary, our study provides a systematic investigation of cell type–specific causal genes for brain-associated complex phenotypes, together with evidence that brain structure and disorders/behaviors share genetic foundations operating at the cellular level. We hope that the connections identified among genes, cell types, and complex phenotypes will serve as starting points for developing therapeutic strategies targeting brain structural abnormalities, disorders, and behaviors.

## Methods

### Cell type-specific *cis*-eQTL summary statistics

We obtained published cell type–specific *cis*-eQTL summary statistics comprising effect sizes for 67,815,924 SNP–gene pairs across 4,690,822 SNPs and 17,881 gene transcripts [12]. The data cover eight major brain cell types: astrocytes, excitatory neurons, inhibitory neurons, oligodendrocytes, OPCs, microglia, endothelial cells, and pericytes (**Fig 1a** and **S3 Table**). The *cis*-eQTLs were identified by integrating high-quality single-nuclei RNA sequencing data from the prefrontal cortex, temporal cortex, and white matter with corresponding genotype data from 192 individuals. All individuals fell within three standard deviations of the mean for the first and second principal components of European-ancestry populations from the 1000 Genomes Project [12].

### GWAS summary statistics for brain disorders and behaviors

We obtained publicly available GWAS summary statistics for 26 brain DBs, including eight behavioral-cognitive phenotypes, eight neurological disorders, and 10 psychiatric disorders (**S1 Table**). Most were published recently and derived from meta-analyses with large sample sizes (mean ~280,000 individuals). All GWAS summary statistics were of European ancestry, and there was no participant overlap with the cell type–specific *cis*-eQTL dataset.

The sample size for each GWAS summary statistics dataset is listed below.

- Behavioral-cognitive phenotypes
  (1) Automobile speeding propensity (ASP): 404,291 individuals (heritability: 7.9%) [86]
  (2) Childhood intelligence (CI): 12,441 individuals (heritability: 22%-46%) [124]
  (3) Drinks per week (DPW): 941,280 individuals (heritability: 4%) [125]
  (4) Educational attainment (EA): 766,345 individuals (heritability: 12.2%) [126]
  (5) General risk tolerance (GRT): 466,571 individuals (heritability: 4.6%) [86]
  (6) Intelligence (INT): 269,867 individuals (heritability: 19%) [127]
  (7) Neuroticism (NEU): 390,278 individuals (heritability: 10%) [128]
  (8) Smoking cessation (SC): 547,219 individuals (heritability: 5%) [125]
- Neurological disorders
  (1) Alzheimer’s disease (AD): 71,880 cases and 383,378 controls (heritability: 5.5%) [129]
  (2) Amyotrophic lateral sclerosis (ALS): 20,806 cases and 59,804 controls (heritability: not reported in the original GWAS publication) [130]
  (3) Epilepsy (EPI): 15,212 cases and 29,677 controls (heritability: 9.7%) [131]
  (4) Intracerebral hemorrhage (ICH): 1,545 cases and 1,481 controls (heritability: not reported in the original GWAS publication) [132]
  (5) Insomnia (INS): 109,548 cases and 277,440 controls (heritability: 7.2%) [133]
  (6) Ischemic stroke (IS): 67,162 cases and 454,450 controls (heritability: not reported in the original GWAS publication) [134]
  (7) Multiple sclerosis (MS): 47,429 cases and 68,374 controls (heritability: 19.2%) [135]
  (8) Parkinson’s disease (PD): 33,674 cases and 449,056 controls (heritability: 22%-27%) [136]
- Psychiatric disorders
  (1) Attention-deficit/hyperactivity disorder (ADHD): 20,183 cases and 35,191 controls (heritability: 21.6%) [137]
  (2) Anxiety disorder (ANX): 25,453 cases and 58,113 controls (heritability: 26%) [138]
  (3) Autism spectrum disorder (ASD): 18,381 cases and 27,969 controls (heritability: 11.8%) [139]
  (4) Alcohol use disorder (AUD): 11,569 cases and 34,999 controls (heritability: 9.0%) [140]
  (5) Bipolar disorder (BIP): 41,917 cases and 371,549 controls (heritability: 18.6%) [141]
  (6) Major depressive disorder (MDD): 135,458 cases and 344,901 controls (heritability: 8.7%) [142]
  (7) Obsessive-compulsive disorder (OCD): 2,688 cases and 7,037 controls (heritability: 28%) [143]
  (8) Post-traumatic stress disorder (PTSD): 9,831 cases and 19,225 controls (heritability: 28%) [144]
  (9) Schizophrenia (SCZ): 76,755 cases and 243,649 controls (heritability: 24%) [145]
  (10) Tourette syndrome (TS): 4,819 cases and 9,488 controls (heritability: 21%) [146]

### GWAS summary statistics for imaging-derived phenotypes

We obtained publicly available GWAS summary statistics for 123 brain IDPs, including 101 brain regional (and total) volume phenotypes and 22 white matter microstructure phenotypes (**S2 Table**). The brain volume GWAS summary statistics were processed by Zhao et al. [147] from 19,629 UK Biobank participants of European ancestry (reported mean heritability ~40%). Volumes included total brain volume, gray matter, white matter, and cerebrospinal fluid, labeled with the Mindboggle-101 atlas [148]. The white matter tract GWAS summary statistics were processed by Zhao et al. [22] from 33,292 UK Biobank participants of European ancestry (reported mean heritability ~46.3%). Tracts were segmented with the JHU ICBM-DTI-81 white-matter atlas [149–151], and microstructure was quantified by mean fractional anisotropy (FA).

### Transcriptome-wide association study

To investigate potential links between gene expression and brain-associated complex phenotypes, we conducted a TWAS for the GWAS summary statistics of 123 IDPs and 26 DBs [31]. Prediction weights and covariance were obtained from two resources: (1) PredictDB Data Repository (https://predictdb.org/), where prediction models were built from GTEx V8 expression data [152] across 13 brain regions (amygdala, anterior cingulate cortex [BA24], caudate, cerebellar hemisphere, cerebellum, cortex, frontal cortex [BA9], hippocampus, hypothalamus, nucleus accumbens, putamen, spinal cord [cervical C-1] and substantia nigra); (2) CMC-derived DLPFC prediction models (https://github.com/laurahuckins/CMC_DLPFC_prediXcan), built from CommonMind Consortium expression data [153] from dorsolateral prefrontal cortex (DLPFC) tissue. TWAS was implemented with MetaXcan [31]. eGene–phenotype reaching nominal significance (*P* < 0.05) in at least one brain tissue were retained for subsequent MR analysis.

### Primary Mendelian randomization analysis

To investigate the causal effects of cell type–specific gene expression on brain-associated complex phenotypes, we performed two-sample MR using four complementary methods: two single-SNP methods (SMR [6] and Wald ratio [32]) and two multi-SNP methods (PMR-Egger [8] and GSMR [33]). Details on each method are provided below (**S1 Fig**).

(1) The SMR method tests whether the expression level of a gene is associated with a phenotype under the assumption of either causality or pleiotropy [6]. It is designed specifically for gene expression as the exposure and requires only one SNP as the instrument. The top *cis*-eQTL associated with each gene expression in a given cell type at genome-wide significance (*P* < 5 × 10^-8^) was selected as the instrument. SMR incorporates the HEIDI test to detect linkage [6]; results with HEIDI *P* < 0.05 were discarded. SMR and the HEIDI test were implemented with SMR software (version 1.3.1, https://yanglab.westlake.edu.cn/software/smr).
(2) The Wald ratio method computes the change in disease risk per standard deviation change in gene expression, using the instrumental *cis*-eQTL for the target gene. It is applicable to any exposure and requires only one SNP as the instrument under the standard MR assumptions (relevance, independence, and exclusion restriction). For instrument selection, LD clumping (*r*^*2*^ < 0.1, window = 250 kb, 1000 Genomes EUR reference panel) was first performed for *cis*-eQTLs associated with each gene expression at genome-wide significance (*P* < 5 × 10^-8^) using PLINK v1.90 [154]. The independent *cis*-eQTL(s) for each gene in the given cell type were then selected as instrument(s). After instrument selection, 86.6% of cell type–specific genes had one valid instrumental *cis*-eQTL, so the Wald ratio could be applied in most analyses. For the remaining 13.4% of genes with more than one valid instrument, the inverse-variance weighted (IVW, fixed-effects) method was used as a supplementary approach [155], which is commonly paired with the Wald ratio in the literature [20,156]. Both methods were implemented with the R package *TwoSampleMR* (version 0.5.10) [157]. The *harmonise_data()* function in TwoSampleMR was used to harmonize effect alleles and SNP effects between exposure and outcome. Heterogeneity (function *mr_heterogeneity*, *P* < 0.05) and pleiotropy (function *mr_pleiotropy_test*, *P* < 0.05) tests were conducted for IVW results.
(3) The PMR-Egger method estimates the causal effect of gene expression on a phenotype in the presence of horizontal pleiotropy within an MR likelihood framework [8]. It is designed specifically for gene expression as the exposure, can handle multiple correlated variants as instruments, and accounts for linkage disequilibrium (LD) among them. The *cis*-eQTLs (clumping *r*^*2*^ < 0.9, window = 250 kb, 1000 Genomes EUR reference panel) associated with each gene expression in a given cell type at genome-wide significance (*P* < 5 × 10^-8^) were selected as instruments. PMR-Egger was implemented with the R package *PMR* (version 1.0). The LD correlation matrix was derived from the 1000 Genomes EUR reference panel, and the maximum number of iterations was set to 5000.
(4) The GSMR method extends SMR to estimate the causal effect of an exposure on an outcome, leveraging multiple variants while accounting for LD among them [33]. It is applicable to any exposure under the standard MR assumptions. The *cis*-eQTLs (clumping *r*^*2*^ < 0.9, 1000 Genomes EUR reference panel) associated with each gene expression in a given cell type at genome-wide significance (*P* < 5 × 10^-8^) were selected as instruments. GSMR incorporates the HEIDI-outlier test to identify and exclude instruments with significant pleiotropic effects on the outcome (*P* < 0.05). Both GSMR and the HEIDI-outlier test were implemented with the R package *gsmr* (version 1.1.0).

A gene was deemed putatively causal for a phenotype within a given cell type (cell type–specific causal eGene) if it met the following criteria: (1) significant in at least one single-SNP method after FDR correction (FDR < 0.05) [158]; (2) significant in at least one multi-SNP method after FDR correction (FDR < 0.05); (3) consistent causal effect direction across all four methods, reducing the likelihood of false positives.

We also calculated the F-statistic for each instrument using the Cragg-Donald statistic to assess instrument strength [159] by $F=R^{\text{2}}\times \frac{N-\text{1}-k}{(\text{1}-R^{\text{2}})\times k}$, where $R^{\text{2}}=\frac{\beta^{\text{2}}}{\beta^{\text{2}}+se^{\text{2}}\times N}$ represents the proportion of exposure variance explained by the instrument, *N* is the sample size from the *cis*-eQTL summary statistics, *β* is the effect size, *se* is the standard error, and *k* is the number of instruments used in the MR estimate (*k* = 1 for a single instrument). Genes for which the averaged F-statistic across all instruments exceeded 10 were retained. A brief introduction to MR for non-expert readers is provided in **S3 Text**.

### Significance of cell type-specific causal eGenes sharing

We applied a hypergeometric test to assess whether cell type–specific causal eGenes were significantly shared between any two brain-associated complex phenotypes. The background gene set was derived by multiplying the total number of genes (only those with the same biotypes as the causal eGenes) by the total number of cell types. When estimating overlap within a particular cell type, the background comprised the total number of genes with the same biotypes as the causal eGenes. An FDR threshold of 0.05 was used to select significant cell types. Gene annotation was obtained from Ensembl (GRCh37 release 87) [160].

### Gene set enrichment analysis

We utilized all gene sets from the Biological Process (BP), Molecular Function (MF), and Cellular Component (CC) sub-ontologies in the Gene Ontology (GO) database for gene set enrichment analysis [161,162]. GO enrichment was implemented with the *enrichGO()* function in the R package *clusterProfiler* (version 4.10.0) [57].

### Replication of predicted associations

We performed replication analysis on the predicted causal associations between eGenes and brain-associated complex phenotypes identified in our MR analysis. To validate eGene–phenotype pairs, three external datasets were used. (1) The *cis*-eQTL summary statistics from the BrainMeta portal (https://yanglab.westlake.edu.cn/data/brainmeta/cis_eqtl/), derived from RNA sequencing of 2,865 brain cortex samples from 2,443 unrelated European-ancestry individuals [36]. The same MR analysis procedure was applied to infer causal relationships between cortical gene expression and 149 brain-associated complex phenotypes. An eGene–phenotype pair was considered replicated if it achieved nominal significance in at least one MR method and showed consistent effect directions across all MR methods and with the discovery analysis. (2) The differentially expressed genes and transcripts (at 5% FDR) in ASD, SCZ and BIP from PsychENCODE [29], which used in vivo gene expression profiles to identify transcriptome-wide isoform alterations across the three major psychiatric disorders. (3) Published eGene–phenotype associations from the NHGRI-EBI GWAS Catalog (version 2023-06-09, www.ebi.ac.uk/gwas/) [1].

To validate eGene–cell type–phenotype triples, two external datasets were used: (1) cell type–specific eQTL summary statistics from the brainSCOPE resource (https://brainscope.gersteinlab.org/integrative_files.html), generated from single-cell resolution data of 388 adult DLPFC samples [37]. Six major cell types were considered: astrocytes, excitatory neurons, inhibitory neurons, oligodendrocytes, OPCs, and microglia. The same MR analysis procedure was applied to infer causal relationships between cell type–specific gene expression and 149 brain-associated complex phenotypes. An eGene–cell type–phenotype triple was considered replicated if it achieved nominal significance in at least one MR method and showed consistent effect directions across all MR methods and with the discovery analysis. (2) Differentially expressed genes associated with AD pathology in each cell type from Mathys et al. [45]. For each external dataset, the replication rate was calculated as the number of replicated associations divided by the total number tested, and significance was assessed by hypergeometric test. Replication was evaluated only for phenotypes present in both the discovery and replication studies.

### Mendelian randomization between brain IDPs and DBs

To characterize putative causality routes among cell type–specific genes, IDPs, and DBs, we performed bidirectional MR between DBs and IDPs. Because the IDP GWAS was conducted in UK Biobank participants, we selected DB GWAS datasets that did not include UK Biobank samples to minimize sample overlap. Thirteen DB GWASs were retained: CI, EPI, ICH, IS, ALS, MS, OCD, TS, ADHD, AUD, ASD, PTSD, and SCZ. Unlike MR with gene expression as the exposure—where a single independent genome-wide significant *cis*-eQTL typically serves as the instrument—analyses with DBs or IDPs as exposures often involve multiple independent instruments. We therefore primarily employed multi-SNP MR methods: PMR-Egger, GSMR, and methods from the R package *TwoSampleMR* (IVW [155], MR-Egger [163], Weighted median [164], Simple mode [165], Weighted mode [166]). or instrument selection, variants were selected at genome-wide significance (*P* < 5 × 10^-8^) with clumping *r*^*2*^ < 0.6 for PMR-Egger and *r*^*2*^ < 0.1 for GSMR and TwoSampleMR methods. Heterogeneity (function *mr_heterogeneity*, *P* < 0.05) and pleiotropy (function *mr_pleiotropy_test*, *P* < 0.05) tests were conducted for TwoSampleMR results. A phenotype was deemed putatively causal for another if it met the following criteria: (1) significant in at least two MR methods after FDR correction (FDR < 0.05); (2) consistent causal effect direction across all methods.

### Expression analysis of causal eGenes using single-cell data

External normalized single-cell gene expression data were obtained from STAB2 [112]. We extracted cell type–specific expression data of the causal eGenes for the eight cell types used in this study: microglia, astrocytes, oligodendrocytes, OPCs, inhibitory neurons, excitatory neurons, endothelial cells, and pericytes. To assess associations between and within IDPs and DBs at the cell type level, we first compiled gene expression data of the causal eGenes for each IDP or DB in each cell type. For each IDP and DB, we then created an eight-dimensional expression vector, in which each value represented the mean expression of the causal eGenes in a given cell type. Spearman’s rank correlation coefficient was used to evaluate associations between IDPs and DBs based on the expression profiles of the corresponding causal eGenes across cell types. Additionally, hierarchical clustering (complete linkage) was employed to investigate associations of IDPs and DBs based on gene expression correlation coefficients.

## Supporting information

S1 Text Phenotype similarity matrix construction and clustering analysis. (DOCX)

S2 Text SNP-level colocalization and regulatory annotation. (DOCX)

S3 Text Overview of Mendelian randomization and causal inference framework in this study. (DOCX)

S1 Fig Study workflow for the primary Mendelian randomization analysis. IV: instrumental variables.
(TIF)

S2 Fig Replication results for predicted eGene–IDP and eGene–DB associations. Reproducibility of eGene–IDP pairs was assessed using cortical *cis*-eQTL summary statistics from BrainMeta [36]. Reproducibility of eGene–DB pairs was assessed using cortical *cis*-eQTLs from BrainMeta [36], differentially expressed genes from PsychENCODE [29], and eGene–phenotype associations from the GWAS Catalog [1]. Significance of overlap was evaluated by hypergeometric test.
(TIF)

S3 Fig Replication results for predicted eGene–cell type–IDP and eGene–cell type–DB associations. Reproducibility of eGene–cell type–IDP triples was assessed using cell type–specific eQTL summary statistics from brainSCOPE [37]. Reproducibility of eGene–cell type–DB triples was assessed using cell type–specific eQTLs from brainSCOPE [37] and differentially expressed genes from Mathys et al. [45] (only AD-associated causal eGenes were tested). Significance of overlap was evaluated by hypergeometric test.
(TIF)

S4 Fig Distribution of cell type specificity for predicted eGene–phenotype associations. Bar plots show the number of eGene–phenotype associations predicted in exactly *k* cell types (x-axis), restricted to eGenes with valid instrumental *cis*-eQTLs in two or more cell types. The y-axis indicates the number of predicted eGene–phenotype associations detected in exactly *k* cell types.
(TIF)

S5 Fig Overlap of causal eGenes between each pair of phenotype groups. Overlaps were assessed by hypergeometric test. BRV: brain regional volume; WMMP: white matter microstructure phenotype; BCP: behavioral–cognitive phenotype; NEUD: neurological disorder; PSYD: psychiatric disorder.
(TIF)

S6 Fig Percentage and count of shared cell type–specific causal eGenes between different phenotype groups. **(a)** Percentage and count of shared cell type–specific causal eGenes between the brain regional volume group and white matter microstructure phenotypes. **(b)** Percentage and count of shared cell type–specific causal eGenes between the brain regional volume group and DBs.
(TIF)

S7 Fig Genetic correlation and causal eGene overlap among IDPs. **(a)** Pairwise genetic correlations (*rg*) were estimated for all 123 IDPs using LD Score Regression based on GWAS summary statistics [167]. Correlations range from −0.83 to 1. The strongest correlation between IDPs from different groups was observed between the fractional anisotropy of the posterior limb of the internal capsule (PLIC_FA) and the volume of the right lateral ventricle (*r*_*g*_ = 0.49). **(b)** Relationship between pairwise genetic correlation and overlap of cell type–specific causal eGenes among IDPs. The x-axis shows absolute pairwise genetic correlation (|*rg*|), and the y-axis shows the number of overlapping cell type–specific causal eGenes identified by MR analysis. Each point represents one IDP pair, colored by pair type: between-group (one brain regional volume and one white matter microstructure phenotype), within-BRV (both brain regional volumes), or within-WMMP (both white matter microstructure phenotypes). The black curve shows a LOESS-smoothed trend, with the shaded area indicating the 95% confidence interval.
(TIF)

S8 Fig Cell type–specific causal eGenes shared between white matter microstructure phenotypes and behavioral–cognitive phenotypes (a), neurological disorders (b), and psychiatric disorders (c). (TIF)

S9 Fig Cell type–specific causal eGenes shared between brain regional volumes and behavioral–cognitive phenotypes (a), as well as neurological disorders (b). (TIF)

S10 Fig Hierarchical clustering of the phenotype similarity evaluated based on the shared cell type–specific causal eGenes. For each cell type, we constructed a phenotype × phenotype Jaccard similarity matrix based on shared causal eGenes. These were averaged to generate a phenotype similarity matrix, followed by hierarchical clustering. The resulting 12 phenotype clusters (C1–C12) are annotated on the heatmap.
(TIF)

S11 Fig Cell type contributions to the phenotype similarity. Bar plot showing the Pearson correlation between each cell type–specific phenotype similarity matrix and the phenotype similarity matrix. Higher correlations indicate greater contributions of the corresponding cell type to the global phenotype similarity pattern.
(TIF)

S12 Fig Phenotype group enrichment and dominant cell type per cluster. Bar plot showing the enrichment ratio of each phenotype group within the 12 identified phenotype clusters. Significance level is indicated (* FDR < 0.05, # P < 0.05; hypergeometric test). The dominant cell type contributing to each cluster’s similarity structure (defined as having the highest correlation with the phenotype similarity matrix within that cluster) is shown on the left.
(TIF)

S13 Fig Expression levels of the causal eGenes of 26 DBs from glial and neural cells. **(a)** Expression differences in glial and neural cell types for various DBs, with significance indicated as ***, *P* < 0.001. **(b)** Detailed expression profiles of causal eGenes for 26 DBs in glial and neural cell types. Glia: glial cells, including astrocytes, microglia, oligodendrocytes, and OPCs. Neuro: neuronal cells, including inhibitory and excitatory neurons.
(TIF)

S14 Fig Spatiotemporal expression levels of the causal eGenes of behavioral–cognitive phenotypes (a) and neurological disorders (b). Glia: glial cells, including astrocytes, microglia, oligodendrocytes, and OPCs. Neuro: neuronal cells, including inhibitory and excitatory neurons. * *P* < 0.05; ** *P* < 0.01; **** *P* < 0.001.
(TIF)

S15 Fig Expression dynamics of the causal eGenes of psychiatric disorders in astrocytes (a), oligodendrocytes (b), microglia (c), OPCs (d), inhibitory neurons (e) and excitatory neurons (f). Locally estimated scatterplot smoothing (LOESS) curves are shown with 95% confidence intervals. P1, 4 ≤ age < 8 PCW; P2, 8 ≤ age < 10 PCW; P3, 10 ≤ age < 13 PCW; P4, 13 ≤ age < 16 PCW; P5, 16 ≤ age < 19 PCW; P6, 19 ≤ age < 24 PCW; P7, 24 ≤ age < 38 PCW; P8, 0 ≤ age < 6 months; P9, 6 ≤ age < 12 months; P10, 1 ≤ age < 6 years; P11, 6 ≤ age < 12 years; P12, 12 ≤ age < 20 years; P13, 20 ≤ age < 40 years; P14, 40 ≤ age < 60 years; P15, > 60 years.
(TIF)

S16 Fig Expression dynamics of the causal eGenes of behavioral–cognitive phenotypes in astrocytes (a), oligodendrocytes (b), microglia (c), OPCs (d), inhibitory neurons (e) and excitatory neurons (f). For the locally estimated scatterplot smoothing (LOESS) plots, smooth curves are shown with 95% confidence intervals. P1, 4 ≤ Age < 8 PCW; P2, 8 ≤ Age < 10 PCW; P3, 10 ≤ Age < 13 PCW; P4, 13 ≤ Age < 16 PCW; P5, 16 ≤ Age < 19 PCW; P6, 19 ≤ Age < 24 PCW; P7, 24 ≤ Age < 38 PCW; P8, 0 ≤ Age < 6 Months; P9, 6 ≤ Age < 12 Months; P10, 1 ≤ Age < 6 Years; P11, 6 ≤ Age < 12 Years; P12, 12 ≤ Age < 20 Years; P13, 20 ≤ Age < 40 Years; P14, 40 ≤ Age < 60 Years; P15, > 60 Years.
(TIF)

S17 Fig Heatmap showing the expression associations of the eGenes for different groups of DBs. Associations were evaluated based on expression correlation (Spearman’s rank correlation coefficient) of causal eGenes across eight cell types (**Methods**), and clusters were derived using hierarchical clustering (complete linkage).
(TIF)

S18 Fig Heatmap showing the expression associations of the eGenes for DBs and white matter microstructure phenotypes. Associations were evaluated based on expression correlation (Spearman’s rank correlation coefficient) of causal eGenes across eight cell types (**Methods**). * FDR < 0.05; ** FDR < 0.01; *** FDR < 0.001.
(TIF)

S19 Fig Heatmap showing the expression associations of the eGenes for DBs and brain regional volumes. Associations were evaluated based on expression correlation (Spearman’s rank correlation coefficient) of causal eGenes across eight cell types (**Methods**). * FDR < 0.05; ** FDR < 0.01; *** FDR < 0.001.
(TIF)

S1 Table GWAS summary statistics for disorders and behaviors (DBs) used in this study. (XLSX)

S2 Table GWAS summary statistics for imaging-derived phenotypes (IDPs) used in this study. (XLSX)

S3 Table Summary of the cell type–specific *cis*-eQTL dataset used in this study. (XLSX)

S4 Table Summary of significant MR results for cell type–specific causal eGenes on imaging-derived phenotypes (IDPs). (XLSX)

S5 Table Replication results for predicted eGene–IDP and eGene–cell type–IDP associations. (XLSX)

S6 Table Summary of significant MR results for cell type–specific causal eGenes on disorders and behaviors (DBs). (XLSX)

S7 Table Replication results for predicted eGene–DB and eGene–cell type–DB associations. (XLSX)

S8 Table Cell type–specific causal eGenes shared across different phenotype groups. (XLSX)

S9 Table Enriched gene sets of genes shared across phenotype groups and classes (pairwise sharing). (XLSX)

S10 Table Phenotype composition of each of the 12 clusters derived from hierarchical clustering of phenotype similarity. (XLSX)

S11 Table Literature support for disorder–disorder pairs co-clustered based on shared cell type–specific causal eGenes. (XLSX)

S12 Table Putative routes among cell type–specific causal eGenes, IDPs, and DBs. (XLSX)

S13 Table Putative SNP–cell type–specific eGene–phenotype1 (IDP or DB)–phenotype2 (DB or IDP) chains. (XLSX)

S1 STROBE Checklist STROBE-MR checklist of recommended items to address in reports of Mendelian randomization studies [168,169]. (DOCX)
