# Supplementary material for: Deciphering cell type-specific causal genetic effects on brain imaging-derived phenotypes and disorders with single-cell Mendelian randomization
Source: PLoS Comput Biol. 2026 Jun 17;22(6):e1014422. doi: 10.1371/journal.pcbi.1014422 (PMC13289931; doi:10.1371/journal.pcbi.1014422)
Supplement: S1 Text — (DOCX) [file pcbi.1014422.s001.docx]

# S1 Text. Phenotype similarity matrix construction and clustering analysis

For each brain cell type, we constructed a phenotype × phenotype Jaccard similarity matrix from the predicted causal eGenes. The Jaccard index for each phenotype pair was calculated as the number of shared causal eGenes divided by the size of the union of their causal eGene sets. These cell type–specific matrices were then averaged to produce an aggregate similarity matrix representing global phenotype similarity across cell types. To evaluate which cell types chiefly drive this global similarity pattern, we computed the Pearson correlation between each cell type–specific matrix and the aggregate matrix.

Hierarchical clustering was next performed on the aggregate similarity matrix using the R package *pvclust* (version 2.2) with average linkage and correlation distance^1^. Cluster stability was assessed through 10,000 bootstrap replicates, and clusters with approximately unbiased P-values ≥ 0.95 were considered significant. To evaluate which cell types chiefly drive the within-cluster similarity pattern, we calculated the Pearson correlation between each cell type–specific matrix and the aggregate matrix, restricting the calculation to phenotypes within the given cluster. We further tested phenotype group enrichment within clusters using hypergeometric tests and corrected for multiple testing using the false discovery rate (FDR).

# References

1 Suzuki, R. & Shimodaira, H. Hierarchical clustering with P-values via multiscale bootstrap resampling. *R package* (2013).
