# Supplementary material for: Deciphering cell type-specific causal genetic effects on brain imaging-derived phenotypes and disorders with single-cell Mendelian randomization
Source: PLoS Comput Biol. 2026 Jun 17;22(6):e1014422. doi: 10.1371/journal.pcbi.1014422 (PMC13289931; doi:10.1371/journal.pcbi.1014422)
Supplement: S2 Text — (DOCX) [file pcbi.1014422.s002.docx]

# S2 Text. SNP-level colocalization and regulatory annotation

We performed a targeted variant-level analysis on 11 cell type–specific eGene–Phenotype1 (IDP or DB)–Phenotype2 (DB or IDP) routes highlighted in our main results (**Fig. 4**; **S12 Table**). Specifically, we reconstructed the full causal chain from SNP to brain-associated complex phenotypes through the following steps:

(1) Phenotype pair filtering via clustering. We first selected Phenotype1–Phenotype2 pairs that co-clustered in our aggregate phenotype similarity matrix (**S10 Fig**), indicating that they are regulated by a common set of cell type–specific eGenes. Notably, nearly all selected pairs in the 11 routes (e.g., GCC_FA–SCZ, ACR_FA–SCZ, SCZ–right medial orbitofrontal) resided in the same cluster (Cluster C4), supporting their convergence at the gene–cell type level.

(2) Fine-mapping and colocalization. For each cell type–specific eGene–Phenotype1 combination in the selected routes, we first fine-mapped both the corresponding eQTL and Phenotype1 GWAS loci using the SuSiE algorithm (R package *susieR*, version 0.14.2), which permits modeling of multiple causal variants per region^1^. We then applied the function *coloc.susie* from the R package *coloc* (version 6.0.0) to perform colocalization using the posterior distributions from fine-mapping^2^. Loci with posterior probability (PP.H4) exceeding 0.8 were considered colocalized. From these, SNPs with individual-level posterior probability (PP.H4.snp) greater than 0.01 were retained as putative functional variants for further annotation. This yielded a set of SNP–eGene–cell type–Phenotype1 combinations likely to reflect mechanistic links.

(3) Annotation of functional SNPs. We annotated these colocalized SNPs to regulatory features—including chromatin states, DNase hypersensitivity sites, and transcription factor motif disruptions—using *HaploReg* (version 4.2)^3^. This allowed us to link SNPs to potential regulatory elements and complete the chain: SNP (annotated regulatory element) → cell type–specific eGene → Phenotype1 → Phenotype2.

# References

1 Wang, G., Sarkar, A., Carbonetto, P. & Stephens, M. A simple new approach to variable selection in regression, with application to genetic fine mapping. *J R Stat Soc Series B Stat Methodol* **82**, 1273-1300, doi:10.1111/rssb.12388 (2020).

2 Wallace, C. A more accurate method for colocalisation analysis allowing for multiple causal variants. *PLoS Genet* **17**, e1009440, doi:10.1371/journal.pgen.1009440 (2021).

3 Ward, L. D. & Kellis, M. HaploReg: a resource for exploring chromatin states, conservation, and regulatory motif alterations within sets of genetically linked variants. *Nucleic Acids Res* **40**, D930-934, doi:10.1093/nar/gkr917 (2012).
