# Supplementary material for: Deciphering cell type-specific causal genetic effects on brain imaging-derived phenotypes and disorders with single-cell Mendelian randomization
Source: PLoS Comput Biol. 2026 Jun 17;22(6):e1014422. doi: 10.1371/journal.pcbi.1014422 (PMC13289931; doi:10.1371/journal.pcbi.1014422)
Supplement: S3 Text — (DOCX) [file pcbi.1014422.s003.docx]

# S3 Text. Overview of Mendelian Randomization and causal inference framework in this study

## Overview of Mendelian Randomization

A key challenge in human genetics is determining whether a biological factor causes a disease or is merely correlated with it. In observational studies, exposures (i.e., risk factors) and outcomes are often influenced by confounding factors such as age, lifestyle, environment, or cellular composition, which obscure the true direction of causality.

Randomized controlled trials (RCTs) are considered to be the gold standard for testing causality. However, RCTs are often impractical because of ethical constraints. As an alternative, Mendelian randomization (MR) introduces the concept of instrumental variables (IVs) from econometrics and uses genetic variants as IVs to estimate the causal effect of an exposure (e.g., gene expression) on an outcome (e.g., disease or behavior). Because genetic variants are fixed at conception and largely unaffected by environmental influences, they provide a source of variation that is naturally independent of confounding factors^1^. Therefore, if genetically predicted exposure variation is associated with an outcome, it supports a putative causal effect of the exposure on the outcome.

Effective MR methods generally rely on three core assumptions: (i) Relevance: each genetic variant is associated with the exposure; (ii) Independence: each genetic variant is independent of confounding factors; (iii) Exclusion restriction: each genetic variant influences the outcome only through the exposure. For methods such as MR-Egger that explicitly model horizontal pleiotropy (variants influencing the outcome directly), the third assumption is relaxed and replaced by the weaker InSIDE (Instrument Strength Independent of Direct Effect) condition^2^. Because violations of these assumptions can bias causal estimates, MR analyses typically include tests for horizontal pleiotropy, weak instrument bias, and heterogeneity to ensure robustness^2-5^. Provided the genetic instruments satisfy the three core IV assumptions and an additional point-estimate-identifying assumption (e.g., the genetic variant affects the exposure in the same direction across all individuals, typically assumed in standard MR models), the null hypothesis of no causal effect can be tested validly and the resulting exposure–outcome relationship is biologically interpretable^1^.

Whether to select independent SNPs or correlated SNPs as IVs remains an open question in MR. On the one hand, exposure variance can be influenced by multiple SNPs that are in potential linkage disequilibrium (LD) with one another. On the other hand, incorporating more SNPs can increase the risk of bias in MR estimates caused by weak instrument bias or pleiotropy, as irrelevant SNPs may also be included in the analysis. To balance these considerations, our study applied multiple complementary MR methods with different IV selection strategies and pleiotropy assumptions.

MR methods can be categorized into one-sample MR and two-sample MR. In one-sample MR, individual-level data for the genotype, exposure, and outcome are obtained from the same cohort. The causal effect is typically estimated using two-stage least squares (2SLS) framework. In the first stage, the exposure is predicted from the IVs using the following linear model:

$$\boldsymbol{X}=\pi_{0}\boldsymbol{I}+\pi_{1}\boldsymbol{G}+\boldsymbol{\epsilon}_{1}$$

where $\boldsymbol{G}$ is the genotype matrix for instrumental SNPs, $\boldsymbol{X}$ is the observation vector for the exposure (assuming a single exposure for simplicity), $\pi_{1}$ is the regression coefficient, $\pi_{0}$ is the intercept, and $\boldsymbol{\epsilon}_{1}$ is the error term. In the second stage, the outcome is regressed on the predicted exposure:

$$\boldsymbol{Y}=\beta_{0}\boldsymbol{I}+\beta_{1}\hat{\boldsymbol{X}}+\boldsymbol{\epsilon}_{2}$$

where $\hat{\boldsymbol{X}}$ is the genetically predicted exposure from the first stage, $\boldsymbol{Y}$ denotes the observation vector for the outcome, $\beta_{1}$ is the causal effect, $\beta_{0}$ is the intercept, and $\boldsymbol{\epsilon}_{2}$ is the error term.


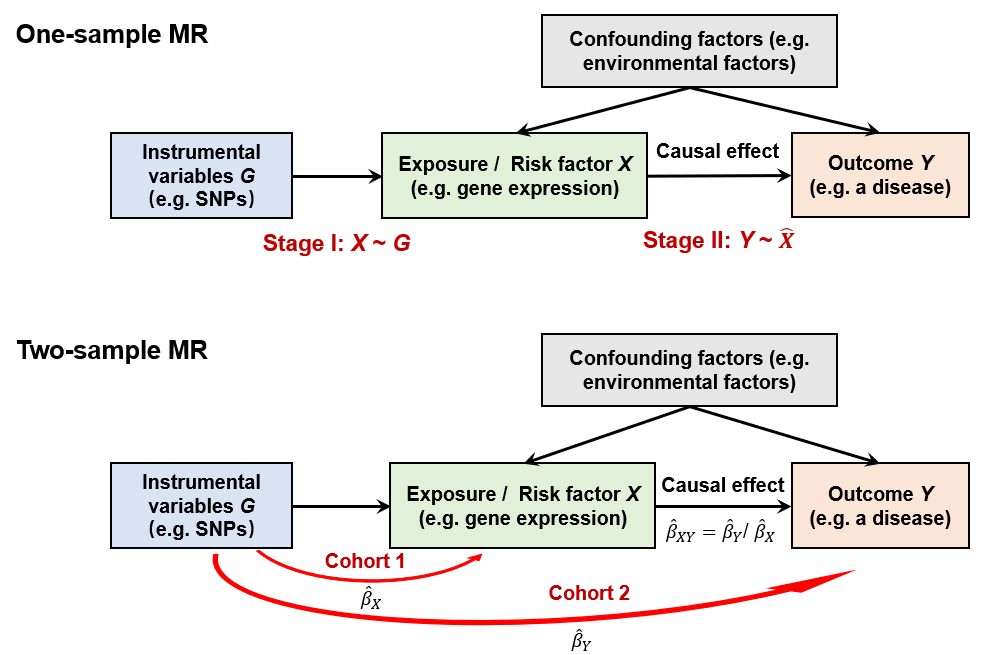


**Schematic of Mendelian Randomization.**

Two-sample MR uses GWAS summary statistics from two independent datasets: one for the exposure and one for the outcome. This enables causal inference in the absence of individual-level data. The most basic estimator in two-sample MR is the inverse-variance weighted (IVW) method^6-8^. Suppose $k$ is the number of independent SNPs selected as IVs, and $\hat{\beta}_{X_{i}}$ and $\hat{\beta}_{Y_{i}}$ denote the GWAS estimate of SNP $i$ on the exposure and the outcome, respectively. The causal effect estimate for SNP $i$ is then given by the Wald ratio:

$$\hat{\beta}_{{XY}_{i}}=\frac{\hat{\beta}_{Y_{i}}}{\hat{\beta}_{X_{i}}}$$

Its standard error can be approximated as^9^:

$$\mathrm{se}\left( \hat{\beta}_{{XY}_{i}} \right)\approx\frac{\mathrm{se}\left( \hat{\beta}_{Y_{i}} \right)}{\hat{\beta}_{X_{i}}}$$

The IVW estimator then combines the SNP-specific causal effects using inverse-variance weighting:

$$\hat{\beta}^{\mathrm{IVW}}=\frac{\sum_{i=1}^{k} \frac{\hat{\beta}_{{XY}_{i}}}{\mathrm{Var}\left( \hat{\beta}_{{XY}_{i}} \right)}}{\sum_{i=1}^{k} \frac{1}{\mathrm{Var}\left( \hat{\beta}_{{XY}_{i}} \right)}}=\frac{\sum_{i=1}^{k} \frac{{\hat{\beta}_{X_{i}}\hat{\beta}}_{Y_{i}}}{\mathrm{Var}\left( \hat{\beta}_{Y_{i}} \right)}}{\sum_{i=1}^{k} \frac{\hat{\beta}_{X_{i}}^{2}}{\mathrm{Var}\left( \hat{\beta}_{Y_{i}} \right)}}$$

It has been proven that 2SLS and IVW are asymptotically equivalent under ideal conditions (two samples are ancestry-matched, mutually independent, and of sufficiently large sample size)^10^. So two-sample MR can be viewed as an practical approximation to one-sample MR.

## Causal inference framework in this study

In this study, we aimed to identify causal relationships between cell type–specific gene expression and a broad range of brain-associated complex phenotypes. Because individual-level data were unavailable, we adopted a two-sample MR framework based on publicly available GWAS and eQTL summary statistics. In this context, *cis*-eQTLs measured in eight major brain cell types serve as IVs, gene expression serves as the exposure, and brain imaging-derived phenotypes (IDPs) and brain disorders or behaviors (DBs) serve as the outcomes.

The intuition is straightforward: *cis*-eQTLs provide exogenous variation in gene expression that is unlikely to be influenced by environmental or behavioral confounders. If a *cis*-eQTL-driven change in expression consistently predicts variation in a phenotype (IDP or DB) across multiple MR methods, it supports a putative causal effect of gene expression on the phenotype. Building on this idea, we further characterized putative causal routes among eGenes, IDPs, and DBs by integrating significant associations across eGene–IDP, eGene–DB, and IDP–DB pairs. These routes provide hypotheses on how gene regulation in specific brain cell types may influence brain structure and thereby affect disease risk, or how gene regulation may influence disease-related processes and in turn alter brain structure. We caution that these routes should not be interpreted as definitive causal pathways. They highlight possible regulatory mechanisms underlying brain-associated complex phenotypes and require further experimental validation.

# References

1 Sanderson, E. *et al.* Mendelian randomization. *Nat Rev Methods Primers* **2**, doi:10.1038/s43586-021-00092-5 (2022).

2 Bowden, J., Davey Smith, G. & Burgess, S. Mendelian randomization with invalid instruments: effect estimation and bias detection through Egger regression. *International journal of epidemiology* **44**, 512-525, doi:10.1093/ije/dyv080 (2015).

3 Burgess, S. *et al.* Guidelines for performing Mendelian randomization investigations: update for summer 2023. *Wellcome Open Res* **4**, 186, doi:10.12688/wellcomeopenres.15555.3 (2019).

4 Verbanck, M., Chen, C. Y., Neale, B. & Do, R. Detection of widespread horizontal pleiotropy in causal relationships inferred from Mendelian randomization between complex traits and diseases. *Nat Genet* **50**, 693-698, doi:10.1038/s41588-018-0099-7 (2018).

5 Sanderson, E., Davey Smith, G., Windmeijer, F. & Bowden, J. An examination of multivariable Mendelian randomization in the single-sample and two-sample summary data settings. *International journal of epidemiology* **48**, 713-727, doi:10.1093/ije/dyy262 (2019).

6 Burgess, S., Butterworth, A. & Thompson, S. G. Mendelian randomization analysis with multiple genetic variants using summarized data. *Genetic epidemiology* **37**, 658-665 (2013).

7 Burgess, S. & Thompson, S. G. Multivariable Mendelian randomization: the use of pleiotropic genetic variants to estimate causal effects. *American journal of epidemiology* **181**, 251-260, doi:10.1093/aje/kwu283 (2015).

8 Burgess, S., Dudbridge, F. & Thompson, S. G. Combining information on multiple instrumental variables in Mendelian randomization: comparison of allele score and summarized data methods. *Statistics in medicine* **35**, 1880-1906, doi:10.1002/sim.6835 (2016).

9 Thomas, D. C., Lawlor, D. A. & Thompson, J. R. Re: Estimation of bias in nongenetic observational studies using" Mendelian triangulation" by Bautista et al. (2007).

10 Burgess, S., Zuber, V., Valdes-Marquez, E., Sun, B. B. & Hopewell, J. C. Mendelian randomization with fine-mapped genetic data: Choosing from large numbers of correlated instrumental variables. *Genet Epidemiol* **41**, 714-725, doi:10.1002/gepi.22077 (2017).
