# Supplementary figures and images for: Deciphering cell type-specific causal genetic effects on brain imaging-derived phenotypes and disorders with single-cell Mendelian randomization
Source: PLoS Comput Biol. 2026 Jun 17;22(6):e1014422. doi: 10.1371/journal.pcbi.1014422 (PMC13289931; doi:10.1371/journal.pcbi.1014422)

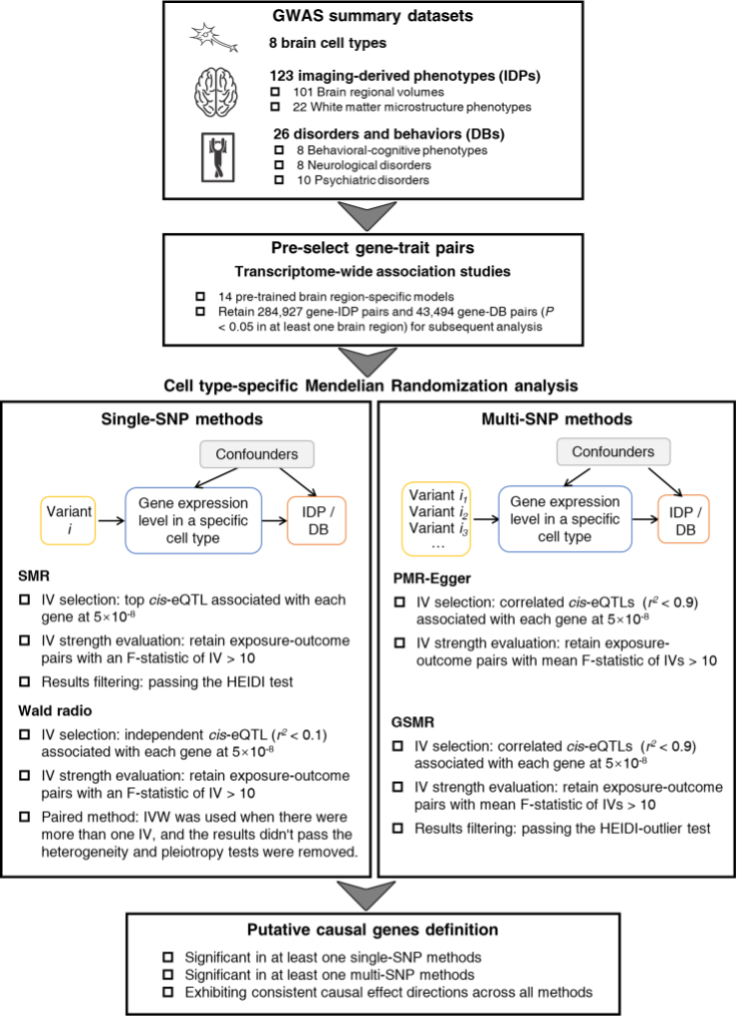

Supplement: S1 Fig — IV: instrumental variables. (TIF) [file pcbi.1014422.s004.tif]

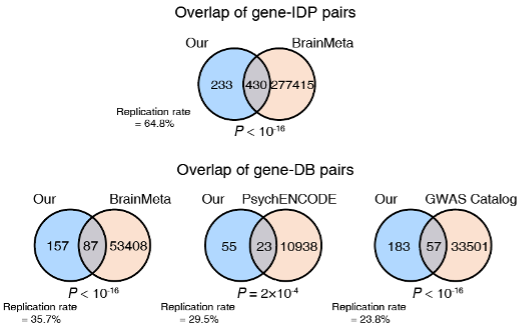

Supplement: S2 Fig — Reproducibility of eGene–IDP pairs was assessed using cortical cis-eQTL summary statistics from BrainMeta [36]. Reproducibility of eGene–DB pairs was assessed using cortical cis-eQTLs from BrainMeta [36], differentially expressed genes from PsychENCODE [29], and eGene–phenotype associations from the GWAS Catalog [1]. Significance of overlap was evaluated by hypergeometric test. (TIF) [file pcbi.1014422.s005.tif]

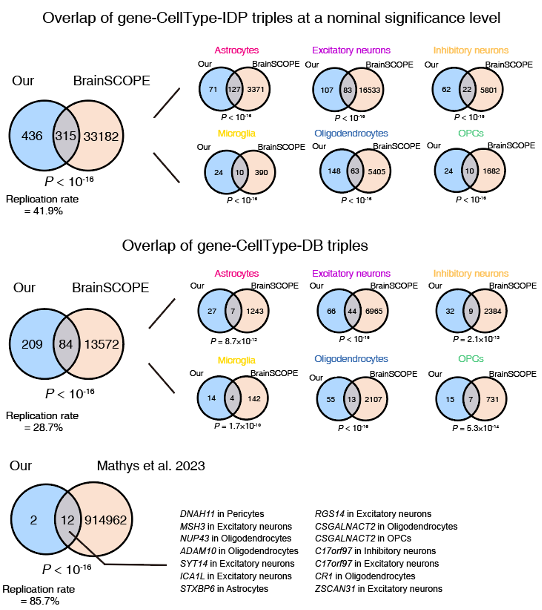

Supplement: S3 Fig — Reproducibility of eGene–cell type–IDP triples was assessed using cell type–specific eQTL summary statistics from brainSCOPE [37]. Reproducibility of eGene–cell type–DB triples was assessed using cell type–specific eQTLs from brainSCOPE [37] and differentially expressed genes from Mathys et al. [45] (only AD-associated causal eGenes were tested). Significance of overlap was evaluated by hypergeometric test. (TIF) [file pcbi.1014422.s006.tif]

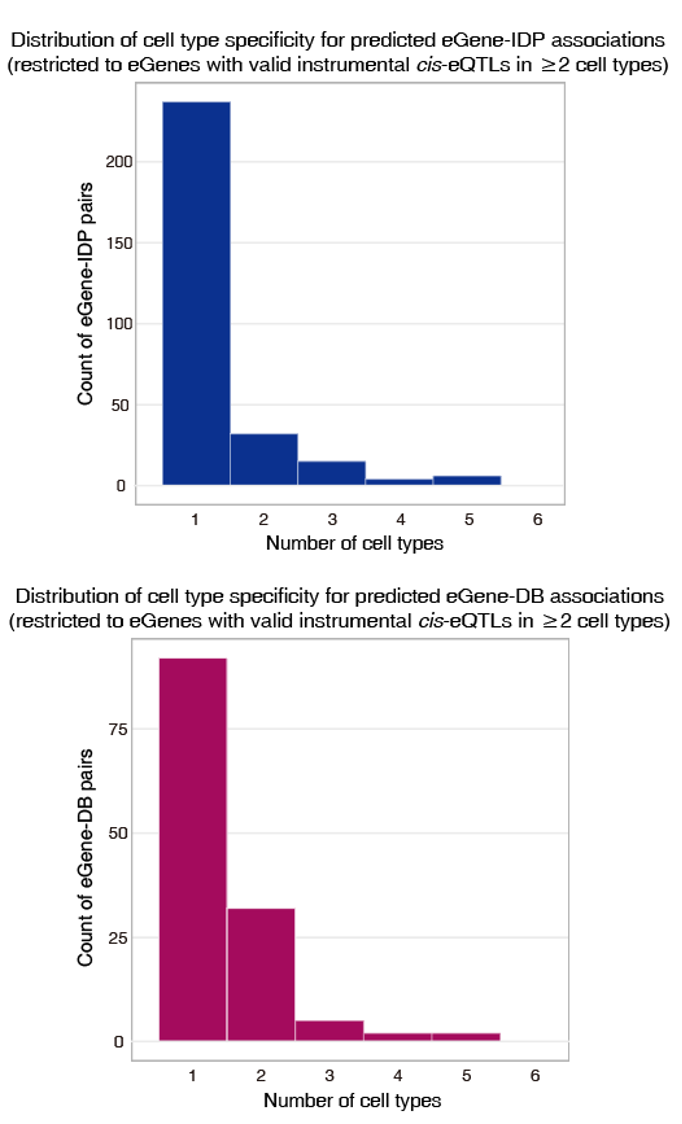

Supplement: S4 Fig — Bar plots show the number of eGene–phenotype associations predicted in exactly k cell types (x-axis), restricted to eGenes with valid instrumental cis-eQTLs in two or more cell types. The y-axis indicates the number of predicted eGene–phenotype associations detected in exactly k cell types. (TIF) [file pcbi.1014422.s007.tif]

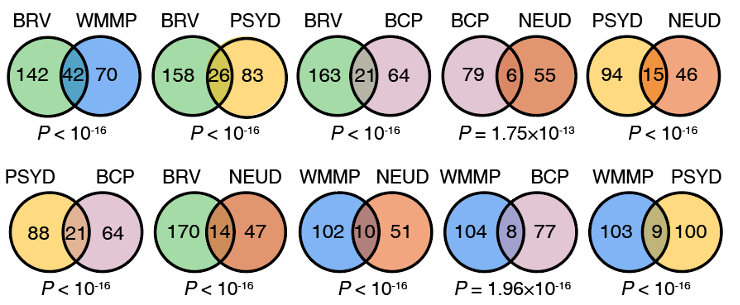

Supplement: S5 Fig — Overlaps were assessed by hypergeometric test. BRV: brain regional volume; WMMP: white matter microstructure phenotype; BCP: behavioral–cognitive phenotype; NEUD: neurological disorder; PSYD: psychiatric disorder. (TIF) [file pcbi.1014422.s008.tif]

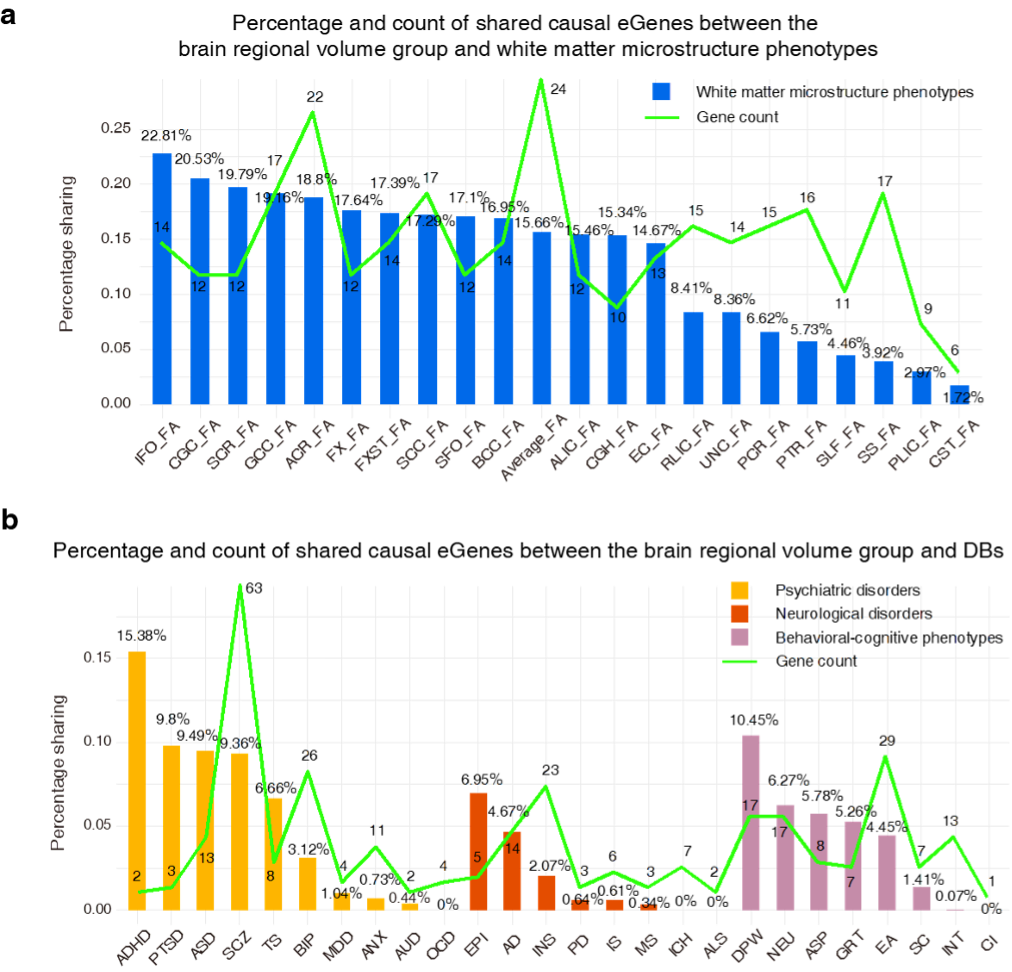

Supplement: S6 Fig — (a) Percentage and count of shared cell type–specific causal eGenes between the brain regional volume group and white matter microstructure phenotypes. (b) Percentage and count of shared cell type–specific causal eGenes between the brain regional volume group and DBs. (TIF) [file pcbi.1014422.s009.tif]

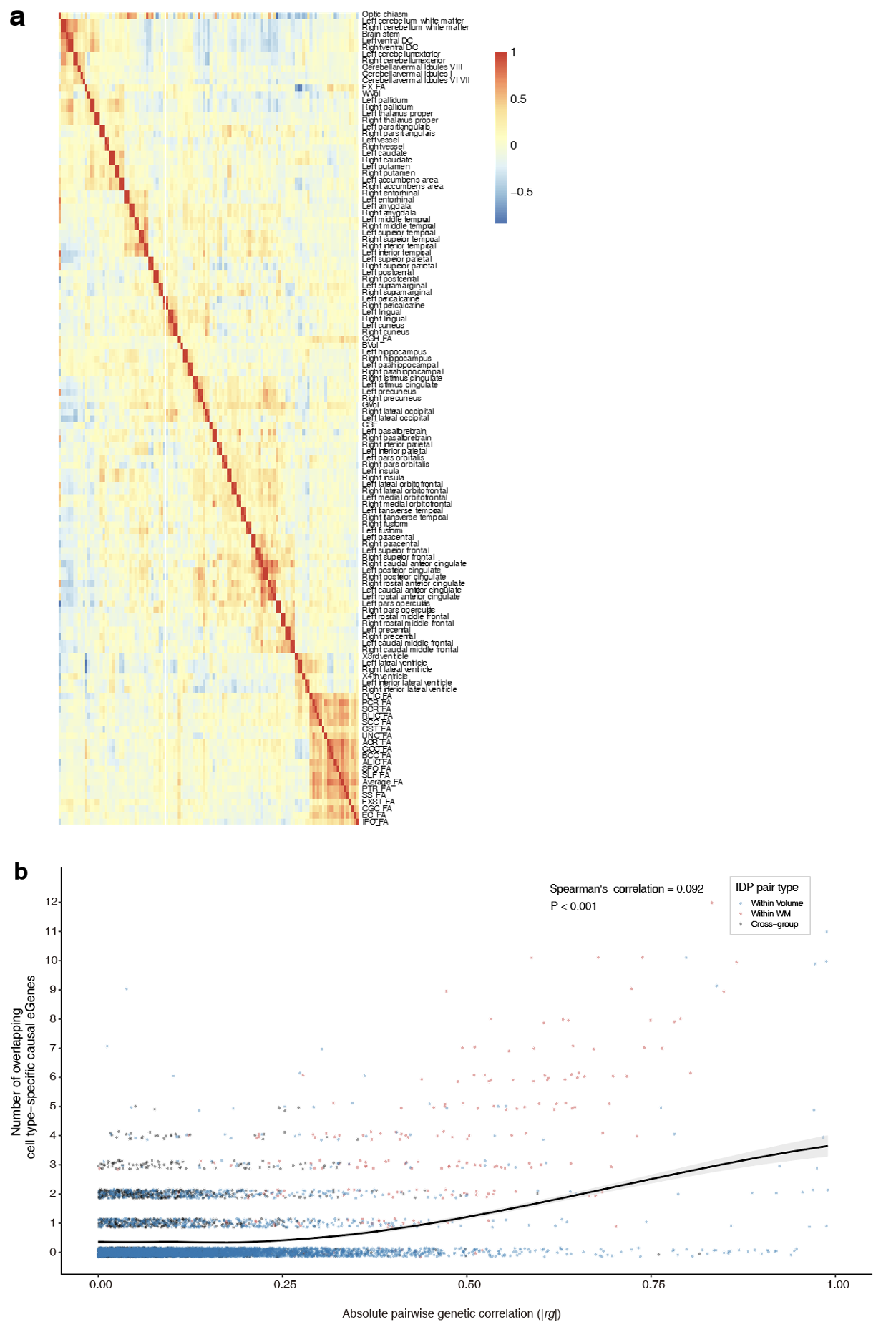

Supplement: S7 Fig — (a) Pairwise genetic correlations (rg) were estimated for all 123 IDPs using LD Score Regression based on GWAS summary statistics [167]. Correlations range from −0.83 to 1. The strongest correlation between IDPs from different groups was observed between the fractional anisotropy of the posterior limb of the internal capsule (PLIC_FA) and the volume of the right lateral ventricle (rg = 0.49). (b) Relationship between pairwise genetic correlation and overlap of cell type–specific causal eGenes among IDPs. The x-axis shows absolute pairwise genetic correlation (|rg|), and the y-axis shows the number of overlapping cell type–specific causal eGenes identified by MR analysis. Each point represents one IDP pair, colored by pair type: between-group (one brain regional volume and one white matter microstructure phenotype), within-BRV (both brain regional volumes), or within-WMMP (both white matter microstructure phenotypes). The black curve shows a LOESS-smoothed trend, with the shaded area indicating the 95% confidence interval. (TIF) [file pcbi.1014422.s010.tif]

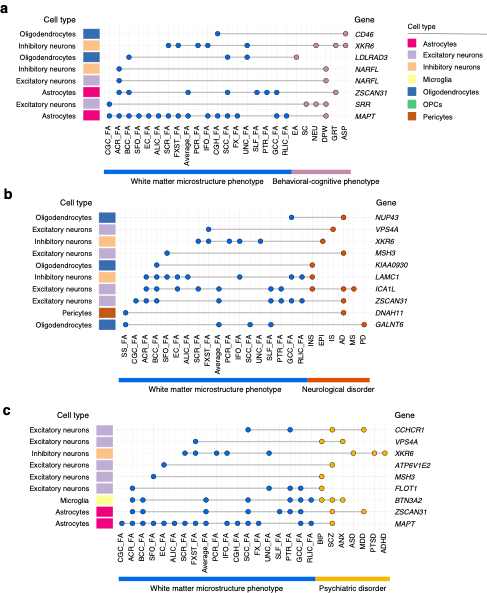

Supplement: S8 Fig — (TIF) [file pcbi.1014422.s011.tif]

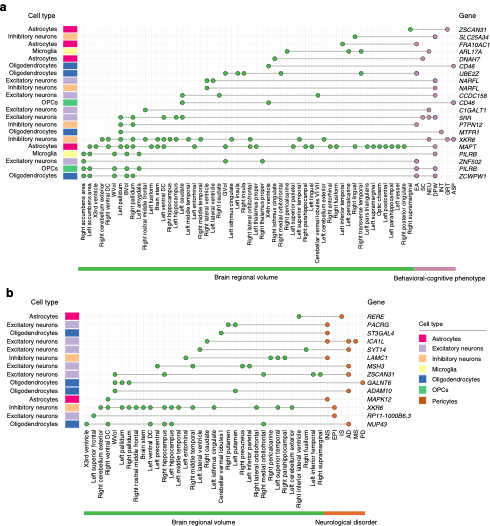

Supplement: S9 Fig — (TIF) [file pcbi.1014422.s012.tif]

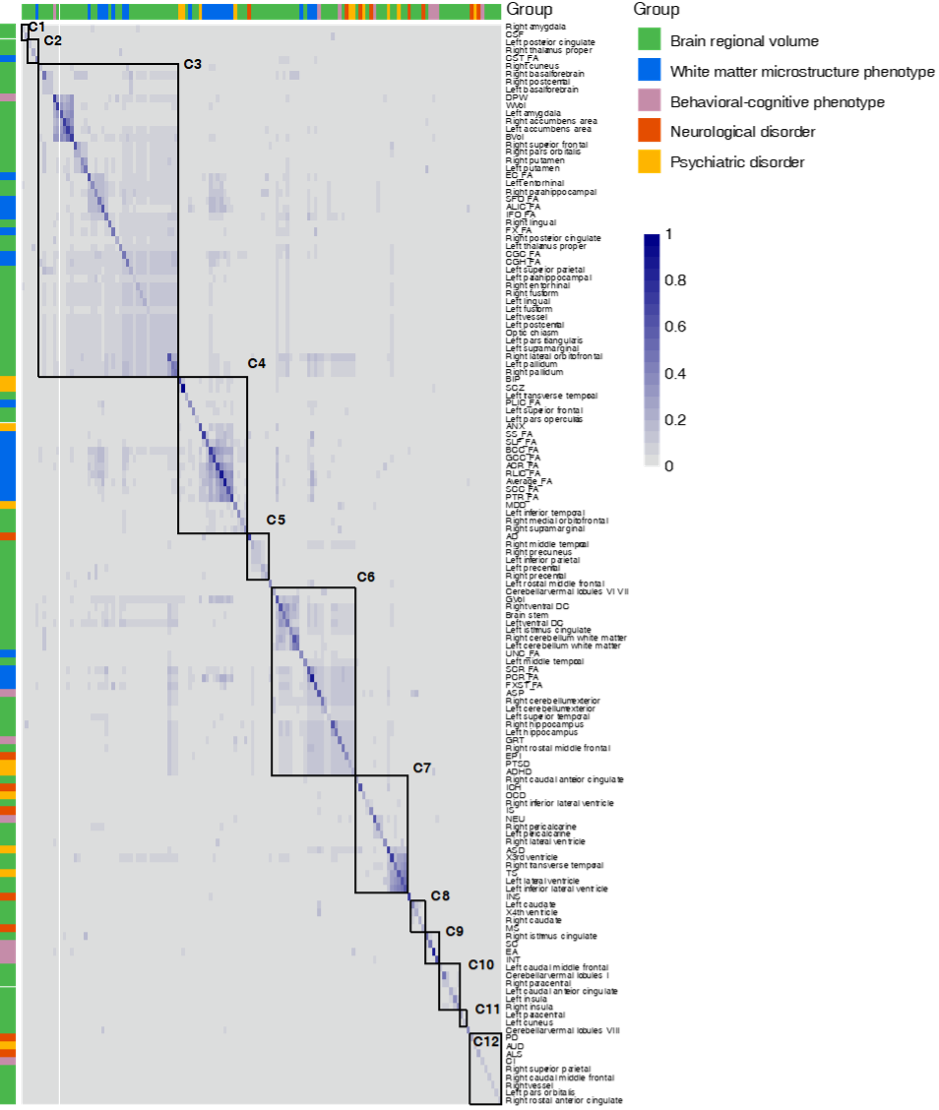

Supplement: S10 Fig — For each cell type, we constructed a phenotype × phenotype Jaccard similarity matrix based on shared causal eGenes. These were averaged to generate a phenotype similarity matrix, followed by hierarchical clustering. The resulting 12 phenotype clusters (C1–C12) are annotated on the heatmap. (TIF) [file pcbi.1014422.s013.tif]

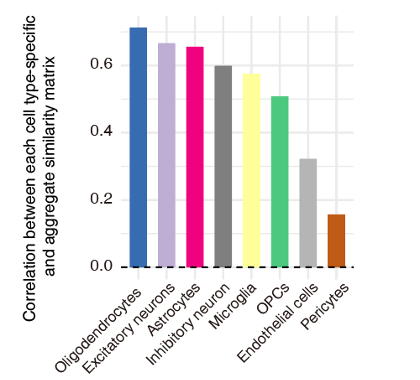

Supplement: S11 Fig — Bar plot showing the Pearson correlation between each cell type–specific phenotype similarity matrix and the phenotype similarity matrix. Higher correlations indicate greater contributions of the corresponding cell type to the global phenotype similarity pattern. (TIF) [file pcbi.1014422.s014.tif]

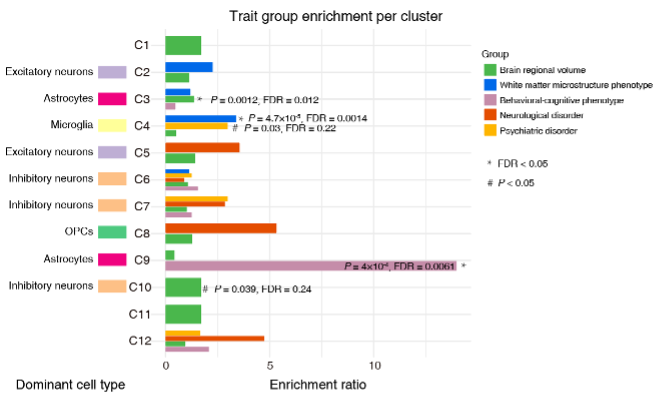

Supplement: S12 Fig — Bar plot showing the enrichment ratio of each phenotype group within the 12 identified phenotype clusters. Significance level is indicated (* FDR < 0.05, # P < 0.05; hypergeometric test). The dominant cell type contributing to each cluster’s similarity structure (defined as having the highest correlation with the phenotype similarity matrix within that cluster) is shown on the left. (TIF) [file pcbi.1014422.s015.tif]

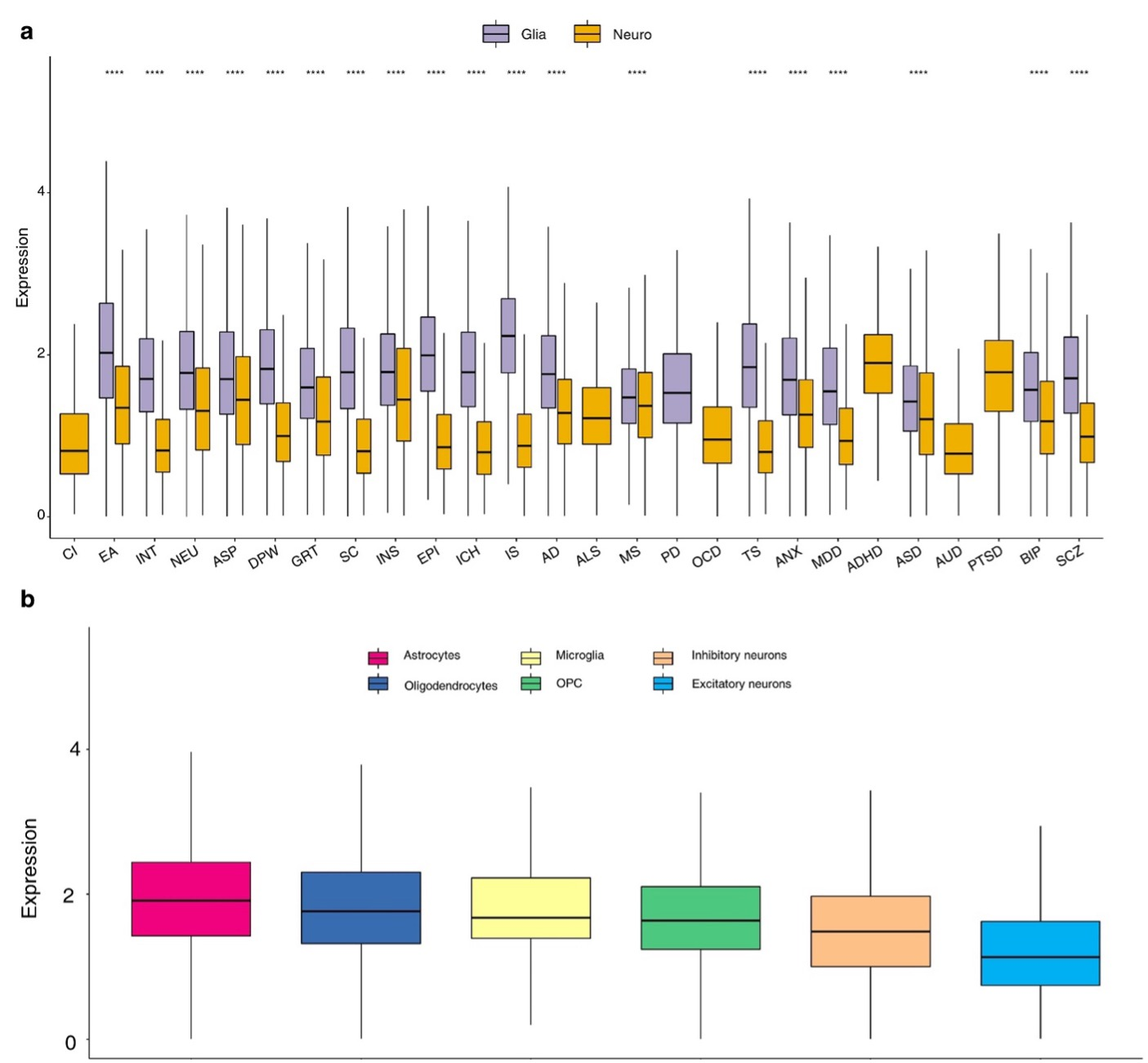

Supplement: S13 Fig — (a) Expression differences in glial and neural cell types for various DBs, with significance indicated as ***, P < 0.001. (b) Detailed expression profiles of causal eGenes for 26 DBs in glial and neural cell types. Glia: glial cells, including astrocytes, microglia, oligodendrocytes, and OPCs. Neuro: neuronal cells, including inhibitory and excitatory neurons. (TIF) [file pcbi.1014422.s016.tif]

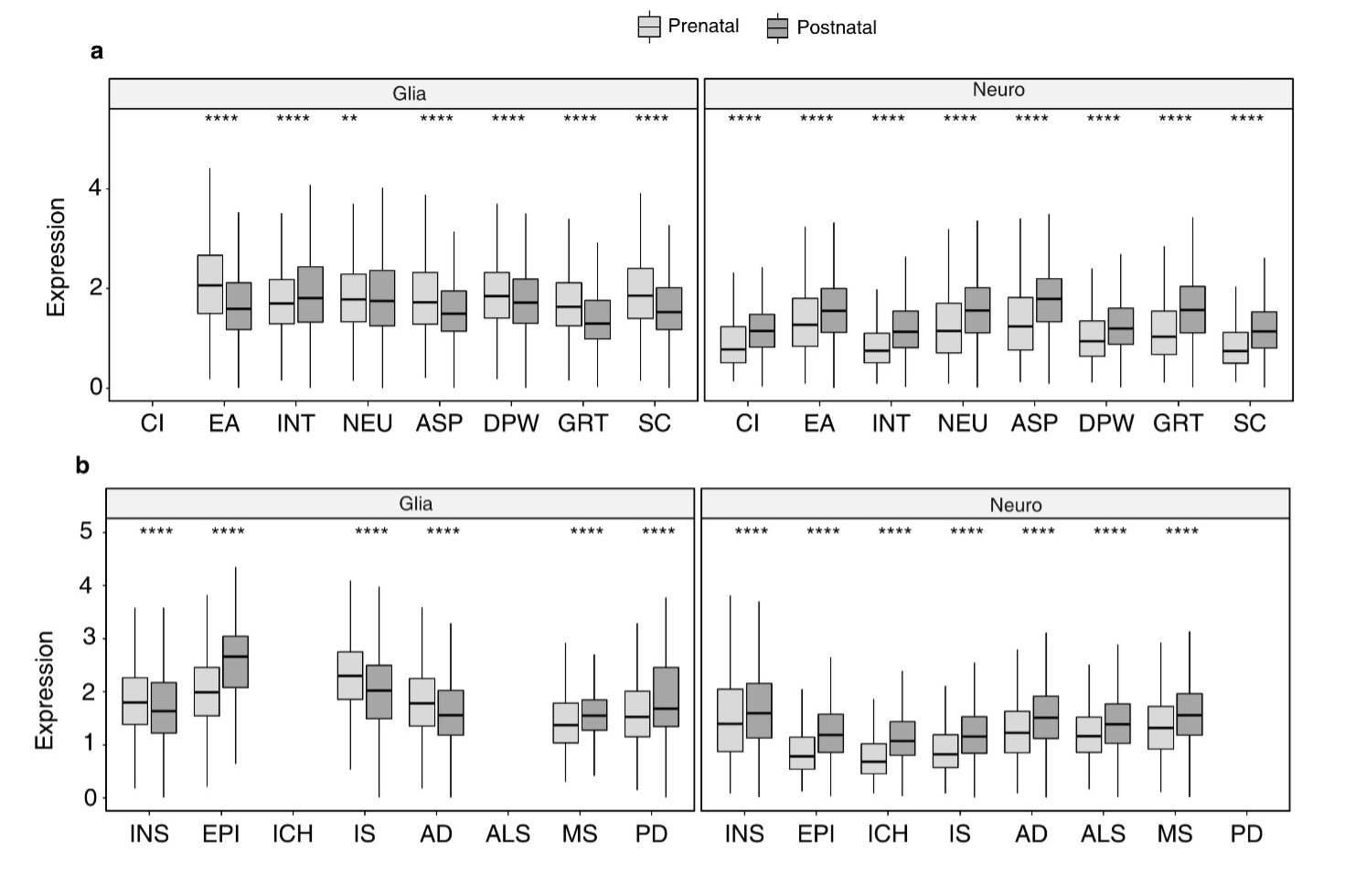

Supplement: S14 Fig — Glia: glial cells, including astrocytes, microglia, oligodendrocytes, and OPCs. Neuro: neuronal cells, including inhibitory and excitatory neurons. * P < 0.05; ** P < 0.01; **** P < 0.001. (TIF) [file pcbi.1014422.s017.tif]

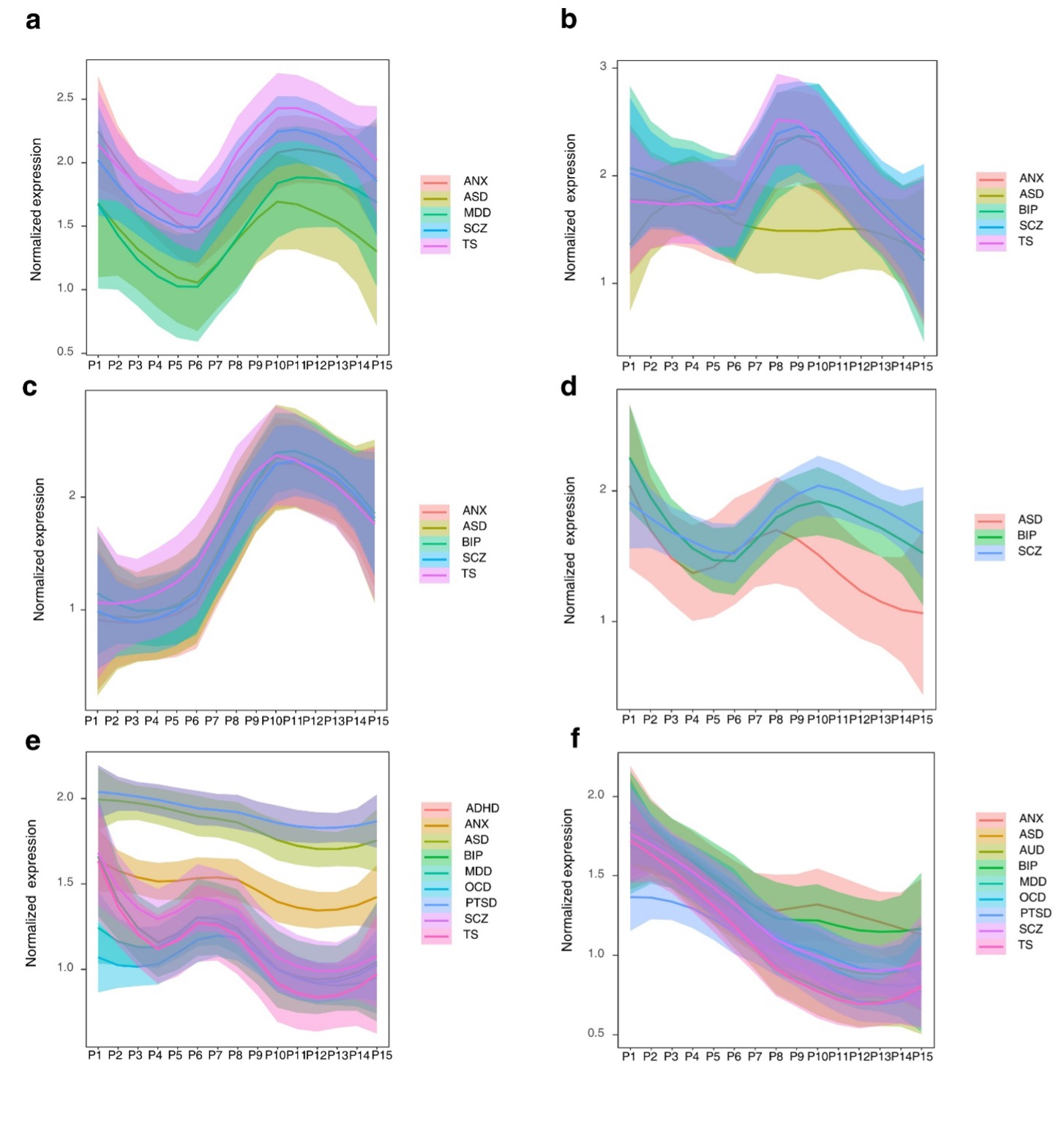

Supplement: S15 Fig — Locally estimated scatterplot smoothing (LOESS) curves are shown with 95% confidence intervals. P1, 4 ≤ age < 8 PCW; P2, 8 ≤ age < 10 PCW; P3, 10 ≤ age < 13 PCW; P4, 13 ≤ age < 16 PCW; P5, 16 ≤ age < 19 PCW; P6, 19 ≤ age < 24 PCW; P7, 24 ≤ age < 38 PCW; P8, 0 ≤ age < 6 months; P9, 6 ≤ age < 12 months; P10, 1 ≤ age < 6 years; P11, 6 ≤ age < 12 years; P12, 12 ≤ age < 20 years; P13, 20 ≤ age < 40 years; P14, 40 ≤ age < 60 years; P15, > 60 years. (TIF) [file pcbi.1014422.s018.tif]

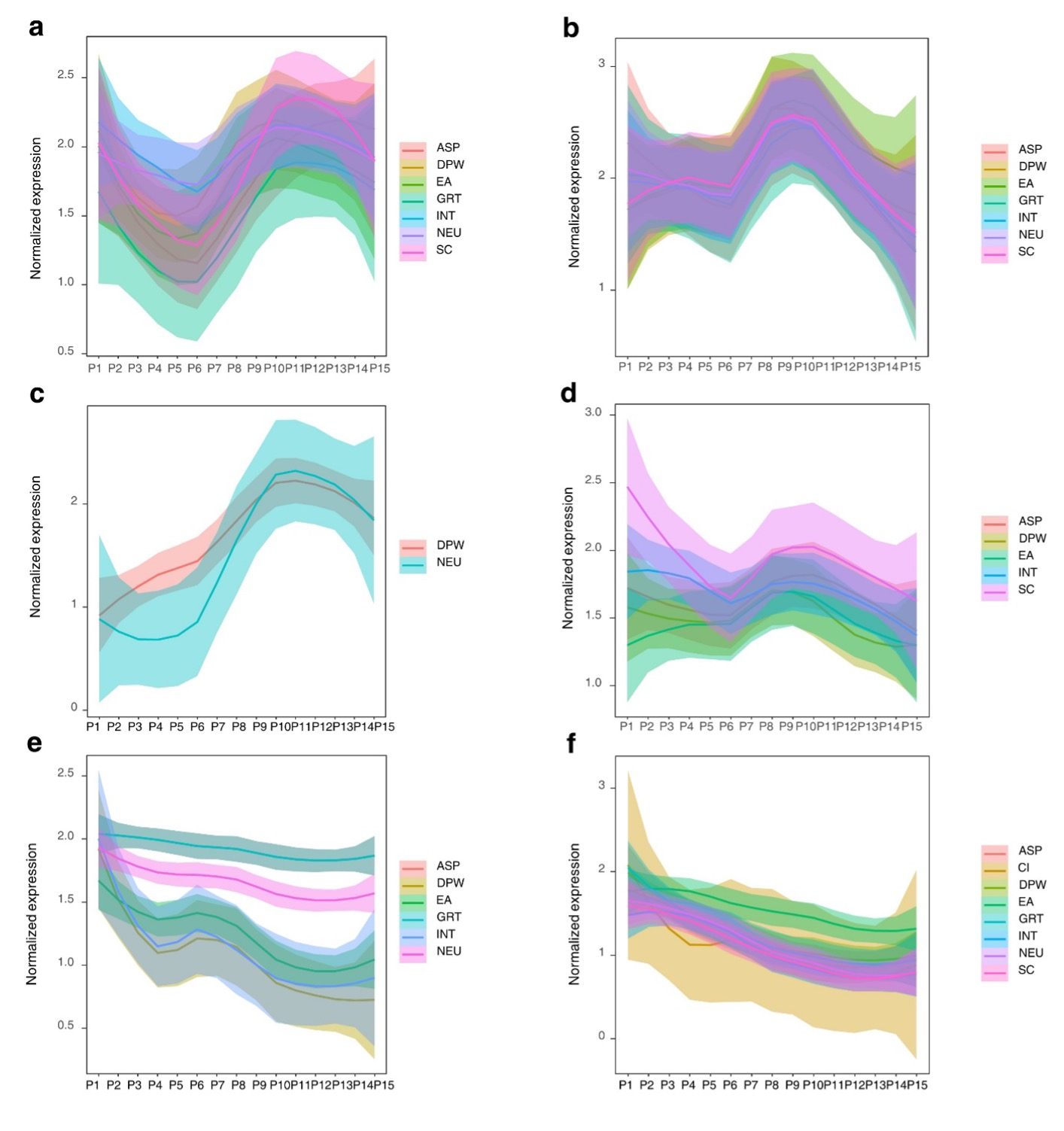

Supplement: S16 Fig — For the locally estimated scatterplot smoothing (LOESS) plots, smooth curves are shown with 95% confidence intervals. P1, 4 ≤ Age < 8 PCW; P2, 8 ≤ Age < 10 PCW; P3, 10 ≤ Age < 13 PCW; P4, 13 ≤ Age < 16 PCW; P5, 16 ≤ Age < 19 PCW; P6, 19 ≤ Age < 24 PCW; P7, 24 ≤ Age < 38 PCW; P8, 0 ≤ Age < 6 Months; P9, 6 ≤ Age < 12 Months; P10, 1 ≤ Age < 6 Years; P11, 6 ≤ Age < 12 Years; P12, 12 ≤ Age < 20 Years; P13, 20 ≤ Age < 40 Years; P14, 40 ≤ Age < 60 Years; P15, > 60 Years. (TIF) [file pcbi.1014422.s019.tif]

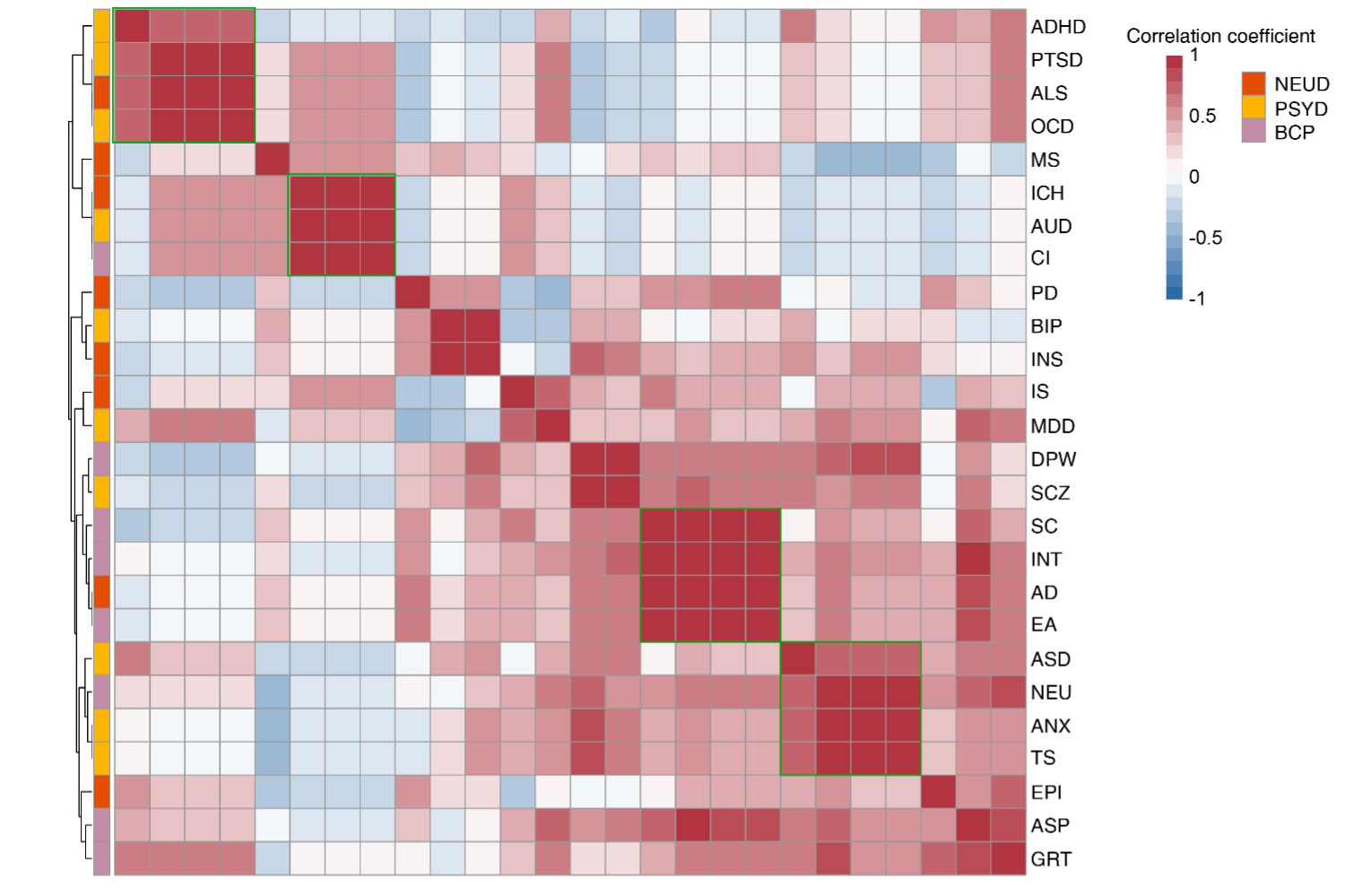

Supplement: S17 Fig — Associations were evaluated based on expression correlation (Spearman’s rank correlation coefficient) of causal eGenes across eight cell types (Methods), and clusters were derived using hierarchical clustering (complete linkage). (TIF) [file pcbi.1014422.s020.tif]

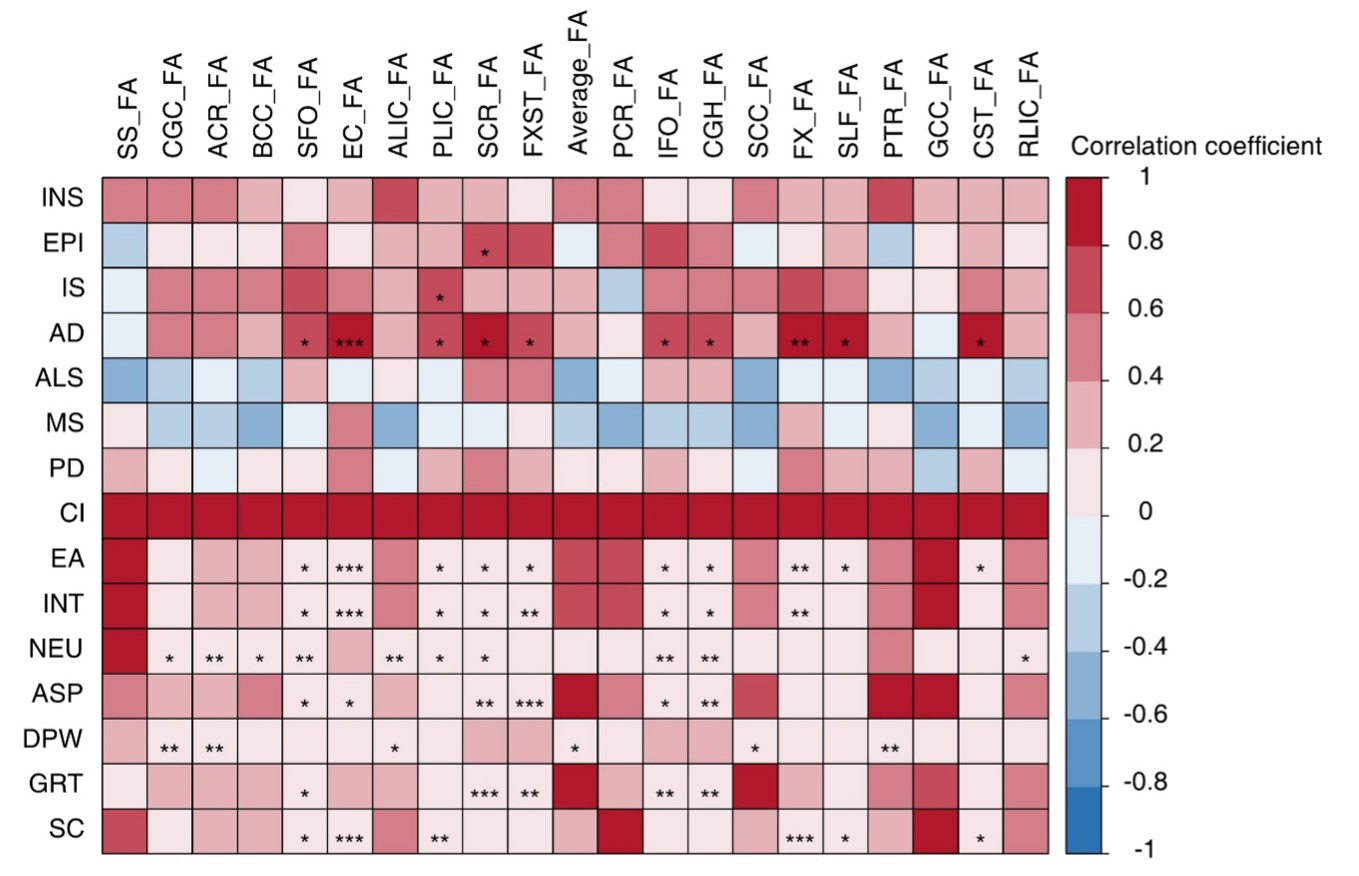

Supplement: S18 Fig — Associations were evaluated based on expression correlation (Spearman’s rank correlation coefficient) of causal eGenes across eight cell types (Methods). * FDR < 0.05; ** FDR < 0.01; *** FDR < 0.001. (TIF) [file pcbi.1014422.s021.tif]

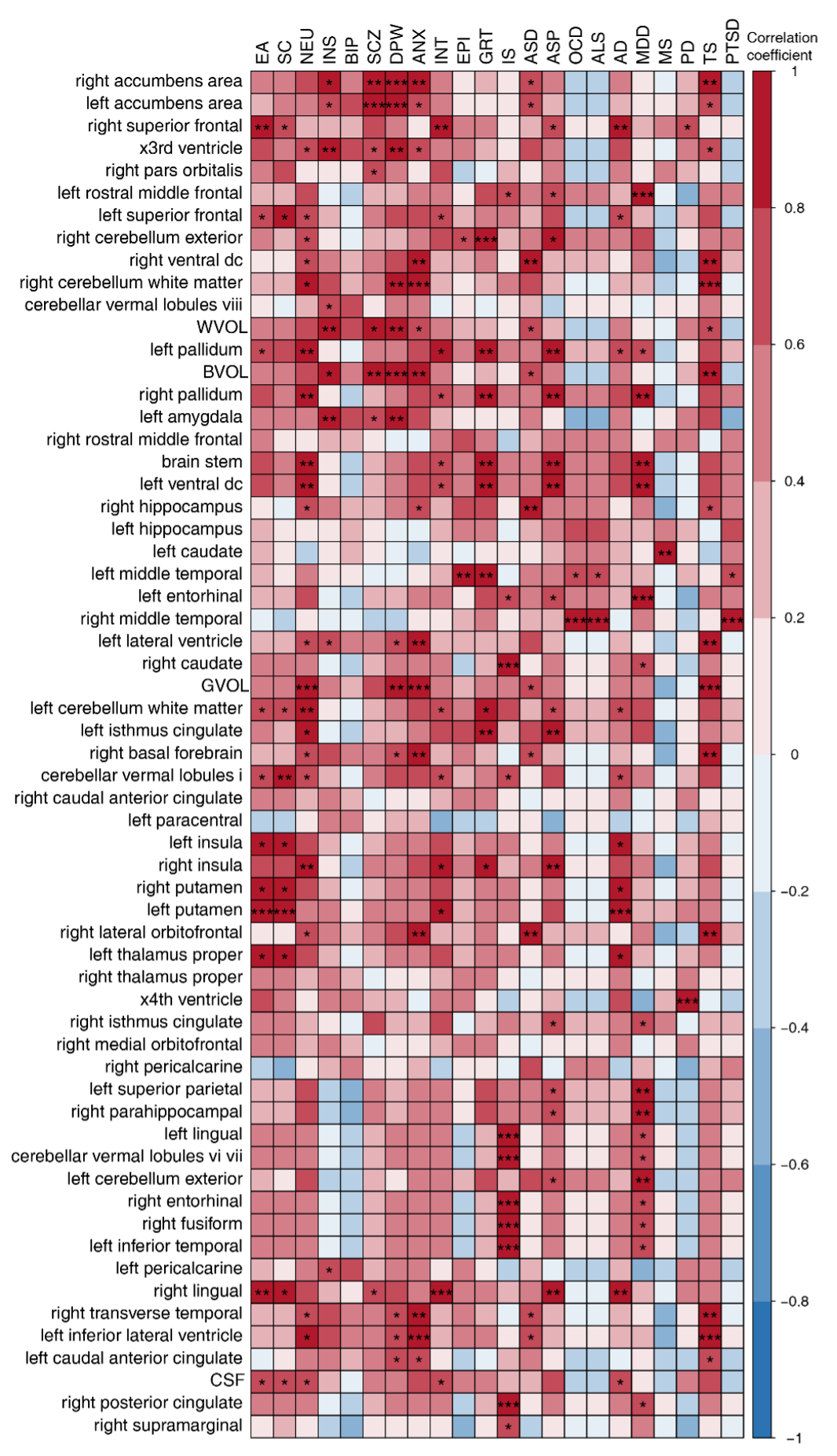

Supplement: S19 Fig — Associations were evaluated based on expression correlation (Spearman’s rank correlation coefficient) of causal eGenes across eight cell types (Methods). * FDR < 0.05; ** FDR < 0.01; *** FDR < 0.001. (TIF) [file pcbi.1014422.s022.tif]
