# Supplementary material for: Deciphering cell type-specific causal genetic effects on brain imaging-derived phenotypes and disorders with single-cell Mendelian randomization
Source: PLoS Comput Biol. 2026 Jun 17;22(6):e1014422. doi: 10.1371/journal.pcbi.1014422 (PMC13289931; doi:10.1371/journal.pcbi.1014422)
Supplement: S1 STROBE Checklist — (DOCX) [file pcbi.1014422.s036.docx]

**S14 Table. STROBE-MR checklist of recommended items to address in reports of Mendelian randomization studies**^1^ ^2^

| **Item No.** | **Section** | **Checklist item** | **Page No.** | **Relevant text from manuscript** |
| --- | --- | --- | --- | --- |
| 1 | **TITLE and ABSTRACT** | Indicate Mendelian randomization (MR) as the study’s design in the title and/or the abstract if that is a main purpose of the study | 1, 2 | Title: Deciphering cell type-specific causal genetic effects on brain imaging-derived phenotypes and disorders with single-cell Mendelian randomization  Abstract: Here, we develop a single-cell Mendelian randomization framework to infer cell type-specific causal relationships between gene expression and diverse brain-associated complex phenotypes by integrating single-cell expression quantitative trait loci (*cis*-eQTLs) and genome-wide association study findings. |
|  | **INTRODUCTION** |  |  |  |
| 2 | **Background** | Explain the scientific background and rationale for the reported study. What is the exposure? Is a potential causal relationship between exposure and outcome plausible? Justify why MR is a helpful method to address the study question | 4 | By integrating *cis*-eQTL data with GWAS findings, Mendelian randomization (MR)—a framework for genetic causal inference—can pinpoint genes whose expression causally influences complex neurological, psychological, and behavioral phenotypes [6-9]. |
| 3 | **Objectives** | State specific objectives clearly, including pre-specified causal hypotheses (if any). State that MR is a method that, under specific assumptions, intends to estimate causal effects | 4 | By integrating *cis*-eQTL data with GWAS findings, Mendelian randomization (MR)—a framework for genetic causal inference—can pinpoint genes whose expression causally influences complex neurological, psychological, and behavioral phenotypes [6-9].  *Note: This study uses several complementary MR methods to infer the causal relationship. We specify the corresponding hypotheses in METHODS section.* |
|  | **METHODS** |  |  |  |
| 4 | **Study design and data sources** | Present key elements of the study design early in the article. Consider including a table listing sources of data for all phases of the study. For each data source contributing to the analysis, describe the following: |  |  |
|  | a) | Setting: Describe the study design and the underlying population, if possible. Describe the setting, locations, and relevant dates, including periods of recruitment, exposure, follow-up, and data collection, when available. | 27-29 | All individuals fell within three standard deviations of the mean for the first and second principal components of European-ancestry populations from the 1000 Genomes Project [12].  Most were published recently and derived from meta-analyses with large sample sizes (mean ~280,000 individuals). All GWAS summary statistics were of European ancestry, and there was no participant overlap with the cell type–specific *cis*-eQTL dataset.  The brain volume GWAS summary statistics were processed by Zhao et al. [147] from 19,629 UK Biobank participants of European ancestry (reported mean heritability ~40%).  The white matter tract GWAS summary statistics were processed by Zhao et al. [22] from 33,292 UK Biobank participants of European ancestry (reported mean heritability ~46.3%). |
|  | b) | Participants: Give the eligibility criteria, and the sources and methods of selection of participants. Report the sample size, and whether any power or sample size calculations were carried out prior to the main analysis | 27-29 | Notably, the *cis*-eQTLs from brain cell types were identified by integrating high-quality single-nuclei RNA sequencing data from the prefrontal cortex, temporal cortex, and white matter, and corresponding genotype data from 192 individuals.  Most were published recently and derived from meta-analyses with large sample sizes (mean ~280,000 individuals). All GWAS summary statistics were of European ancestry, and there was no participant overlap with the cell type–specific *cis*-eQTL dataset.  The GWAS summary statistics for brain volume phenotypes were processed by Zhao et al. [158], with a sample size of 19,629 participants of European ancestry from UKB (reported mean heritability: ~40%).  The GWAS summary statistics for white matter tracts were processed by Zhao et al. [22], with a sample size of 33,292 participants of European ancestry from UKB (reported mean heritability: ~46.3%). |
|  | c) | Describe measurement, quality control and selection of genetic variants | 30 | The top cis-eQTL associated with each gene expression in a given cell type at genome-wide significance (*P* < 5×10^-8^) was selected as the instrument.  For instrument selection, LD clumping (*r^2^* < 0.1, window = 250 kb, 1000 Genomes EUR reference panel) was first performed for *cis*-eQTLs associated with each gene expression at genome-wide significance (*P* < 5×10^-8^) using PLINK v1.90 [154]. The independent *cis*-eQTL(s) for each gene in the given cell type were then selected as instrument(s).  The *cis*-eQTLs (clumping *r^2^* < 0.9, window = 250 kb, 1000 Genomes EUR reference panel) associated with each gene expression in a given cell type at genome-wide significance (*P* < 5×10^-8^) were selected as instruments. |
|  | d) | For each exposure, outcome, and other relevant variables, describe methods of assessment and diagnostic criteria for diseases | S1 Table, S2 Table, S3 Table | *Note: We used GWAS summary statistics to perform two-sample MR to investigate the causal effects of cell type-specific gene expression on 149 brain-associated complex traits. Summary of the dataset used in this research is in S1 Table, S2 Table and S3 Table.* |
|  | e) | Provide details of ethics committee approval and participant informed consent, if relevant | - | *Note: We only used publicly available GWAS summary statistics in this study. Therefore, there was no need to obtain ethics committee approval or informed consent from participants.* |
| 5 | **Assumptions** | Explicitly state the three core IV assumptions for the main analysis (relevance, independence and exclusion restriction) as well assumptions for any additional or sensitivity analysis | 30 | The GSMR method extends SMR to estimate the causal effect of an exposure on an outcome, leveraging multiple variants while accounting for LD among them [33]. It is applicable to any exposure under the standard MR assumptions. |
| 6 | **Statistical methods: main analysis** | Describe statistical methods and statistics used |  |  |
|  | a) | Describe how quantitative variables were handled in the analyses (i.e., scale, units, model) | - | *Note: In this study, we employed well-established Mendelian randomization methods, including SMR, Wald Ratio, PMR-Egger, and GSMR, to analyse the publicly available GWAS summary statistics. All variables were used in their original summary statistics form (e.g., effect sizes, standard errors, p-values) without any modifications or scaling. No further handling of individual quantitative variables was performed during the analysis.* |
|  | b) | Describe how genetic variants were handled in the analyses and, if applicable, how their weights were selected | 30 | The top cis-eQTL associated with each gene expression in a given cell type at genome-wide significance (*P* < 5×10^-8^) was selected as the instrument.  For instrument selection, LD clumping (*r^2^* < 0.1, window = 250 kb, 1000 Genomes EUR reference panel) was first performed for *cis*-eQTLs associated with each gene expression at genome-wide significance (*P* < 5×10^-8^) using PLINK v1.90 [154]. The independent *cis*-eQTL(s) for each gene in the given cell type were then selected as instrument(s).  The *cis*-eQTLs (clumping *r^2^* < 0.9, window = 250 kb, 1000 Genomes EUR reference panel) associated with each gene expression in a given cell type at genome-wide significance (*P* < 5×10^-8^) were selected as instruments. |
|  | c) | Describe the MR estimator (e.g. two-stage least squares, Wald ratio) and related statistics. Detail the included covariates and, in case of two-sample MR, whether the same covariate set was used for adjustment in the two samples | 30 | (2) The Wald ratio method computes the change in disease risk per standard deviation change in gene expression, using the instrumental *cis*-eQTL for the target gene. It is applicable to any exposure and requires only one SNP as the instrument under the standard MR assumptions (relevance, independence, and exclusion restriction). For instrument selection, LD clumping (*r^2^* < 0.1, window = 250 kb, 1000 Genomes EUR reference panel) was first performed for *cis*-eQTLs associated with each gene expression at genome-wide significance (*P* < 5×10^-8^) using PLINK v1.90 [154]. The independent cis-eQTL(s) for each gene in the given cell type were then selected as instrument(s). After instrument selection, 86.6% of cell type–specific genes had one valid instrumental *cis*-eQTL, so the Wald ratio could be applied in most analyses. For the remaining 13.4% of genes with more than one valid instrument, the inverse-variance weighted (IVW, fixed-effects) method was used as a supplementary approach [155], which is commonly paired with the Wald ratio in the literature [20, 156]. Both methods were implemented with the R package *TwoSampleMR* (version 0.5.10) [157]. The *harmonise_data()* function in TwoSampleMR was used to harmonize effect alleles and SNP effects between exposure and outcome. Heterogeneity (function *mr_heterogeneity*, P < 0.05) and pleiotropy (function *mr_pleiotropy_test*, *P* < 0.05) tests were conducted for IVW results. |
|  | d) | Explain how missing data were addressed | - | *Note: Only GWAS summary statistics were used in this study; no individual-level data were involved. As such, issues related to missing individual-level data do not apply. All summary statistics utilized were obtained from publicly available, quality-controlled GWAS datasets, in which missing data had already been addressed by the original studies.* |
|  | e) | If applicable, indicate how multiple testing was addressed | 31 | A gene was deemed putatively causal for a phenotype within a given cell type (cell type–specific causal eGene) if it met the following criteria: (1) significant in at least one single-SNP method after FDR correction (FDR < 0.05) [158]; (2) significant in at least one multi-SNP method after FDR correction (FDR < 0.05); (3) consistent causal effect direction across all four methods, reducing the likelihood of false positives. |
| 7 | **Assessment of assumptions** | Describe any methods or prior knowledge used to assess the assumptions or justify their validity | 31 | GSMR incorporates the HEIDI-outlier test to identify and exclude instruments with significant pleiotropic effects on the outcome (*P* < 0.05). Both GSMR and the HEIDI-outlier test were implemented with the R package gsmr (version 1.1.0).  We also calculated the F-statistic for each instrument using the Cragg-Donald statistic to assess instrument strength [159] by $F=R^{2}\times\frac{N-1-k}{(1-R^{2})\times k}$, where $R^{2}=\frac{\beta^{2}}{\beta^{2}+se^{2}\times N}$ represents the proportion of exposure variance explained by the instrument, *N* is the sample size from the *cis*-eQTL summary statistics, *β* is the effect size, se is the standard error, and *k* is the number of instruments used in the MR estimate (*k* = 1 for a single instrument). Genes for which the averaged F-statistic across all instruments exceeded 10 were retained.  |
| 8 | **Sensitivity analyses and additional analyses** | Describe any sensitivity analyses or additional analyses performed (e.g. comparison of effect estimates from different approaches, independent replication, bias analytic techniques, validation of instruments, simulations) | 31 | A gene was deemed putatively causal for a phenotype within a given cell type (cell type–specific causal eGene) if it met the following criteria: (1) significant in at least one single-SNP method after FDR correction (FDR < 0.05) [158]; (2) significant in at least one multi-SNP method after FDR correction (FDR < 0.05); (3) consistent causal effect direction across all four methods, reducing the likelihood of false positives. |
| 9 | **Software and pre-registration** |  |  |  |
|  | a) | Name statistical software and package(s), including version and settings used |  | SMR and the HEIDI test were implemented with SMR software (version 1.3.1, https://yanglab.westlake.edu.cn/software/smr).  Both methods were implemented with the R package TwoSampleMR (version 0.5.10) [157].  PMR-Egger was implemented with the R package PMR (version 1.0).  Both GSMR and the HEIDI-outlier test were implemented with the R package gsmr (version 1.1.0). |
|  | b) | State whether the study protocol and details were pre-registered (as well as when and where) | - | *Note: We did not pre-register the study protocol prior to conducting the analyses.* |
|  | **RESULTS** |  |  |  |
| 10 | **Descriptive data** |  |  |  |
|  | a) | Report the numbers of individuals at each stage of included studies and reasons for exclusion. Consider use of a flow diagram | 27-29 | The *cis*-eQTLs were identified by integrating high-quality single-nuclei RNA sequencing data from the prefrontal cortex, temporal cortex, and white matter with corresponding genotype data from 192 individuals.  The sample size for each GWAS summary statistics dataset is listed below.   - Behavioral-cognitive phenotypes   (1) Automobile speeding propensity (ASP): 404,291 individuals (heritability: 7.9%) [86]  (2) Childhood intelligence (CI): 12,441 individuals (heritability: 22%-46%) [124]  (3) Drinks per week (DPW): 941,280 individuals (heritability: 4%) [125]  (4) Educational attainment (EA): 766,345 individuals (heritability: 12.2%) [126]  (5) General risk tolerance (GRT): 466,571 individuals (heritability: 4.6%) [86]  (6) Intelligence (INT): 269,867 individuals (heritability: 19%) [127]  (7) Neuroticism (NEU): 390,278 individuals (heritability: 10%) [128]  (8) Smoking cessation (SC): 547,219 individuals (heritability: 5%) [125]   - Neurological disorders   (1) Alzheimer's disease (AD): 71,880 cases and 383,378 controls (heritability: 5.5%) [129]  (2) Amyotrophic lateral sclerosis (ALS): 20,806 cases and 59,804 controls (heritability: not reported in the original GWAS publication) [130]  (3) Epilepsy (EPI): 15,212 cases and 29,677 controls (heritability: 9.7%) [131]  (4) Intracerebral hemorrhage (ICH): 1,545 cases and 1,481 controls (heritability: not reported in the original GWAS publication) [132]  (5) Insomnia (INS): 109,548 cases and 277,440 controls (heritability: 7.2%) [133]  (6) Ischemic stroke (IS): 67,162 cases and 454,450 controls (heritability: not reported in the original GWAS publication) [134]  (7) Multiple sclerosis (MS): 47,429 cases and 68,374 controls (heritability: 19.2%) [135]  (8) Parkinson's disease (PD): 33,674 cases and 449,056 controls (heritability: 22%-27%) [136]   - Psychiatric disorders   (1) Attention-deficit/hyperactivity disorder (ADHD): 20,183 cases and 35,191 controls (heritability: 21.6%) [137]  (2) Anxiety disorder (ANX): 25,453 cases and 58,113 controls (heritability: 26%) [138]  (3) Autism spectrum disorder (ASD): 18,381 cases and 27,969 controls (heritability: 11.8%) [139]  (4) Alcohol use disorder (AUD): 11,569 cases and 34,999 controls (heritability: 9.0%) [140]  (5) Bipolar disorder (BIP): 41,917 cases and 371,549 controls (heritability: 18.6%) [141]  (6) Major depressive disorder (MDD): 135,458 cases and 344,901 controls (heritability: 8.7%) [142]  (7) Obsessive-compulsive disorder (OCD): 2,688 cases and 7,037 controls (heritability: 28%) [143]  (8) Post-traumatic stress disorder (PTSD): 9,831 cases and 19,225 controls (heritability: 28%) [144]  (9) Schizophrenia (SCZ): 76,755 cases and 243,649 controls (heritability: 24%) [145]  (10) Tourette syndrome (TS): 4,819 cases and 9,488 controls (heritability: 21%) [146]  The brain volume GWAS summary statistics were processed by Zhao et al. [147] from 19,629 UK Biobank participants of European ancestry (reported mean heritability ~40%). Volumes included total brain volume, gray matter, white matter, and cerebrospinal fluid, labeled with the Mindboggle-101 atlas [148]. The white matter tract GWAS summary statistics were processed by Zhao et al. [22] from 33,292 UK Biobank participants of European ancestry (reported mean heritability ~46.3%). |
|  | b) | Report summary statistics for phenotypic exposure(s), outcome(s), and other relevant variables (e.g. means, SDs, proportions) | S4 Table, S6 Table | *Note: This two tables summarized the main MR analysis results.* |
|  | c) | If the data sources include meta-analyses of previous studies, provide the assessments of heterogeneity across these studies | - | *Note: Our analysis results don’t include meta-analyses of previous studies.* |
|  | d) | For two-sample MR:  i.  Provide justification of the similarity of the genetic variant-exposure associations between the exposure and outcome samples  ii.  Provide information on the number of individuals who overlap between the exposure and outcome studies | 6, 27-29 | All individuals fell within three standard deviations of the mean for the first and second principal components of European-ancestry populations from the 1000 Genomes Project [12].  The brain volume GWAS summary statistics were processed by Zhao et al. [147] from 19,629 UK Biobank participants of European ancestry (reported mean heritability ~40%). Volumes included total brain volume, gray matter, white matter, and cerebrospinal fluid, labeled with the Mindboggle-101 atlas [148]. The white matter tract GWAS summary statistics were processed by Zhao et al. [22] from 33,292 UK Biobank participants of European ancestry (reported mean heritability ~46.3%).  Most were published recently and derived from meta-analyses with large sample sizes (mean ~280,000 individuals). All GWAS summary statistics were of European ancestry, and there was no participant overlap with the cell type–specific *cis*-eQTL dataset.  We manually verified the sample descriptions of all datasets and confirmed the absence of participant overlap between the *cis*-eQTL catalog and the GWAS cohorts. |
| 11 | **Main results** |  |  |  |
|  | a) | Report the associations between genetic variant and exposure, and between genetic variant and outcome, preferably on an interpretable scale | S4 Table, S6 Table | *Note: This two tables summarized the main MR analysis results.* |
|  | b) | Report MR estimates of the relationship between exposure and outcome, and the measures of uncertainty from the MR analysis, on an interpretable scale, such as odds ratio or relative risk per SD difference | S4 Table, S6 Table | *Note: This two tables summarized the main MR analysis results, and the measures of uncertainty from the MR analysis.* |
|  | c) | If relevant, consider translating estimates of relative risk into absolute risk for a meaningful time period | - | *Note: Given the nature of our study and the focus on genetic instruments, we did not calculate absolute risk estimates, as our primary aim was to assess relative risk associations.* |
|  | d) | Consider plots to visualize results (e.g. forest plot, scatterplot of associations between genetic variants and outcome versus between genetic variants and exposure) | Fig 1b, Fig 1c, Fig 2, Fig 3, S8-S10 Figs | *Note: We have included several plots to visualize the MR results, including heatmaps, upset plots, and dumbbell plots.* |
| 12 | **Assessment of assumptions** |  |  |  |
|  | a) | Report the assessment of the validity of the assumptions | S4 Table, S6 Table | *Note: We reported the results from heterogeneity and pleiotropy tests to detect the presence of heterogeneity and horizontal pleiotropy in IVW results in S4 and S6 Table.* |
|  | b) | Report any additional statistics (e.g., assessments of heterogeneity across genetic variants, such as *I^2^*, Q statistic or E-value) | S4 Table, S6 Table | *Note: We reported the results from heterogeneity and pleiotropy tests to detect the presence of heterogeneity and horizontal pleiotropy in IVW results in S4 and S6 Table.* |
| 13 | **Sensitivity analyses and additional analyses** |  |  |  |
|  | a) | Report any sensitivity analyses to assess the robustness of the main results to violations of the assumptions | 30-31, S4 Table, S6 Table | SMR incorporates the HEIDI test to detect linkage [6]; results with HEIDI *P* < 0.05 were discarded.  The *harmonise_data()* function in TwoSampleMR was used to harmonize effect alleles and SNP effects between exposure and outcome. Heterogeneity (function *mr_heterogeneity*, *P* < 0.05) and pleiotropy (function *mr_pleiotropy_test*, P < 0.05) tests were conducted for IVW results.  GSMR incorporates the HEIDI-outlier test to identify and exclude instruments with significant pleiotropic effects on the outcome (*P* < 0.05). Both GSMR and the HEIDI-outlier test were implemented with the R package *gsmr* (version 1.1.0). |
|  | b) | Report results from other sensitivity analyses or additional analyses | 8-9 | To assess reproducibility, we replicated the MR analyses using cortical eQTLs from BrainMeta [36] and cell type–specific eQTLs from brainSCOPE [37]. Of the eGene–IDP pairs, 64.9% (430/663) and 49.2% (326/663) were replicated in BrainMeta at nominal and FDR < 0.05 levels, respectively (both *P* < 10^-16^, hypergeometric test; **S5 Table**; **S2 Fig**). For eGene–cell type–IDP triples, 41.9% (315/751) and 33.7% (253/751) were replicated in brainSCOPE at nominal and FDR < 0.05 levels, respectively (both *P* < 10^-16^, hypergeometric test; **S5 Table**; **S3 Fig**).  We validated these findings using three independent resources: BrainMeta cortical eQTLs [36], PsychENCODE differential expression data [29], and the GWAS Catalog [1]. Replication rates were 35.7% (87/244) and 23.0% (56/244) in BrainMeta at nominal and FDR < 0.05 levels, respectively; 29.5% (23/78) in PsychENCODE; and 23.8% (57/240) in the GWAS Catalog (all *P* < 10^-4^, hypergeometric test; **S7 Table**; **S2 Fig**). For eGene–cell type–DB triples, replication in brainSCOPE [37] was 41.9% (315/751) nominally and 33.7% (253/751) at FDR < 0.05 (both with *P* < 10^-16^, hypergeometric test; **S7 Table**; **S3 Fig**). Notably, 12 of the 14 AD-associated cell type–specific causal eGenes identified here were corroborated by Mathys et al. [45]. |
|  | c) | Report any assessment of direction of causal relationship (e.g., bidirectional MR) | 16-17 | To dissect these causal chains at cellular resolution, we performed bidirectional Mendelian randomization between IDPs and DBs (**Methods**). This identified six causal effects from IDPs to DBs and three from DBs to IDPs. By overlaying these bidirectional phenotype relationships with the cell type–specific eGene–phenotype associations identified above, we reconstructed four eGene–IDP–DB routes and seven eGene–DB–IDP routes in which the eGene was causal for both the imaging phenotype and the disorder (**Fig. 4**; **S12 Table**). |
|  | d) | When relevant, report and compare with estimates from non-MR analyses | 8-9 | To assess reproducibility, we replicated the MR analyses using cortical eQTLs from BrainMeta [36] and cell type–specific eQTLs from brainSCOPE [37]. Of the eGene–IDP pairs, 64.9% (430/663) and 49.2% (326/663) were replicated in BrainMeta at nominal and FDR < 0.05 levels, respectively (both *P* < 10^-16^, hypergeometric test; **S5 Table**; **S2 Fig**). For eGene–cell type–IDP triples, 41.9% (315/751) and 33.7% (253/751) were replicated in brainSCOPE at nominal and FDR < 0.05 levels, respectively (both *P* < 10^-16^, hypergeometric test; **S5 Table**; **S3 Fig**).  We validated these findings using three independent resources: BrainMeta cortical eQTLs [36], PsychENCODE differential expression data [29], and the GWAS Catalog [1]. Replication rates were 35.7% (87/244) and 23.0% (56/244) in BrainMeta at nominal and FDR < 0.05 levels, respectively; 29.5% (23/78) in PsychENCODE; and 23.8% (57/240) in the GWAS Catalog (all *P* < 10^-4^, hypergeometric test; **S7 Table**; **S2 Fig**). For eGene–cell type–DB triples, replication in brainSCOPE [37] was 41.9% (315/751) nominally and 33.7% (253/751) at FDR < 0.05 (both with *P* < 10^-16^, hypergeometric test; **S7 Table**; **S3 Fig**). Notably, 12 of the 14 AD-associated cell type–specific causal eGenes identified here were corroborated by Mathys et al. [45]. |
|  | e) | Consider additional plots to visualize results (e.g., leave-one-out analyses) | S2 Fig, S3 Fig | *Note: We performed replication analysis on the predicted causal associations between eGenes and brain-associated complex traits identified in our MR analysis. The results are shown in S2 Fig and S3 Fig.* |
|  | **DISCUSSION** |  |  |  |
| 14 | **Key results** | Summarize key results with reference to study objectives | 23 | In this study, we employed a single-cell Mendelian randomization framework to dissect potential causal relationships between gene expression and brain-associated complex phenotypes, leveraging complementary analytical methods to enhance reliability. We identified 254 and 217 eGenes with putative causal effects on 112 IDPs and 26 DBs, respectively, across eight brain cell types. These causal eGenes exhibited strong cell type specificity: over 90% of eGene–IDP associations and approximately 80% of eGene–DB associations were restricted to a single cell type. At the same time, the causal eGenes showed widespread phenotype pleiotropy, exemplified by *DDHD2* in excitatory neurons, *XKR6* in inhibitory neurons, and *MAPT* and *ZSCAN31* in astrocytes. Genes shared across different categories of brain-associated phenotypes displayed distinct patterns of evolutionary constraint and cell type enrichment, and were overrepresented in biological processes including memory and cognition, neurotransmitter regulation, and cellular maintenance and signaling. We further characterized putative causality routes linking cell type–specific causal eGenes to IDPs and DBs, and, by examining the spatiotemporal expression dynamics of these genes in independent single-cell data, uncovered coordinated transcriptional programs that underpin the relationships among brain-related complex phenotypes. |
| 15 | **Limitations** | Discuss limitations of the study, taking into account the validity of the IV assumptions, other sources of potential bias, and imprecision. Discuss both direction and magnitude of any potential bias and any efforts to address them | 26 | Several avenues remain open for future work. First, the comparatively small number of *cis*-eQTLs in endothelial cells and pericytes (**S3 Table**) [12], likely constrained the number of causal genes identified in these cell types (**Fig. 1b**). Larger single-cell eQTL datasets with greater sample sizes and finer cell type resolution should improve power to detect eGene–phenotype associations in these populations. Second, when performing MR between IDPs and DBs, half of the DB GWASs were excluded because they included participants from UK Biobank [123], limiting the completeness of the inferred causality routes among eGenes, IDPs, and DBs (**Fig. 4**). In addition, shared causal eGenes identified across IDPs and/or DBs may be influenced by unavoidable sample overlap between some GWAS datasets. Future studies should assemble more fully independent GWAS resources for brain-associated phenotypes to strengthen the robustness and coverage of these causal inferences. Third, although our findings highlight putative causal relationships, they do not establish definitive causality and require confirmation by randomized controlled trials, the gold standard for causal inference. Considerable work remains ahead in treatment development, and our results should be interpreted accordingly. |
| 16 | **Interpretation** |  |  |  |
|  | a) | Meaning: Give a cautious overall interpretation of results in the context of their limitations and in comparison with other studies | 26 | Third, although our findings highlight putative causal relationships, they do not establish definitive causality and require confirmation by randomized controlled trials, the gold standard for causal inference. Considerable work remains ahead in treatment development, and our results should be interpreted accordingly. |
|  | b) | Mechanism: Discuss underlying biological mechanisms that could drive a potential causal relationship between the investigated exposure and the outcome, and whether the gene-environment equivalence assumption is reasonable. Use causal language carefully, clarifying that IV estimates may provide causal effects only under certain assumptions | 24 | In our analysis, six eGenes—*SYT14*, *MSH3*, *ICA1L*, *RGS14*, *C17orf97* and *ZSCAN31*—were causally associated with AD in excitatory neurons (**S6 Table**). Among these, five (*MSH3*, *ICA1L*, *RGS14*, *C17orf97* and *ZSCAN31*) showed a consistent positive causal effect. Several of these genes have established links to excitatory neuron or synaptic function: *SYT14* mediates calcium-dependent neurotransmitter release and synaptic vesicle trafficking [117, 118]; *ICA1L* and *RGS14* modulate synaptic signaling pathways in excitatory neurons [97, 119]; and *ZSCAN31*, a zinc finger protein, regulates neuronal excitability and intrinsic circuitry within this subtype [120, 121]. This convergence suggests that these genes may influence AD progression through shared mechanisms affecting neuronal function. |
|  | c) | Clinical relevance: Discuss whether the results have clinical or public policy relevance, and to what extent they inform effect sizes of possible interventions | 26 | Third, although our findings highlight putative causal relationships, they do not establish definitive causality and require confirmation by randomized controlled trials, the gold standard for causal inference. Considerable work remains ahead in treatment development, and our results should be interpreted accordingly.  In summary, our study provides a systematic investigation of cell type–specific causal genes for brain-associated complex phenotypes, together with evidence that brain structure and disorders/behaviors share genetic foundations operating at the cellular level. We hope that the connections identified among genes, cell types, and complex phenotypes will serve as starting points for developing therapeutic strategies targeting brain structural abnormalities, disorders, and behaviors. |
| 17 | **Generalizability** | Discuss the generalizability of the study results (a) to other populations, (b) across other exposure periods/timings, and (c) across other levels of exposure | 26 | First, the comparatively small number of *cis*-eQTLs in endothelial cells and pericytes (**S3 Table**) [12], likely constrained the number of causal genes identified in these cell types (**Fig. 1b**). Larger single-cell eQTL datasets with greater sample sizes and finer cell type resolution should improve power to detect eGene–phenotype associations in these populations. |
|  | **OTHER INFORMATION** |  |  |  |
| 18 | **Funding** | Describe sources of funding and the role of funders in the present study and, if applicable, sources of funding for the databases and original study or studies on which the present study is based | 36 | This work was partly supported by National Natural Science Foundation of China (T2225015, 62433008), Shanghai Science and Technology Commission Program (23JS1410100, 24JS2810100), Shanghai Municipal Education Commission (24KXZNA11), Major Project of Guangzhou National Laboratory (GZNL2024A01003), National Key R&D Program of China (2023YFF1204800, 2025YFC3409300, 2025YFA1309200). The computations in this research were performed using the CFFF platform of Fudan University. The funders had no role in study design, data collection and analysis, decision to publish, or preparation of the manuscript. |
| 19 | **Data and data sharing** | Provide the data used to perform all analyses or report where and how the data can be accessed, and reference these sources in the article. Provide the statistical code needed to reproduce the results in the article, or report whether the code is publicly accessible and if so, where | 34-35 | Data Availability Statement The scripts in the study are publicly available via GitHub at https://github.com/sldrcyang/ExpMR. The predicted cell type-specific causal eGenes, and their associated IDPs and DBs can be freely downloaded in our supplementary website (https://mai.fudan.edu.cn/expmr). The cell type-specific cis-eQTL summary statistics dataset was downloaded from https://zenodo.org/record/6104982#.Y1a2WbZBxPY. The GWAS summary statistics datasets are available from the URLs: CI from http://ssgac.org/documents/CHIC_Summary_Benyamin2014.txt.gz; EA from https://zenodo.org/records/18584527/files/GWAS_EA_excl23andMe.zip?download=1; INT from https://ctg.cncr.nl/documents/p1651/SavageJansen_IntMeta_sumstats.zip; NEU from https://ctg.cncr.nl/documents/p1651/sumstats_neuroticism_ctg_format.txt.gz; ASP from https://zenodo.org/records/18584527/files/AUTOMOBILE_SPEEDING_PROPENSITY_GWAS.zip?download=1; DPW from https://conservancy.umn.edu/handle/11299/201564; GRT from https://zenodo.org/records/18584527/files/RISK_behavior.RISK_GWAS_MA_UKB+replication.zip?download=1; SC from https://conservancy.umn.edu/handle/11299/201564; INS from https://ctg.cncr.nl/documents/p1651/insomnia_ukb2b_EUR_sumstats_20190311_with_chrX_mac_100.txt.gz; EPI from https://www.epigad.org/gwas_ilae2018_16loci/all_epilepsy_METAL.gz; ICH from https://personal.broadinstitute.org/ryank/3980413.Woo.2014.zip; IS from http://megastroke.org/; AD from https://ctg.cncr.nl/documents/p1651/AD_sumstats_Jansenetal.txt.gz; ALS from https://zenodo.org/records/18584527/files/alsMetaSummaryStats_march21st2018.tab.zip?download=1; MS from https://imsgc.net/data/discovery_metav3.0.meta.gz; PD from https://zenodo.org/records/18584527/files/nallsEtAl2019_excluding23andMe_allVariants.tab.zip?download=1; OCD from https://doi.org/10.6084/m9.figshare.14672103; TS from https://doi.org/10.6084/m9.figshare.14672232; ANX from https://zenodo.org/records/18584527/files/TotAnx_OR_sumstats.zip?download=1; MDD from https://doi.org/10.6084/m9.figshare.14672085; ADHD from https://doi.org/10.6084/m9.figshare.14671965; ASD from https://doi.org/10.6084/m9.figshare.14671989; AUD from https://doi.org/10.6084/m9.figshare.14672187; PTSD from https://ipsych.dk/fileadmin/ipsych.dk/Downloads/daner_woautism_ad_sd8-sd6_woautismstress_cleaned.gz; BIP from https://doi.org/10.6084/m9.figshare.14102594; SCZ from https://doi.org/10.6084/m9.figshare.19426775; IDPs from https://www.med.unc.edu/bigs2/data/gwas-summary-statistics/. The pLI scores are downloaded from ExAc (https://genome.ucsc.edu/cgi-bin/hgTrackUi?db=hg19&g=gnomadPLI; version v2.1.1). The prediction weights and covariance used for TWAS analysis are downloaded from PredictDB Data Repository (https://predictdb.org/), and CMC-derived DLPFC prediction models (https://github.com/laurahuckins/CMC_DLPFC_prediXcan). The cis-eQTL summary statistics of the BrainMeta portal are downloaded from https://yanglab.westlake.edu.cn/data/brainmeta/cis_eqtl/. The documented associations between genes and complex phenotypes of the NHGRI-EBI GWAS Catalog are downloaded from https://www.ebi.ac.uk/gwas/api/search/downloads/associations/v1.0.2?split=false. The cell type-specific eQTL summary statistics from the brainSCOPE resource are downloaded from https://brainscope.gersteinlab.org/integrative_files.html. The differentially expressed genes associated with AD from Mathys et al. 2023 are downloaded from https://github.com/mathyslab7/ROSMAP_snRNAseq_PFC/tree/main/Results/Differential_gene_expression_analysis. |
| 20 | **Conflicts of Interest** | All authors should declare all potential conflicts of interest | 36 | The authors have declared that no competing interests exist. |

This checklist is copyrighted by the Equator Network under the Creative Commons Attribution 3.0 Unported (CC BY 3.0) license.

1. Skrivankova VW, Richmond RC, Woolf BAR, Yarmolinsky J, Davies NM, Swanson SA, et al. Strengthening the Reporting of Observational Studies in Epidemiology using Mendelian Randomization (STROBE-MR) Statement. JAMA. 2021.

2. Skrivankova VW, Richmond RC, Woolf BAR, Davies NM, Swanson SA, VanderWeele TJ, et al. Strengthening the Reporting of Observational Studies in Epidemiology using Mendelian Randomisation (STROBE-MR): Explanation and Elaboration. BMJ. 2021;375:n2233.
