# Supplementary material for: Deciphering cell type-specific causal genetic effects on brain imaging-derived phenotypes and disorders with single-cell Mendelian randomization
Source: PLoS Comput Biol. 2026 Jun 17;22(6):e1014422. doi: 10.1371/journal.pcbi.1014422 (PMC13289931; doi:10.1371/journal.pcbi.1014422)
Supplement: Attachment — Submitted filename: Response to Reviewers.docx [file pcbi.1014422.s038.docx]

College of Biomedical Engineering

Fudan University

Shanghai 200438, China

February 17, 2026

Dear Editor,

Thank you for your feedback on our manuscript entitled “**Deciphering cell type-specific causal genetic effects on brain imaging-derived phenotypes and disorders with single-cell Mendelian randomization**” (PCOMPBIOL-D-25-00949). We sincerely thank all reviewers for their constructive and valuable feedback that we have used to improve the quality of our manuscript.

The reviewers’ comments have been numbered and laid out in italicized font, while our point-to-point response in normal font. Notably, any changes or modifications in the main text or supplementary text have been given in red.

We are hopeful that the revised manuscript would be regarded as appropriate for **PLOS Computational Biology** publication. Thank you again for your consideration.

Best regards,

**Dr. Xing-Ming Zhao**

Professor

College of Biomedical Engineering

Fudan University

Shanghai, P.R. China

Tel. 86-21-55665546

Email: xmzhao@fudan.edu.cn

*Response Letter to*

**Deciphering cell type-specific causal genetic effects on brain imaging-derived phenotypes and disorders with single-cell Mendelian randomization**

# *Reviewer #1:*

*Summary: Overall, a very interesting article with findings pertinent to the field. While I have some analytical concerns, I believe these can be addressed, and overall, I recommend this article to be accepted with minor revisions.*

**REF1 – Response to REF1:** We sincerely thank you for these constructive, valuable and professional comments, which have greatly benefited us. The detailed point-by-point responses are listed below.

**REF1 – Point to point response to REF1**

## REF 1.1 – Issue of tissue matching in MR

| *Referee Comment* | *One potential flaw in the analytical workflow is that the authors use cell type-specific eQTLs from prefrontal cortex, temporal cortex, and deep white matter to infer associations about other brain regions. The literature on whether this is appropriate is currently in flux. Currently the data suggests that the same cell type in different cortical regions in generally consistent (PMID: 39578476), but this is not necessarily the case subcortically (PMID:* *37824663). Since there are few resources for single-cell data for most brain regions (excluding the dorsolateral prefrontal cortex), I recommend the authors limit their broader analyses to the brain regions utilized in the eQTL data and validate particular associations in subcortical brain regions by other means.* |
| --- | --- |
| Author Response | We thank the reviewer for raising this important point. We agree that the *cis*-eQTLs used in our study come from the prefrontal cortex, temporal cortex, and white matter, which do not cover all brain regions. However, in the Mendelian randomization (MR) framework, tissue matching is not required to test whether an exposure has a causal effect on an outcome. According to *Sanderson et al.*, as long as the genetic instruments satisfy the three core instrumental variable (IV) assumptions (relevance, independence, and exclusion restriction) and an additional point-estimate-identifying assumption (e.g., the genetic variant affects the exposure in the same direction across all individuals, typically assumed in standard MR models), it’s sufficient to validly test the null hypothesis of no causal effect and the resulting expression-outcome link is biologically interpretable(1). These assumptions relate to the validity of the genetic instruments and the structure of the gene-trait relationship, rather than tissue specificity.  Using gene expression data from related but not identical tissues is commonly used in studies of MR, TWAS, and colocalization analyses. For example, *Chen et al.* used blood-derived eQTLs to identify genes associated with nearly 4,000 brain imaging phenotypes via SMR and colocalization(2). *Zhou et al.* proposed a cross-tissue TWAS-MR joint framework that models shared *cis*-eQTL effects across tissues to improve gene expression prediction and identify causal genes for brain traits(3). *Zhao et al.* performed cross-tissue TWAS analysis, linking gene expression in individual cortical regions with 211 neuroimaging traits spanning cortical, subcortical, and cerebellar structures(4).  In the revised manuscript, we have added a section to introduce MR and clarified the MR assumptions in plain text in **S3 Text**. |
| Excerpt 1.1 (Supplementary Text) | **S3 Text:**  In general, effective MR methods rely on three core assumptions: (i) Relevance: each genetic variant is associated with the exposure; (ii) Independence: each genetic variant is independent of confounding factors; (iii) Exclusion restriction: each genetic variant influences the outcome only through the exposure. For MR methods such as MR-Egger that explicitly model horizontal pleiotropy (variants influence the outcome directly), they relax the third assumption and instead require the weaker InSIDE (Instrument Strength Independent of Direct Effect) condition^6^. Because violations of these assumptions can bias causal estimates, MR analyses typically include tests for horizontal pleiotropy, weak instrument bias, and heterogeneity to ensure robustness^6-9^. As long as the genetic instruments satisfy the three core IV assumptions and an additional point-estimate-identifying assumption (e.g., the genetic variant affects the exposure in the same direction across all individuals, typically assumed in standard MR models), the null hypothesis of no causal effect is sufficient to test validly and the resulting exposure-outcome relationship is biologically interpretable^5^. |

## REF 1.2 – Modifying Fig. 1b

| *Referee Comment* | *Regarding figure 1B., it is difficult to visually identify between 0 and 1 eGenes according to the color scale. I recommend using a distinct color for 0 and then using a color bar to annotate between 1 and the maximum value.* |
| --- | --- |
| Author Response | Thank you for this suggestion. In the revised manuscript, we have updated **Fig. 1b** by using separate light beige to denote zero and use a continuous color bar to denote between 1 and the maximum value. |
| Excerpt 1.2 (Main Text) |   **Fig. 1. (b)** Heatmap showing the number of putatively causal cell type-specific eGenes across IDPs. |

## REF 1.3 – Clarifying how FDR correction was applied

| *Referee Comment* | *"As shown in Fig. 1b and S4 Table, we identified 254 eGenes that may have causal effects on 112 IDPs in eight cell types (FDR < 0.05)..." Can the authors clarify how FDR correction was applied? I would apply it across all IDPs+DBs and cell types.* |
| --- | --- |
| Author Response | Thank you for this suggestion. In the revised manuscript, we have clarified that the FDR correction was applied across all genes × cell types × IDPs combinations for IDPs, and across all genes × cell types × DBs combinations for DBs, respectively. We chose to correct IDPs and DBs separately because they represent fundamentally distinct types of phenotypes, derived from different GWAS sources, with different statistical power, correlation structures, and association distributions. Pooling them into a single correction might mix unrelated signals, make true associations harder to detect, and obscure interpretation. Similar strategy of applying FDR correction within phenotype classes has been adopted in previous studies. For example, *Yang et al.* applied FDR correction separately for brain imaging phenotypes and behavioral traits(5). |
| Excerpt 1.3 (Main Text) | **Lines 160-162 (Results):**  As shown in **Fig. 1b** and **S4 Table**, we identified 254 eGenes that may have causal effects on 112 IDPs in eight cell types (FDR < 0.05, corrected across all gene-cell type-IDP tests), forming a catalog of 760 combinations between cell types, eGenes and IDPs.  **Lines 184-186 (Results):**  As shown in **Fig. 1c** and **S6 Table**, we identified 217 eGenes that may have causal effects on 26 DBs in eight cell types (FDR < 0.05, corrected across all gene-cell type-DB tests), forming a catalog of 298 combinations between cell types, eGenes and DBs. |

## REF 1.4 – Correlational structure of IDPs

| *Referee Comment* | *Regarding the FDR correction, I would also be interested in understanding the correlation structure of the IDPs. Could the authors provide a supplementary figure detailing this? If it’s found that the* *correlational structure is interfering with the FDR correction, perhaps dimensionality reduction can be used to create representative categories of multiple IDPs? This is also especially critical to understand within class trait pleiotropy, as detailed in the section, "Pervasive trait pleiotropy of causal eGenes".* |
| --- | --- |
| Author Response | Thank you for this suggestion. Our study adopts a two-sample MR framework, where IDPs serve as outcome traits, and only GWAS summary statistics (i.e., SNP-trait associations) are used. Since we do not use raw individual-level IDP data, dimensionality reduction techniques are not applicable in our setting. Moreover, as discussed in **REF 1.1**, as long as IV assumptions and a point-estimate identifying assumption are satisfied, the MR test of a causal effect is valid regardless of the correlation structure among outcomes. In other words, the correlation structure among IDPs does not bias MR estimates or FDR correction, provided the tests themselves are well-calibrated.  It is well known that brain regions exhibit highly structural, functional, and genetic correlations(6-8), and shared associated genes across IDPs are reported in prior studies(9). In this context, the presence of shared causal eGenes across IDPs is expected and reflects genuine shared biology rather than artifacts introduced by FDR correction procedures. Notably, many existing studies adopt FDR correction across large number of IDPs without adjusting for their correlation structure(5, 10).  To address the reviewer’s request, we have added to compute the pairwise genetic correlation matrix among the 123 IDPs using LD Score Regression (LDSC) based on GWAS summary statistics (**S7 Fig**)(11).The correlations range from -0.83 to 1, indicating the extensive shared genetic architecture among brain regions. As our manuscript only discusses shared causal eGenes between two IDP groups (white matter microstructure phenotype and brain regional volume), we specifically examined their cross-group genetic correlations. The highest correlation is observed between the posterior limb of internal capsule (PLIC) and right lateral ventricle (*rg* = 0.49). However, the two traits do not share any causal eGenes in our results (**S4 Table**), suggesting that shared causal genes are not simply a byproduct of IDP correlations, but may reflect specific underlying regulatory mechanisms (**Lines 226-228**).    **S7 Fig. Genetic correlation among imaging-derived phenotypes (IDPs).** Pairwise genetic correlations were estimated for all 123 IDPs using LD Score Regression based on GWAS summary statistics(11). Correlations range from -0.83 to 1. The strongest correlation between IDPs from different groups was observed between the FA value of posterior limb of the internal capsule (PLIC_FA) and the volume of right lateral ventricle (*rg* = 0.49).  Finally, we clarify that our main findings focus more on cross-class pleiotropy between IDPs and DBs, rather than within-class pleiotropy among IDPs. The latter is briefly mentioned and is not central to our primary findings. |
| Excerpt 1.4 (Main Text) | **Lines 226-228 (Results):**  Notably, this pleiotropy is not simply driven by genetic correlations among IDPs, as even the most correlated cross-group pair (PLIC_FA and right lateral ventricle, *rg* = 0.49) shares no causal eGenes (**S7 Fig**). |

## REF 1.5 – Replication criteria clarification

| *Referee Comment* | *"found that 76.3% (505 out of 662; P < 10-16, hypergeometric test) of the eGene-IDP pairs could be successfully replicated ..." It would be helpful to state that this replication is at a nominal level. Additionally, if any of the methods provide directionality, it would be helpful to confirm the directionality matches.* |
| --- | --- |
| Author Response | Thanks for this comment. In the revised manuscript, we added to state that this replication was assessed at a nominal threshold (*P* < 0.05) (**Lines 166-168, Lines 191-193**). Furthermore, we refined our replication criteria in BrainMeta dataset to also require directional consistency: a gene-trait pair is considered replicated in the BrainMeta only if it reaches *P* < 0.05 using at least one MR method, and shows the same effect direction across all MR methods and as observed in the discovery analysis. The updated replication results for BrainMeta remain consistent with the original results (**Lines 166-168, Lines 191-193**, **S2 Fig**):   - 64.9% (430/663; *P* < 10^-16^, hypergeometric test) of eGene-IDP pairs were replicated at *P* < 0.05, and 49.2% (326/663; *P* < 10^-16^, hypergeometric test) were replicated at FDR < 0.05. - 35.7% (87/244; *P* < 10^-16^, hypergeometric test) of the eGene-DB pairs were replicated at *P* < 0.05, and 23% (56/244; *P* < 10^-16^, hypergeometric test) were replicated at FDR < 0.05.     **S2 Fig. Replication results for the predicted gene-IDP and gene-DB associations.** Reproducibility of eGene-IDP pairs was assessed using cortical *cis*-eQTL summary statistics from BrainMeta(12). Reproducibility of eGene-DB pairs was assessed using cortical *cis*-eQTLs from BrainMeta(12), differentially expressed genes from PsychENCODE(13), and gene-trait associations from GWAS Catalog(14). Significance of overlap was tested by hypergeometric test. |
| Excerpt 1.5 (Main Text) | **Lines 164-168 (Results):**  To confirm the reproducibility of the predicted eGene-IDP pairs and eGene-cell type-IDP triples, we repeated MR analyses using cortical eQTL summary statistics from BrainMeta [36] and cell type-specific eQTL summary statistics from brainSCOPE [37], respectively. Among the eGene-IDP pairs, 64.9% (430/663) and 49.2% (326/663) were replicated in BrainMeta at nominal and FDR < 0.05 levels, respectively (both *P* < 10^-16^, hypergeometric test; **S5 Table** and **S2 Fig**).  **Lines 188-193 (Results):**  As we did for the IDPs, we confirmed the reproducibility of eGene-DB pairs using three datasets: the cortical eQTL summary statistics from BrainMeta [36], differentially expressed genes and transcripts from PsychENCODE [29], and documented gene-trait associations from GWAS Catalog [1]. Replication rates were 35.7% (87/244; at nominal significance level) and 23.0% (56/244; at FDR < 0.05 level) in BrainMeta, 29.5% (23/78) in PsychENCODE, and 23.8% (57/240) in the GWAS Catalog (all *P* < 10^-4^, hypergeometric test; **S7 Table** and **S2 Fig**).  **Methods (Lines 834-846):**  To validate the predicted eGene–trait pairs, three external datasets were used. (1) We obtained the *cis*-eQTL summary statistics from the BrainMeta portal (https://yanglab.westlake.edu.cn/data/brainmeta/cis_eqtl/), which was derived using RNA sequencing data of 2,865 brain cortex samples from 2,443 unrelated individuals of European ancestry [36]. The same MR analysis procedure was applied to this data to infer the causal relationships between cortical gene expression and 149 brain-associated complex traits. An eGene–trait pair was considered replicated if it achieved nominal significance in at least one MR method, and showed consistent effect directions across all MR methods and with the discovery analysis. (2) We extracted differentially expressed genes and transcripts (at 5% FDR) in ASD, SCZ and BIP from PsychENCODE [29], which leveraged in vivo gene expression profiles to pinpoint specific alterations in transcriptome-wide isoform levels across the three major psychiatric disorders. (3) We obtained published associations between genes and complex traits from the NHGRI-EBI GWAS Catalog (version 2023-06-09, www.ebi.ac.uk/gwas/) [1]. |

## REF 1.6 – Clarifying the cell type specificity of eGene-trait pairs

| *Referee Comment* | *"Our predicted eGene-IDP pairs exhibited strong cell type specificity, with 91.4% (605 out of 662) existing only in a particular cell type, which is consistent with previous literature showing that the formation of brain structures is modulated by genes in a cell type-specific manner[37-39]." Can the authors clarify whether this rate is above what we would expect considering the underlying cell type specificity of the eQTLs themselves? Also, please clarify this for both IDP and DB analyses.* |
| --- | --- |
| Author Response | Thanks for this comment. To test whether our observed high cell type specificity of predicted eGene-trait associations was simply driven by the eQTLs themselves, we performed an additional analysis restricted to genes that have valid instrumental *cis*-eQTLs in ≥ 2 cell types. This ensures that the observed specificity cannot be simply attributed to a gene being testable in only one cell type. Under this restriction, 80.6% (237 out of 294) of the predicted eGene-IDP associations were significant in exactly one cell type; 69.2% (92 out of 133) eGene-DB associations were significant in exactly one cell type (**S4 Fig**). Additionally, our findings are consistent with prior literature reporting that 81.9% of genes have putative causal associations with central nervous system traits were identified in a single brain cell type(15). These demonstrate that the high cell-type specificity persists even when the same gene has instruments in multiple cell types, suggesting that this specificity reflects biologically meaningful regulatory mechanisms rather than eQTL themselves. We have now incorporated this additional analysis in the revised manuscript (**Lines 173-175**, **Lines 200-202**).    **S4 Fig. Distribution of cell type specificity for predicted eGene-trait associations.** Bar plots show how many eGene-trait associations are predicted in exactly *k* cell types (x-axis) (restricted to eGenes with valid instrumental *cis*-eQTLs in two or more cell types). The y-axis shows the number of predicted eGene-trait associations that appear in exactly *k* cell types. |
| Excerpt 1.6 (Main Text) | **Lines 173-175 (Results):**  The high specificity remained after controlling for eQTL availability. Among predicted eGene-IDP pairs where the gene had valid instrumental *cis*-eQTLs in two or more cell types, 80.6% (237 out of 294) were significant in exactly one cell type (**S4 Fig**).  **Lines 200-202 (Results):**  Among predicted eGene-DB pairs where the gene had valid instrumental *cis*-eQTLs in two or more cell types, 69.2% (92 out of 133) were significant in exactly one cell type (**S4 Fig**). |

## REF 1.7 – Use of SNPs in MHC region and risks from high LD

| *Referee Comment* | *"In contrast, the causal eGenes shared within the brain regional volume group were generally involved in protein binding, such as Hsp90 protein binding and MHC class I protein binding (Fig. 2c and S7 Table), which aligns with previous findings showing that the expression of Hsp90 and MHC class I proteins could regulate synapse formation and brain development[55, 56]." Could the authors clarify whether SNPs within the MHC were used to identify eGenes? The high linkage disequilibrium of this region requires careful analysis to make sure genetic associations are not spurious.* |
| --- | --- |
| Author Response | Thanks for this comment. We did not pre-exclude SNPs within MHC region, as it is standard practice to retain such regions initially and apply linkage disequilibrium (LD) clumping steps, LD-corrected models, or downstream horizontal pleiotropy tests to minimize biased results due to LD(16-18). In our analysis, we implemented several models and layers of safeguards to address potential bias caused by LD:  (1) For analysis using SMR, only the top *cis*-eQTL for each gene was used as the instrument, and the HEIDI test was applied to detect and remove associations driven by LD rather than causality;  (2) For analysis using Wald ratio and IVW, strict LD clumping (*r^2^* < 0.1) was applied to select approximately independent instruments, and the heterogeneity and pleiotropy tests were conducted to detect the presence of heterogeneity and horizontal pleiotropy in MR results;  (3) For analysis using PMR-Egger, the model is specially designed to be robust to correlated instruments and model LD-induced pleiotropy;  (4) For analysis using PMR-Egger, LD matrix has already been incorporated in the model and HEIDI-outlier test was performed to remove SNPs showing evidence of pleiotropic effects on the outcome;  (5) Due to the sparsity of *cis*-eQTLs in cell types, over 85% genes had only a single valid instrument after LD clumping. This further alleviate the confounding effects of high LD;  (6) To ensure robustness, we only declared a gene as ‘causal’ if it showed consistent effect direction across all used methods and significant in at least one single-SNP method (e.g., SMR or Wald ratio) and one multi-SNP (e.g., IVW, PMR-Egger) method. This strategy helps reduce false positives arising from complex LD patterns, including those in the MHC.  Together, we believe our reported causal genes are unlikely to be influenced by MHC LD structure. |

## REF 1.8 – Add trait heritability in the text

| *Referee Comment* | *As the article focuses on a multitude of traits with differing power, it would be useful to annotate the traits in the text with their heritability.* |
| --- | --- |
| Author Response | Thanks for this suggestion. In the revised manuscript, we have reviewed the original GWAS publications and added the reported SNP-based heritability estimates for each trait where available into **Methods** section. |
| Excerpt 1.8 (Main Text) | **Lines 722-724 (Methods):**  The GWAS summary statistics for brain volume phenotypes were processed by Zhao et al. [158], with a sample size of 19,629 participants of European ancestry from UKB (reported mean heritability: ~40%).  **Lines 725-727 (Methods):**  The GWAS summary statistics for white matter tracts were processed by Zhao et al. [22], with a sample size of 33,292 participants of European ancestry from UKB (reported mean heritability: ~46.3%).  **Lines 685-717 (Methods):**  The sample size for each of the GWAS summary statistics datasets was listed as follows:   - Behavioral-cognitive phenotypes   (1) Automobile speeding propensity (ASP): 404,291 individuals (heritability: 7.9%) [92]  (2) Childhood intelligence (CI): 12,441 individuals (heritability: 22%-46%) [135]  (3) Drinks per week (DPW): 941,280 individuals (heritability: 4%) [136]  (4) Educational attainment (EA): 766,345 individuals (heritability: 12.2%) [137]  (5) General risk tolerance (GRT): 466,571 individuals (heritability: 4.6%) [92]  (6) Intelligence (INT): 269,867 individuals (heritability: 19%) [138]  (7) Neuroticism (NEU): 390,278 individuals (heritability: 10%) [139]  (8) Smoking cessation (SC): 547,219 individuals (heritability: 5%) [136]   - Neurological disorders   (1) Alzheimer's disease (AD): 71,880 cases and 383,378 controls (heritability: 5.5%) [140]  (2) Amyotrophic lateral sclerosis (ALS): 20,806 cases and 59,804 controls [141]  (3) Epilepsy (EPI): 15,212 cases and 29,677 controls (heritability: 9.7%) [142]  (4) Intracerebral hemorrhage (ICH): 1,545 cases and 1,481 controls [143]  (5) Insomnia (INS): 109,548 cases and 277,440 controls (heritability: 7.2%) [144]  (6) Ischemic stroke (IS): 67,162 cases and 454,450 controls [145]  (7) Multiple sclerosis (MS): 47,429 cases and 68,374 controls (heritability: 19.2%) [146]  (8) Parkinson's disease (PD): 33,674 cases and 449,056 controls (heritability: 22%-27%) [147]   - Psychiatric disorders   (1) Attention-deficit/hyperactivity disorder (ADHD): 20,183 cases and 35,191 controls (heritability: 21.6%) [148]  (2) Anxiety disorder (ANX): 25,453 cases and 58,113 controls (heritability: 26%) [149]  (3) Autism spectrum disorder (ASD): 18,381 cases and 27,969 controls (heritability: 11.8%) [150]  (4) Alcohol use disorder (AUD): 11,569 cases and 34,999 controls (heritability: 9.0%) [151]  (5) Bipolar disorder (BIP): 41,917 cases and 371,549 controls (heritability: 18.6%) [152]  (6) Major depressive disorder (MDD): 135,458 cases and 344,901 controls (heritability: 8.7%) [153]  (7) Obsessive-compulsive disorder (OCD): 2,688 cases and 7,037 controls (heritability: 28%) [154]  (8) Post-traumatic stress disorder (PTSD): 9,831 cases and 19,225 controls (heritability: 28%) [155]  (9) Schizophrenia (SCZ): 76,755 cases and 243,649 controls (heritability: 24%) [156]  (10) Tourette syndrome (TS): 4,819 cases and 9,488 controls (heritability: 21%) [157] |

## REF 1.9 – Clarify sample overlap between GWAS datasets

| *Referee Comment* | *For analyses comparing between and within DBs and IDPs, can the authors clarify if there is sample overlap between the compared GWASs? If there is, can overlapping samples be excluded, or accounted for with external replication of findings?* |
| --- | --- |
| Author Response | Thanks for this comment. Because of the widespread use of UK Biobank, all IDP GWASs and half (13 out of 26) of DB GWASs include participants from UK Biobank (we have clarified them in **Lines 864-867**). As our analyses rely on a two-sample MR framework only requiring GWAS summary statistics (i.e. the associations between SNPs and exposure/outcome), individual-level data are not available. Therefore, overlapping samples cannot be directly identified or excluded. This issue of partial sample overlap is a known limitation when using large-scale public GWAS datasets and has been acknowledged in previous Mendelian randomization studies(19, 20). To minimize potential overlap bias from sample overlap:  (1) In all gene expression → IDP/DB MR analyses, we ensured that the eQTL summary statistics (exposure) and the outcome GWAS summary statistics were derived from independent cohorts.  (2) In all bidirectional IDP ↔ DB MR analyses, we excluded all DB GWASs involving UK Biobank samples, retaining a subset of 13 independent DBs (**Lines 864-867**).  We acknowledge that sample overlap between some IDP/DB GWAS datasets might influence the secondary analyses that examine shared causal eGenes across IDPs and/or DBs. This limitation has now been explicitly discussed in the revised manuscript (**Lines 646-652**). |
| Excerpt 1.9 (Main Text) | **Lines 646-652 (Discussion):**  Second, when performing MR between IDPs and DBs, half of the GWASs of DBs were removed due to the inclusion of samples from the popular dataset UK Biobank (UKB) [134], thereby limiting the completeness of inferred putative causality routes among eGenes, IDPs, and DBs (**Fig. 4**). At the same time, shared causal eGenes identified across IDPs and/or DBs might be influenced by unavoidable sample overlap between some GWAS datasets. Future work could aim to collect more fully independent GWAS resources for brain-related traits to improve the robustness and coverage of these causal inferences.  **Lines 864-867 (Methods):**  Because the GWAS of IDPs was conducted using UKB participants, we selected DB GWAS datasets without UKB samples to minimize sample overlap in MR. Then 13 DB GWASs (including CI, EPI, ICH, IS, ALS, MS, OCD, TS, ADHD, AUD, ASD, PTSD, and SCZ) were retained for downstream analysis. |

## REF 1.10 – Clarify TWAS filtering criteria

| *Referee Comment* | *Regarding the TWAS analysis, could the authors clarify the filtering used for gene imputation models? While there is some diversity in the literature, a common threshold is CV R2 >= 0.01 and CV q <= 0.05. While the threshold used as an initial filter was TWAS p <= 0.05, it is important to note how imputable those genes are in the first place.* |
| --- | --- |
| Author Response | Thank you for raising this point. We are happy to clarify the filtering strategy used in our TWAS analyses. In this study, we performed TWAS using MetaXcan (S-PrediXcan)(21). MetaXcan is designed to prioritize genes based on the *p*-value rather than hard thresholds on CV prediction performance. As stated by the developers, the MetaXcan prediction performance *p*-value tends to be smaller when the prediction is more reliable, and *p*-value-based filtering is recommended for gene prioritization(21). For this reason, a separate filtering on CV *R^2^* or CV *q*-value cutoffs is not required  We also want to clarify the role of TWAS in our analytical framework. Usually, before examining whether an exposure causally affects an outcome, it is common to first check whether they show any evidence of association. **In our study,** TWAS serves only as an initial **filtering step to restrict MR analyses to gene-trait pairs with suggestive evidence of association. We do not interpret TWAS hits as stand-alone findings. A relaxed threshold (TWAS *p* ≤ 0.05) helps ensure that potentially relevant pairs are not excluded too early, before being formally evaluated by MR. Using a relaxed filter for pre-selecting candidate associated exposure-outcome pairs before MR analysis is frequently used in relevant literature(18, 22)**. For example, Jing et al. performed MR between brain IDPs and psychiatric traits only for trait pairs with genetic correlation *p* < 0.05**(18)**. Our use of a TWAS *p*-value threshold follows the same rationale. **Overall, TWAS in our analysis was intended only to identify gene-trait pairs that are potentially relevant; causal inference relies entirely on MR, not on the TWAS filter itself.** |

## REF 1.11 – Crossovers in Fig. 4

| *Referee Comment* | *In Figure 4, is there any logic for the Sankey chart crossovers (for example, there is a crossover from "Astrocytes" to "MAPT" and "ZSCAN31"? Or is that just an aesthetic choice? I would remove this if this is purely aesthetic, and if it is not, please make sure it is clearly labeled in the legend.* |
| --- | --- |
| Author Response | Thank you for this suggestion. The crossovers in the original Sankey diagram were automatically produced by the R package ggplot and do not carry any biological or analytical meaning. In the revised manuscript, we have updated this figure to avoid such crossovers for clarity (**Fig. 4**).    **Fig. 4.** **Putative causality routes among cell type-specific causal eGenes, IDPs, and DBs**. **(a)** Putative cell type-specific causal eGene–IDP–DB routes. In each route, the cell type-specific eGene was identified as causal for both IDP and DB and the IDP was identified as causal for the DB. **(b)** Putative cell type-specific causal eGene–DB–IDP routes. In each route, the cell type-specific eGene was identified as causal for both IDP and DB, and the DB was identified as causal for the IDP. |

## REF 1.12 – Legend of Fig. 5a

| *Referee Comment* | *Please clear up the labelling in figure 5A's legend. The shading to indicate prenatal vs postnatal is unintuitive. This could be corrected by applying patterning to indicate prenatal vs postanal, or fully explaining each color in the legend, rather than explaining 2 colors and 2 shades.* |
| --- | --- |
| Author Response | Thank you for this suggestion. We have revised the legend of **Fig. 5a** to improve clarity and remove the previous ambiguity. Specifically, instead of separately indicating color (cell type) and shading (developmental stage), we now explicitly label all four categories in the legend: Prenatal Glia, Postnatal Glia, Prenatal Neuron, and Postnatal Neuron.    **Fig. 5. Spatiotemporal expression for causal eGenes of psychiatric disorders and white matter microstructure phenotypes**. |
| Excerpt 1.12 (Main Text) | **Line 501-513 (Results):**  **Fig. 5. Spatiotemporal expression for causal eGenes of psychiatric disorders and white matter microstructure phenotypes**. **(a)** Expression of causal eGenes for psychiatric disorders in the prenatal stage versus the postnatal stage in glial and neuronal cells. Four groups are explicitly shown: Prenatal Glia (light purple), Postnatal Glia (dark purple), Prenatal Neuron (light yellow), and Postnatal Neuron (dark yellow). **(b)** Expression dynamics of causal eGenes for psychiatric disorders in glial cells. **(c)** Expression dynamics of causal eGenes for psychiatric disorders in neuronal cells. **(d)** Expression of causal eGenes for white matter microstructure phenotypes in the postnatal stage in glial and neuronal cells. For the locally estimated scatterplot smoothing (LOESS) plots, smooth curves are shown with 95% confidence intervals. P4, 13 PCW ≤ Age < 16 PCW; P8, Birth ≤ Age < 6 Months; P12, 12 ≤ Age < 20 Years; TS, tourette syndrome; ANX, anxiety disorder; MDD, major depressive disorder; ASD, autism spectrum disorder; BIP, bipolar disorder; SCZ, schizophrenia. Glia: glial cell, including microglia, astrocytes, oligodendrocytes, and oligodendrocyte precursor cells (OPCs). Neuro: neuronal cell, including inhibitory neurons and excitatory neurons. |

## REF 1.13 – Resolution of Fig. 6

| *Referee Comment* | *The y-axis labeling in figure 6 seems to be low resolution compared to the rest of the text.* |
| --- | --- |
| Author Response | Thank you for this suggestion. We have revised the y-axis labeling in **Fig. 6** and have now submitted high-resolution versions of all figures that meet the journal’s publication standards.    **Fig. 6.** **Coordinated associations** **of brain-associated complex traits based on the expression of causal eGenes. (a)** Heatmap showing the associations within IDPs colored by different groups. **(b)** Heatmap showing the associations between white matter microstructure phenotypes and psychiatric disorders. The associations were evaluated based on the expression correlation (Spearman's correlation coefficient) of the causal eGenes from eight cell types (**Methods**). *, FDR < 0.05; **, FDR < 0.01; ***, FDR < 0.001. |

# *Reviewer #2:*

*This manuscript by Yang et al is a very dense and interesting paper, connecting brain cell-type–specific cis-eQTLs with imaging-derived phenotypes (IDPs) and diseases/behaviors (DBs). The authors report catalogs of causal eGenes and emphasize extensive cell-type specificity (e.g., ~91% of eGene–IDP pairs in only one cell type). The direction is timely and potentially impactful. However, the central claim—cell-type–specific causality—remains insufficiently validated. Replication analyses largely eGene–trait concordance, not cell-type attribution; single-cell QC is under-specified; reliance on an external eQTL map is not audited; and several statistics (e.g., “91.4% unique”) are likely inflated by input data bias, power asymmetries, and thresholding.*

**REF2 – Response to REF2:** We would like to thank you for these insightful comments. Following these suggestions, our manuscript has been improved a lot. Our responses are summarized point-by-point below.

**REF2 – Point to point response to REF2**

## REF 2.1 – Strengthen the replication part by testing cell-type specificity

| *Referee Comment* | *Replication does not test cell-type specificity, while their core claim is about “cell type-specific causal genetic effects”. The current “reproducibility” checks (BrainMeta tissue-level eQTLs, PsychENCODE DE, GWAS Catalog) validate gene-to-trait links, not which cell type is causal. Please consider:*  *• It is concerning when the cell-type specificity cannot be validated. Why not use larger single-cell eQTL resources (e.g., Fujita et al. 2024;* *Mathys et al. 2023)?*  *• In the replication test,* *direction concordance and adjust P-values in the replication stage should be reported.*  *• If the cell-type specificity cannot be confirmed, the newly discovered eGenes and their associated IDPs, DBs are still interesting. But it is important to be certain.* |
| --- | --- |
| Author Response | Thank you for this valuable suggestion. In the revised manuscript, we have strengthened this replication part by (i) validating the cell type specificity of predicted eGenes and (ii) reporting the direction concordance and adjusted *P*-values, as follows.  **(1) Validation of cell type specificity using eQTL summary statistics from *brainSCOPE* and AD-associated DEGs from *Mathys et al. 2023***  The single-cell eQTL data from *Fujita et al., 2024* (https://www.synapse.org/Synapse:syn52363777) are now under access restrictions for Chinese applicants, which prevented their use in this study(23). *Mathys et al., 2023* only reports differential gene expression (DGE) associated with Alzheimer’s disease (AD) pathology, which cannot be used for replication across other brain-associated complex traits (we still used it to test AD-associated causal eGenes as below)(24).  Therefore, we added to incorporate a larger single-cell QTL dataset derived from 388 human prefrontal cortex samples from the *brainSCOPE* resource(25). After matching the cell type definitions with the *Bryois et al* dataset used for our main analysis, we evaluated the six major cell types: astrocytes, excitatory neurons, inhibitory neurons, oligodendrocytes, OPCs, and microglia. The same MR analysis pipeline was applied to *brainSCOPE* eQTL summary statistics to estimate causal effects of cell type-specific gene expression on 149 brain-associated complex traits. An eGene-cell type-trait triple was considered replicated if it reached nominal significance in at least one MR method, and showed consistent effect directions across all MR methods and with the discovery analysis. The results are as follows:   - 41.9% (315/751; *P* < 10^-16^, hypergeometric test) of eGene-cell type-IDP triples were replicated at *P* < 0.05, and 33.7% (253/751; *P* < 10^-16^, hypergeometric test) were replicated at FDR < 0.05 (**S3 Fig**). - 28.7% (84/293; *P* < 10^-16^, hypergeometric test) of eGene-cell type-DB triples were replicated at *P* < 0.05, and 23.5% (69/293; *P* < 10^-16^, hypergeometric test) were replicated at FDR < 0.05 (**S3 Fig**).   We also used the AD-associated DGEs from *Mathys et al. 2023* (https://github.com/mathyslab7/ROSMAP_snRNAseq_PFC/tree/main/Results/Differential_gene_expression_analysis) to validate our predicted AD-associated eGenes. Among the 14 cell type-specific causal eGenes we detected for AD, 12 of them were validated in *Mathys et al. 2023* (**S3 Fig**). Overall, these results support that a substantial proportion of the cell type specificity of our predicted causal eGenes can be independently validated.    **S3 Fig. Replication results for the predicted gene-cell type-IDP and gene-cell type-DB associations.** Reproducibility of eGene-cell type-IDP triples was assessed using cell type-specific eQTL summary statistics from brainSCOPE(25). Reproducibility of eGene-cell type-DB triples was assessed using cell type-specific eQTLs from brainSCOPE(25) and differentially expressed genes from Mathys et al (only AD-associated causal eGenes were tested)(24). Significance of overlap was tested by hypergeometric test.  **(2) Reporting replication results of direction concordance and adjusted *P*-values**  As suggested by the reviewer, we refined our replication criteria. When testing gene-trait associations in *BrainMeta* data, an eGene-trait pair is now considered replicated only if it reaches nominal significance in at least one MR method (with FDR < 0.05 also reported), and showed consistent effect directions across all MR methods and with the discovery analysis. Similarly, in *brainSCOPE*, an eGene-cell type-trait triple is considered replicated only if it meets the same criteria (has been described above). The updated results for *BrainMeta* remain consistent with the original findings:   - 64.9% (430/663; *P* < 10^-16^, hypergeometric test) of eGene-IDP pairs were replicated at *P* < 0.05, and 49.2% (326/663; *P* < 10^-16^, hypergeometric test) were replicated at FDR < 0.05. - 35.7% (87/244; *P* < 10^-16^, hypergeometric test) of the eGene-DB pairs were replicated at *P* < 0.05, and 23% (56/244; *P* < 10^-16^, hypergeometric test) were replicated at FDR < 0.05.   In the revised manuscript, we have now updated the replication results in **Results** (**Lines 164-170, 188-199**), **Methods** (**Lines 832-860**), **S2 Fig**, **S3 Fig**, **S5 Table**, and **S7 Table**.    **S2 Fig.** **Replication results for the predicted gene-IDP and gene-DB associations.** Reproducibility of eGene-IDP pairs was assessed using cortical *cis*-eQTL summary statistics from BrainMeta(12). Reproducibility of eGene-DB pairs was assessed using cortical *cis*-eQTLs from BrainMeta(12), differentially expressed genes from PsychENCODE(13), and gene-trait associations from GWAS Catalog(14). Significance of overlap was tested by hypergeometric test. |
| Excerpt 2.1 (Main Text) | **Lines 164-170 (Results):**  To confirm the reproducibility of the predicted eGene-IDP pairs and eGene-cell type-IDP triples, we repeated MR analyses using cortical eQTL summary statistics from BrainMeta [36] and cell type-specific eQTL summary statistics from brainSCOPE [37], respectively. Among the eGene-IDP pairs, 64.9% (430/663) and 49.2% (326/663) were replicated in BrainMeta at nominal and FDR < 0.05 levels, respectively (both *P* < 10^-16^, hypergeometric test; **S5 Table** and **S2 Fig**). Among the eGene-cell type-IDP triples, 41.9% (315/751) and 33.7% (253/751) were replicated in brainSCOPE at nominal and FDR < 0.05 levels, respectively (both *P* < 10^-16^, hypergeometric test; **S5 Table** and **S3 Fig**).  **Lines 188-199 (Results):**  As we did for the IDPs, we confirmed the reproducibility of eGene-DB pairs using three datasets: the cortical eQTL summary statistics from BrainMeta [36], differentially expressed genes and transcripts from PsychENCODE [29], and documented gene-trait associations from GWAS Catalog [1]. Replication rates were 35.7% (87/244; at nominal significance level) and 23.0% (56/244; at FDR < 0.05 level) in BrainMeta, 29.5% (23/78) in PsychENCODE, and 23.8% (57/240) in the GWAS Catalog (all *P* < 10^-4^, hypergeometric test; **S7 Table** and **S2 Fig**). We further confirmed the reproducibility of eGene-cell type-DB triples using two datasets: the cell type-specific eQTL summary statistics from brainSCOPE [37], and AD-related differentially expressed genes from Mathys et al [42]. We found 41.9% (315/751) and 33.7% (253/751) triples were replicated in brainSCOPE at nominal and FDR < 0.05 levels, respectively (both with *P* < 10^-16^, hypergeometric test; **S7 Table** and **S3 Fig**). Among the 14 AD-associated cell type-specific causal eGenes identified in our analysis, 12 were supported by Mathys et al.  **Lines 832-860 (Methods):**  Replication of predicted associations  We performed replication analysis on the predicted causal associations between eGenes and brain-associated complex traits identified in our MR analysis. To validate the predicted eGene–trait pairs, three external datasets were used. (1) We obtained the *cis*-eQTL summary statistics from the BrainMeta portal (https://yanglab.westlake.edu.cn/data/brainmeta/cis_eqtl/), which was derived using RNA sequencing data of 2,865 brain cortex samples from 2,443 unrelated individuals of European ancestry [36]. The same MR analysis procedure was applied to this data to infer the causal relationships between cortical gene expression and 149 brain-associated complex traits. An eGene–trait pair was considered replicated if it achieved nominal significance in at least one MR method, and showed consistent effect directions across all MR methods and with the discovery analysis. (2) We extracted differentially expressed genes and transcripts (at 5% FDR) in ASD, SCZ and BIP from PsychENCODE [29], which leveraged in vivo gene expression profiles to pinpoint specific alterations in transcriptome-wide isoform levels across the three major psychiatric disorders. (3) We obtained published associations between genes and complex traits from the NHGRI-EBI GWAS Catalog (version 2023-06-09, www.ebi.ac.uk/gwas/) [1].  To validate the predicted eGene–cell type–trait triples, two external datasets were used. (1) We obtained cell type-specific eQTL summary statistics from the brainSCOPE resource (https://brainscope.gersteinlab.org/integrative_files.html), which was generated using single cell-resolution data from 388 adult DLPFC samples [37]. Six major cell types were considered: astrocytes, excitatory neurons, inhibitory neurons, oligodendrocytes, OPCs, and microglia. The same MR analysis procedure was applied to this data to infer the causal relationships between cell type-specific gene expression and 149 brain-associated complex traits. An eGene–cell type–trait triple was considered replicated if it achieved nominal significance in at least one MR method, and showed consistent effect directions across all MR methods and with the discovery analysis. (2) We extracted differentially expressed genes associated with AD pathology in each cell type from Mathys et al. [42]. For each external dataset, we calculated the replication rate as the number of replicated associations divided by the total number of associations tested, and assessed the significance by hypergeometric test. Replication was assessed only for traits that appeared in both the discovery and replication studies. |

## REF 2.2 – Potential bias in estimated cell type specificity

| *Referee Comment* | *Similarly, “91.4% unique to one cell type (for eGene-IDP)” is likely inflated by bias/thresholding, and might be misleading without validating the cell-type specificity. The 91.4% can easily arise from unequal eQTL effect size, underpowered samples,* *instrument availability/strength, QC differences, and per–cell-type thresholding.* |
| --- | --- |
| Author Response | Thank you for this valuable comment. To address this concern, we clarify our rationale and present additional analyses that support the robustness of this observation below:  (1) Both the reported cell-type specificity rates (91.4% for eGene-IDP pairs and 82.1% for eGene-DB pairs) were derived using the same eQTL dataset and analytical pipeline. The used *cis*-eQTL summary statistics were derived from *Bryois et al.*, published in Nature Neuroscience 2022, one of the most comprehensive and rigorously curated resources available(26). All eight cell types were processed through a unified QC pipeline, minimizing artificial differences due to data quality control, per cell-type thresholding, and samples limitation. Additionally, identical instrument selection criteria (*P* < 5×10^-8^) were applied across the analysis, and the Cragg-Donald *F*-statistic was computed to avoid weak instruments bias. Notably, the specificity observed for DBs closely matches a previous report showing that 81.9% of CNS trait-associated eGenes were identified in a single cell type[7], suggesting that this level of specificity is not unexpected.  (2) To test whether the strong cell-type specificity was driven by unequal eQTL signals (e.g., a gene having strong instruments only in one cell type), we performed an additional analysis restricted to genes that have valid instrumental *cis*-eQTLs (*P* < 5×10⁻^8^) in ≥ 2 cell types. Under this restriction, 80.6% (237 out of 294) of predicted eGene-IDP pairs and 69.2% (92 out of 133) of eGene-DB pairs remained specific to exactly a single cell type (**S4 Fig**). This suggests that the high level of cell-type specificity is not simply due to limited eQTL availability or strength, but reflects genuine differences across cell types in their contributions to the traits. We have now incorporated this additional analysis in the revised manuscript (**Lines 173-175, Lines 200-202**).    **S4 Fig. Distribution of cell type specificity for predicted eGene-trait associations.** Bar plots show how many eGene-trait associations are predicted in exactly *k* cell types (x-axis) (restricted to eGenes with valid instrumental *cis*-eQTLs in two or more cell types). The y-axis shows the number of predicted eGene-trait associations that appear in exactly *k* cell types.  (3) We added to validate our findings using an independent eQTL summary statistics from *brainSCOPE* and AD-associated DEGs from *Mathys et al. 2023*, and found a substantial proportion of the cell-type specificity of our predicted causal eGenes can be independently validated (described in **REF 2.1**). Within *brainSCOPE*, 79.0% of eGene-IDP pairs and 78.6% of eGene-DB pairs were specific to a single cell type. Although these rates are slightly lower than those in our discovery dataset, they are consistent in trend and magnitude, supporting the reproducibility and robustness of the cell-type specificity observed in our study.  Together, these analyses suggest that the high proportion of cell type specificity of eGene-trait associations reflects genuine biological differences across cell types rather than technical bias. At the same time, we acknowledge that the estimated specificity rates may still be influenced by confounders. We have now added to discuss this point in the revised manuscript (**Lines 639-646**). |
| Excerpt 2.2 (Main Text) | **Lines 173-175 (Results):**  The high specificity remained after controlling for eQTL availability. Among predicted eGene-IDP pairs where the gene had valid instrumental *cis*-eQTLs in two or more cell types, 80.6% (237 out of 294) were significant in exactly one cell type (**S4 Fig**).  **Lines 200-202 (Results):**  Among predicted eGene-DB pairs where the gene had valid instrumental *cis*-eQTLs in two or more cell types, 69.2% (92 out of 133) were significant in exactly one cell type (**S4 Fig**).  **Lines 639-646 (Discussion):**  First, the lower number of *cis*-eQTLs in endothelial cells and pericytes, compared to other cell types (**S3 Table**) [12], likely limited the number of causal genes identified in these cell types (**Fig. 1b**). This imbalance in eQTL availability may partially affect the observed cell-type specificity patterns of eGene-IDP and eGene-DB associations, particularly for cell types with limited instrument availability. With larger brain single-cell-derived gene expression datasets that have increased sample sizes and finer cell type annotation, we are expected to improve the identification of biologically meaningful genes involved in brain illnesses within these cell types. |

## REF 2.3 – Shared genetic architecture of SCZ and BIP

| *Referee Comment* | *Figure 3 indicates that SCZ and BD have very distinct cell type composition regarding eGenes, and also share a few eGenes. This is contradictory to the common knowledge that SCZ and BD share significant genetics. I suspect the inflated cell-type-specificity is the explanation.* |
| --- | --- |
| Author Response | Thank you for this comment. We would like to clarify that **Fig. 3** shows the cell-type composition of eGenes jointly associated with both SCZ/BIP and IDPs, rather than a direct comparison of SCZ- and BIP-associated eGenes. To address the reviewer’s concern about whether our results align with the well-established genetic overlap between SCZ and BIP, we examined the overlap of cell type-specific causal eGenes between SCZ and BIP. We identified 63 cell type-specific causal eGenes associated with SCZ, 26 associated with BIP, and 12 shared between the two, representing a statistically significant overlap (*P* = 1.27 × 10^-36^, hypergeometric test, **R1 Fig**). Therefore, our result aligns with the known shared genetic basis between SCZ and BIP, while also provides additional insight that distinct subsets of eGenes may contribute to different SCZ/BIP-IDP associations in a cell type-specific manner.    **R1 Fig. Overlap of cell type-specific causal eGenes between SCZ and BIP.** |

## REF 2.4 – Concerns about using external sc-eQTL data

| *Referee Comment* | *Reliance on an external cell-type eQTL resource might be a concern. Your eQTL and sc gene expression data all come entirely from a prior publication. You may need to audit those data and show that their QC/procedures do not manufacture specificity. It is particularly a concern for scRNA-seq data.* *Given high noise and dropout in snRNA-seq, QC will likely affect result consistency, such as Ambient RNA decontamination (e.g., SoupX/DecontX/other): tool/versions/parameters; marker gene uses; missingness/dropout filters: per-cell thresholds (min genes/UMIs, max mito%, doublet score, missing-rate), per-gene thresholds (min expressing-cell fraction within a cell type); noise level in the data, etc.* |
| --- | --- |
| Author Response | Thank you for your comment. In our study, we relied on the comprehensive single-nucleus eQTL resource published by *Bryois et al.* in Nature Neuroscience, which is widely used in the field and has been cited over 250 times(26). Importantly, the original study implemented a highly standardized and stringent QC pipeline to minimize technical noise. Key steps include:  (1)   Per-cell QC and filtering (dropout and noise control) To minimize dropout-related artifacts, each nucleus was required to meet conservative thresholds: - Cells with < 300 detected genes or < 500 UMIs were removed. - Cells with > 5% mitochondrial RNA were excluded. - Doublets were identified and removed using *scDblFinder v1.4.0*. - Outlier nuclei with abnormal splice ratios or mitochondrial proportions were detected by *SampleQC* *v0.4.5* and discarded. - Samples with < 500 high-quality nuclei remaining were excluded from downstream analysis.  (2) Ambient RNA Decontamination Ambient RNA contamination was modeled using R package *DropletUtils* (function *maximumAmbience()*, *v1.14.1*). UMIs from ‘empty droplets’ (< 100 UMIs) were aggregated to estimate background RNA, and genes with an estimated ambient contribution exceeded 10% (a conservative threshold) on average within a cell type were excluded from eQTL mapping.  (3) Data integration and cell‑type annotation Filtered nuclei were integrated using *Conos* *v1.4.6* (50 PCs, res = 8) and *Harmony v0.1.0* (30 PCs, res = 0.8) to minimize batch effects. Cell types were annotated with canonical markers (*SLC17A7* for excitatory neurons, *GAD2* for inhibitory neurons, *AQP4* for astrocytes, *MOG* for oligodendrocytes, *C1QA* for microglia, *CLDN5* for endothelial cells, *RGS5* for pericytes). Ambiguous or mixed clusters were excluded. Pseudo-bulk matrices were then generated per individual and normalized using TMM, reducing noise from sparse single-nucleus counts.  (4)  Per‑gene QC and dropout filtering Only genes with average expression ≥ 1 count per million within a given cell type were retained. Genes dominated by ambient RNA or expressed in less than 10 individuals per cell type were removed.  (5) Genotype QC Standard genotype QC was applied, including removal of SNPs with imputation INFO < 0.4, MAF < 5%, or Hardy-Weinberg P < 1×10^-6^; removal of individuals with > 2% missing genotypes or relatedness; verifying consistency between genotype and RNA-seq data using *MBV* (*QTLtools v1.3.1*).  (6) *cis*-eQTL mapping *cis*‑eQTLs (±1 Mb from TSS) were mapped with *FastQTL v2*.0 using 70 expression PCs, three genotype PCs, and technical covariates as regressors.  (7) *cis*-eQTL validation *Bryois et al.* further demonstrated that these *cis*-eQTLs are robust and biologically meaningful, as evidenced by: 1) strong replication in external bulk eQTL datasets, 2) enrichment near transcription start sites, 3) enrichment in cell-type-specific epigenomic marks, and 4) display expected null *P*‑value distributions under permutation.  Importantly, our study adopts a two-sample MR design, which inherently relies on independent, large summary-level datasets for the exposure and outcome. This is a widely used and standard practice in MR studies, as it leverages existing datasets to maximize statistical power and reproducibility. Instead of generating new eQTL data ourselves, we focused on integrating existing well-curated, high-quality resources and applying multiple complementary methods to make causal inferences between gene expression and a broad range of complex traits across cell types. To further reduce the risk that our findings are driven by artifacts in any single dataset, we additionally validated key results using five independent resources (*brainSCOPE*, *Mathys et al. 2023*, *BrainMeta*, *PsychENCODE*, and *GWAS Catalog*), which showed consistent patterns of association (also illustrated in **REF 2.1**).  Together, the multi-layered QC procedures used in *Bryois et al.* dataset at both cell and gene levels, combined with our carefully designed analysis pipeline and validation, help ensure that the observed cell type-specific causal signals are biologically meaningful rather than noise from inadequate QC. |

## REF 2.5 – Systematically map DB-IDP relationships through cell-type and SNPs

### REF 2.5.1 – Trait clustering based on cell type-resolved causal gene sharing

| *Referee Comment* | *Systematically map DB–IDP relationships* *through cell-type and SNPs. Current sharing/overlap is primarily at the gene level. To deliver the promise on “bridging cell type/region/behavior” additional analyses might be useful.*  *• Build a phenotype × phenotype similarity matrix using cell-type–resolved overlap (Jaccard/overlap, optionally IV-weighted) of causal eGenes within cell types, then aggregate across cell types.* *Cluster phenotypes; report cluster stability (bootstrap), enrichment of trait classes* *within clusters, and which cell types drive cohesion.* |
| --- | --- |
| Author Response | Thank you for the constructive suggestion, and we really appreciate it. Following this advice, we extended our analysis to investigate the cellular basis underlying the trait × trait similarity matrix derived from cell type-resolved overlap of causal eGenes. (1) We first constructed cell type-specific trait × trait Jaccard similarity matrices using shared causal eGenes in each of the cell types. Averaging these matrices across cell types yielded an aggregate trait similarity matrix (**S11 Fig**). To evaluate which cell types mostly drive this global similarity pattern, we computed the correlation between each cell type-specific matrix and the aggregate matrix. Oligodendrocytes, excitatory neurons, and astrocytes emerged as the top contributors (**S12 Fig**), in line with their higher numbers of trait-associated causal eGenes (**Fig. 1b-c**). This supports their central roles in shaping shared regulatory mechanisms across brain-associated complex traits.  **S11 Fig. Hierarchical clustering of the aggregate trait similarity matrix derived from shared cell type-specific causal eGenes.** For each of the cell types, we constructed a trait × trait Jaccard similarity matrix based on shared causal eGenes. These were averaged to generate an aggregate similarity matrix, followed by hierarchical clustering. The resulting 12 trait clusters (C1 ~ C12) are annotated on the heatmap.  **S12 Fig. Cell type contributions to the aggregate trait similarity matrix.** Bar plot showing the Pearson correlation between each cell type-specific trait similarity matrix and the aggregate similarity matrix. Higher correlations indicate greater contributions of the corresponding cell type to the global trait similarity pattern. (2) We then performed hierarchical clustering for the aggregate similarity matrix using R package *pvclust* (version 2.2, 10,000 bootstraps)(27). We identified 12 robust trait clusters with approximately unbiased *P*-value ≥ 0.95 (**S11 Fig, S13 Fig** and **S10 Table**). Notably, eight clusters (C2 ~ C9 and C12) contained both IDPs and DBs. Clusters C6 and C7 were the most heterogeneous, each spanning all five trait groups. To identify which cell types drive the within-cluster similarity pattern, we computed, for each cluster, the correlation between the aggregate similarity matrix and each cell type-specific similarity matrix restricted to that cluster. In both C6 and C7, Inhibitory neurons emerged as the top contributor, highlighting a key role in mediating shared molecular regulation across diverse phenotypic categories (**S13 Fig**). We further assessed the enrichment of trait groups within each cluster using hypergeometric tests **(S13 Fig)**. Cluster C4 showed significant enrichment for white matter microstructure phenotypes (FDR = 0.0014) and nominal enrichment for psychiatric disorders (*P* = 0.03). Interestingly, microglia contributed most strongly to similarity within this cluster, pointing to cell type-specific regulatory mechanisms underlying this cluster.  **S13 Fig. Trait group enrichment and dominant cell type per cluster.** Bar plot showing the enrichment ratio of each trait group within the 12 identified trait clusters (hypergeometric tests). Significance is indicated by asterisks (*: FDR < 0.05, #: *P* < 0.05). The dominant cell type contributing to each cluster's similarity structure (having the highest correlation with the aggregate matrix within that cluster) is shown on the left. **S10 Table. Trait composition of each of the 12 clusters derived from hierarchical clustering of the aggregate trait similarity matrix.** BRV: Brain regional volume; WMMP: White matter microstructure phenotype; BCP: Behavioral-cognitive phenotype; NEUD: Neurological disorder; PSYD: Psychiatric disorder. **Cluster** \| **Trait** \| **Group**; C1 \| Right amygdala \| BRV; C1 \| CSF \| BRV; C2 \| Right thalamus proper \| BRV; C2 \| CST_FA \| WMM; C2 \| Left posterior cingulate \| BRV; C3 \| CGC_FA \| WMM; C3 \| SFO_FA \| WMM; C3 \| EC_FA \| WMM; C3 \| ALIC_FA \| WMM; C3 \| Right accumbens area \| BRV; C3 \| Left accumbens area \| BRV; C3 \| Right superior frontal \| BRV; C3 \| Right pars orbitalis \| BRV; C3 \| WVol \| BRV; C3 \| Left pallidum \| BRV; C3 \| BVol \| BRV; C3 \| Right pallidum \| BRV; C3 \| IFO_FA \| WMM; C3 \| Left amygdala \| BRV; C3 \| CGH_FA \| WMM; C3 \| FX_FA \| WMM; C3 \| Left fusiform \| BRV; C3 \| Left entorhinal \| BRV; C3 \| Right basal forebrain \| BRV; C3 \| Right cuneus \| BRV; C3 \| Right putamen \| BRV; C3 \| Left putamen \| BRV; C3 \| Right lateral orbitofrontal \| BRV; C3 \| Left thalamus proper \| BRV; C3 \| Right postcentral \| BRV; C3 \| Left superior parietal \| BRV; C3 \| Left basal forebrain \| BRV; C3 \| Right parahippocampal \| BRV; C3 \| Left lingual \| BRV; C3 \| Right entorhinal \| BRV; C3 \| Right fusiform \| BRV; C3 \| Right lingual \| BRV; C3 \| Left pars triangularis \| BRV; C3 \| Left supramarginal \| BRV; C3 \| Optic chiasm \| BRV; C3 \| Left postcentral \| BRV; C3 \| Left parahippocampal \| BRV; C3 \| Left vessel \| BRV; C3 \| Right posterior cingulate \| BRV; C3 \| DPW \| BCP; C4 \| SS_FA \| WMM; C4 \| ACR_FA \| WMM; C4 \| BCC_FA \| WMM; C4 \| PLIC_FA \| WMM; C4 \| Left superior frontal \| BRV; C4 \| Average_FA \| WMM; C4 \| Left transverse temporal \| BRV; C4 \| SCC_FA \| WMM; C4 \| SLF_FA \| WMM; C4 \| PTR_FA \| WMM; C4 \| GCC_FA \| WMM; C4 \| Left pars opercularis \| BRV; C4 \| Right medial orbitofrontal \| BRV; C4 \| RLIC_FA \| WMM; C4 \| Left inferior temporal \| BRV; C4 \| Right supramarginal \| BRV; C4 \| BIP \| PSYD; C4 \| SCZ \| PSYD; C4 \| ANX \| PSYD; C4 \| MDD \| PSYD; C5 \| Left precentral \| BRV; C5 \| Right middle temporal \| BRV; C5 \| Right precentral \| BRV; C5 \| Right precuneus \| BRV; C5 \| Left inferior parietal \| BRV; C5 \| AD \| NEUD; C6 \| SCR_FA \| WMM; C6 \| FXST_FA \| WMM; C6 \| Right cerebellum exterior \| BRV; C6 \| Right ventral DC \| BRV; C6 \| Right cerebellum white matter \| BRV; C6 \| PCR_FA \| WMM; C6 \| Right rostral middle frontal \| BRV; C6 \| Brain stem \| BRV; C6 \| Left ventral DC \| BRV; C6 \| UNC_FA \| WMM; C6 \| Right hippocampus \| BRV; C6 \| Left hippocampus \| BRV; C6 \| Left middle temporal \| BRV; C6 \| GVol \| BRV; C6 \| Left cerebellum white matter \| BRV; C6 \| Left isthmus cingulate \| BRV; C6 \| Left superior temporal \| BRV; C6 \| Cerebellar vermal lobules VI VII \| BRV; C6 \| Left cerebellum exterior \| BRV; C6 \| EPI \| NEUD; C6 \| GRT \| BCP; C6 \| ASP \| BCP; C6 \| PTSD \| PSYD; C6 \| ADHD \| PSYD; C7 \| X3rd ventricle \| BRV; C7 \| Right lateral ventricle \| BRV; C7 \| Left lateral ventricle \| BRV; C7 \| Right caudal anterior cingulate \| BRV; C7 \| Right pericalcarine \| BRV; C7 \| Right inferior lateral ventricle \| BRV; C7 \| Left pericalcarine \| BRV; C7 \| Right transverse temporal \| BRV; C7 \| Left inferior lateral ventricle \| BRV; C7 \| NEU \| BCP; C7 \| ICH \| NEUD; C7 \| IS \| NEUD; C7 \| ASD \| PSYD; C7 \| OCD \| PSYD; C7 \| TS \| PSYD; C8 \| Left caudate \| BRV; C8 \| Right caudate \| BRV; C8 \| X4th ventricle \| BRV; C8 \| MS \| NEUD; C9 \| Right isthmus cingulate \| BRV; C9 \| EA \| BCP; C9 \| SC \| BCP; C9 \| INT \| BCP; C10 \| Cerebellar vermal lobules I \| BRV; C10 \| Left insula \| BRV; C10 \| Right insula \| BRV; C10 \| Right paracentral \| BRV; C10 \| Left caudal anterior cingulate \| BRV; C10 \| Left caudal middle frontal \| BRV; C11 \| Left paracentral \| BRV; C11 \| Left cuneus \| BRV; C12 \| Left pars orbitalis \| BRV; C12 \| Right rostral anterior cingulate \| BRV; C12 \| Right vessel \| BRV; C12 \| Right caudal middle frontal \| BRV; C12 \| Right superior parietal \| BRV; C12 \| ALS \| NEUD; C12 \| PD \| NEUD; C12 \| AUD \| PSYD; C12 \| CI \| BCP In the revised manuscript, we have incorporated this new analysis into Results (**Lines 385-397**) and **S1 Text**. |
| Excerpt 2.5.1 (Main Text and supplementary Text) | **Lines 385-397 (Results):**  **Cell type-informed clustering of traits reveals biologically meaningful patterns**  To explore the common regulatory basis across traits, we computed a trait × trait Jaccard similarity matrix based on shared cell type-specific causal eGenes (**S11 Fig;** also see **S1 Text**). Oligodendrocytes, excitatory neurons, and astrocytes emerged as the top contributors to the global trait similarity pattern (**S12 Fig**), in line with their higher numbers of trait-associated causal eGenes (**Fig. 1b-c**). Hierarchical clustering of the similarity matrix revealed 12 robust trait clusters (**S11 Fig** and **S10 Table**). Notably, eight clusters (C2 ~ C9 and C12) contained traits from both IDP and DB classes (**S13 Fig**). Clusters C6 and C7 were the most heterogeneous, each spanning all five trait groups. In both C6 and C7, inhibitory neurons emerged as the top contributors, highlighting a key role in mediating shared molecular regulation across diverse phenotypic categories. Cluster C4 exhibited significant enrichment for white matter microstructure phenotypes (FDR = 0.0014, hypergeometric tests) and a nominal enrichment for psychiatric disorders (*P* = 0.03, hypergeometric tests), with microglia emerging as the dominant cell type, pointing to cell type-specific regulatory mechanisms underlying this cluster.  **S1 Text**:  S1 Text. Trait similarity matrix construction and clustering analysis  For each brain cell type, we constructed a trait × trait Jaccard similarity matrix based on the predicted causal eGenes. The Jaccard index for each trait pair was calculated as the number of their shared causal eGenes divided by the number of their causal eGenes union. These cell type-specific matrices were then averaged to produce an aggregate similarity matrix representing global trait similarity across cell types. To evaluate which cell types mostly drive this global similarity pattern, we computed the Pearson correlation between each cell type-specific matrix and the aggregate matrix.  Hierarchical clustering was next performed on the aggregate similarity matrix using R package *pvclust* (version 2.2) with average linkage and correlation distance^1^. Cluster stability was assessed through 10,000 bootstrap replicates, and clusters with approximately unbiased *P*-values ≥ 0.95 were considered significant. To evaluate which cell types mostly drive within-cluster similarity pattern, we calculated the Pearson correlation between each cell type-specific matrix and the aggregate matrix and restricted to the traits within a given cluster. We further performed trait group enrichment within clusters using hypergeometric tests and corrected for multiple testing using the false discovery rate (FDR). |

### REF 2.5.2 – SNP-level extension

| *Referee Comment* | *Systematically map DB–IDP relationships through cell-type and SNPs. Current sharing/overlap is primarily at the gene level. To deliver the promise on “bridging cell type/region/behavior” additional analyses might be useful.*  *• Variant-resolved extension is recommended: fine-map eQTLs per cell type and GWAS loci; perform variant-level colocalization; repeat the clustering using shared functional SNPs (more specific than genes).* *Annotate SNPs to cell-type/region enhancers and TF motifs to provide a mechanistic chain (SNP→element→gene→cell type→brain region→phenotype). Include a short list of sentinel variants per cluster suitable for MPRA/CRISPRi/a follow-up.* |
| --- | --- |
| Author Response | Thank you for your insightful suggestion. While variant-level analyses indeed offer important mechanistic depth, they fall outside the main focus of our current study. Our goal is to identify causal relationships between cell type-specific gene expression and a wide range of brain-related complex traits using MR. In this context, SNPs serve merely as instrumental variables that meet the core MR assumptions (Relevance, Independence, and Exclusion Restriction(28)) to support causal inference from gene expression to traits, rather than as biological drivers which colocalization assumes. Moreover, introducing such analyses across hundreds of traits and multiple cell types could introduce additional complexity and make the causality harder to interpret. Therefore, we believe that incorporating a full variant-level layer across all traits and genes would conflate distinct causal mechanisms and blur rather than clarify the gene-level specificity we aim to uncover.  To address the reviewer’s suggestion in a focused and interpretable manner, we performed a targeted variant-level analysis on 11 representative cell type-specific eGene → Trait1 (IDP or DB) → Trait2 (DB or IDP) routes highlighted in our main results (**Fig. 4** and **S12 Table**). Specifically, we reconstructed the full chain routes from SNP to brain-associated complex traits through the following steps:  **(1) Trait pair selection via clustering.** We selected Trait1–Trait2 pairs that co-clustered in our aggregate trait similarity matrix (**S11 Fig**), indicating they are regulated by a common set of cell type-specific eGenes. Notably, nearly all selected pairs in the 11 selected routes (e.g., GCC_FA–SCZ, ACR_FA–SCZ, SCZ–Right medial orbitofrontal) resided in Cluster C4, supporting their convergence at the gene-cell type level.  **(2) Fine-mapping and colocalization.** For each cell type-specific eGene-Trait1 combination in the selected routes, we first fine-mapped both the corresponding eQTLs and Trait1 GWAS loci using SuSiE algorithm (R package *susieR*, version 0.14.2), which allows modeling multiple causal variants per region(29). Next, we applied the function *coloc.susie* from R package *coloc* (version 6.0.0) to perform colocalization using the posterior distributions from fine-mapping(30). Loci with posterior probability (PP.H4) exceeding 0.8 were considered colocalized. From these, SNPs with individual-level posterior probability (PP.H4.snp) exceeding 0.01 were retained as putative functional variants for further annotation. This yielded a set of SNP → eGene → cell type → Trait1 combinations likely to reflect mechanistic links.  **(3) Annotation of functional SNPs.** We annotated these colocalized SNPs to regulatory features including chromatin state, DNAse hypersensitivity regions, and transcription factor motif disruptions using *HaploReg* (version 4.2)(31). This allowed us to link SNPs to potential regulatory elements and complete the chain: SNP (regulatory elements annotated) → cell type-specific eGene → Trait1 → Trait2.  Here is a representative example: rs3130455 (annotated to promoter histone marks in 24 tissues, DNAse in 53 tissues, and 12 altered motifs) → *CCHCR1* in excitatory neurons → SCZ → PTR_FA. The SNP rs3130455 colocalized between the expression of *CCHCR1* in excitatory neurons and SCZ (PP.H4 = 0.85, PP.H4.snp = 0.15). Functional annotation showed that rs3130455 is marked by active promoter chromatin states (ChromHMM state 1_TssA) and exhibits high DNAse I hypersensitivity across several neural lineages, including H1/H9-derived neuronal progenitor cells and fetal brain tissue. Furthermore, rs3130455 disrupts multiple transcription factor motifs (e.g. *Klf4* and *Klf7*) implicated in neurodevelopmental regulation. This integrated evidence suggests a potential mechanism: rs3130455 modulates *CCHCR1* expression via its promoter in excitatory neurons, thereby linking the causal relationship from SCZ risk to alterations in the microstructure of PTR. Other examples include rs55938136 → *MAPT* in astrocytes → GCC → SCZ, where the intronic SNP rs55938136 colocalized with *MAPT* expression in astrocytes and the FA value of GCC (PP.H4 = 1, PP.H4.snp = 1).  We have included a complete list of 56 SNP – regulatory elements – eGene – cell type – Trait1 – Trait2 chains in **S13 Table** and updated the Results (**Lines 468-481**) and Supplementary Text (**S2 Text**) accordingly.  **S13 Table. Putative SNP–cell type-specific eGene–Trait1 (IDP or DB)–Trait2 (DB or IDP) chains.** In each chain, Trait1 and Trait2 were co-clustered in the aggregate trait similarity matrix, the eGene was inferred as causal for both traits, Trait1 was inferred as causal for Trait2, and the SNP was colocalized with both the eGene and Trait1 and annotated to regulatory elements.   |
| Excerpt 2.5.2 (Main Text and Supplementary Text) | **Lines 468-481 (Results):**  To further trace potential regulatory variants underlying these 11 causality routes shown in **Fig. 4**, we conducted a focused SNP-level fine-mapping, colocalization, and regulatory annotation analysis (see **S2 Text** for details). We reconstructed 56 SNP–cell type-specific causal eGene–Trait1 (DB or IDP)–Trait2 (IDP or DB) chains. For example, one such chain is rs3130455–*CCHCR1* in excitatory neurons–SCZ–PTR (effect direction: +, +, +), where rs3130455 is marked by active promoter chromatin states (ChromHMM 1_TssA) across neural lineages and disrupts transcription factor motifs implicated in neurodevelopment such as *Klf4* and *Klf7*. It suggests that increased SNP dosage may elevate the expression level of *CCHCR1* in excitatory neurons, thereby elevating SCZ risk, and further enhancing white matter connectivity in PTR. Another example is rs55938136–*MAPT* in astrocytes–genu of corpus callosum (GCC)–SCZ (effect direction: +, –, +), where the intronic SNP rs55938136 showed perfect colocalization with *MAPT* expression in astrocytes and the FA value of GCC (PP.H4 = 1, PP.H4.snp = 1). This indicates that increased SNP dosage may raise *MAPT* expression in astrocytes, and reduce structural connectivity of GCC, and consequently increase SCZ susceptibility [113, 114]. The full list of chains is summarized in **S13 Table**.  **S2 Text:**  SNP-level colocalization and regulatory annotation  We performed a targeted variant-level analysis on 11 cell type-specific eGene–Trait1 (IDP or DB)–Trait2 (DB or IDP) routes highlighted in our main results (**Fig. 4** and **S12 Table**). Specifically, we reconstructed the full chain routes from SNP to brain-associated complex traits through the following steps:  (1) Trait pair filtering via clustering. We first selected Trait1–Trait2 pairs that co-clustered in our aggregate trait similarity matrix (**S11 Fig**), indicating they are regulated by a common set of cell type-specific eGenes. Notably, nearly all selected pairs in the 11 selected routes (e.g., GCC_FA–SCZ, ACR_FA–SCZ, SCZ–Right medial orbitofrontal) resided in the same cluster (Cluster C4), supporting their convergence at the gene-cell type level.  (2) Fine-mapping and colocalization. For each cell type-specific eGene–Trait1 combination in the selected routes, we first fine-mapped both the corresponding eQTLs and Trait1 GWAS loci using the SuSiE algorithm (R package *susieR*, version 0.14.2), which allows modeling multiple causal variants per region^2^. Next, we applied the function *coloc.susie* from R package *coloc* (version 6.0.0) to perform colocalization using the posterior distributions from fine-mapping^3^. Loci with posterior probability (PP.H4) exceeding 0.8 were considered colocalized. From these, SNPs with individual-level posterior probability (PP.H4.snp) larger than 0.01 were retained as putative functional variants for further annotation. This yielded a set of SNP–eGene–cell type–Trait1 combinations likely to reflect mechanistic links.  (3) Annotation of functional SNPs. We annotated these colocalized SNPs to regulatory features including chromatin states, DNAse hypersensitivity, and transcription factor motif disruptions using *HaploReg* (version 4.2)^4^ This allowed us to link SNPs to potential regulatory elements and complete the chain: SNP (regulatory elements annotated)–cell type-specific eGene–Trait1–Trait2. |

## REF 2.6 – Clarification on bidirectional IDP–DB effects

| *Referee Comment* | *Bidirectional IDP↔DB effects could be quantified and classified. Don’t drop it. Pairs showing bidirectional MR were excluded “for clarity” in current manuscript, but they are informative.* |
| --- | --- |
| Author Response | Thank you for pointing this out. In our initial manuscript, we stated that IDP-DB pairs showing bidirectional causality were excluded because reverse causation can lead to unreliable MR results even under careful study design(28). In fact, many previous studies have chosen to remove such bidirectional findings to minimize interpretational ambiguity(19, 32). As a result, our analysis pipeline was set to automatically filter out bidirectional pairs using the following R code:  *bidirect = intersect(path1$id, path2$id) path1 = path1[-which(path1$id %in%* *bidirect), ] path2 = path2[-which(path2$id %in% bidirect), ]*  However, after double-checking the current results, we found no IDP-DB pair actually showing bidirectional causality. As a result, no results were excluded. To avoid confusion, we have removed this misleading sentence ‘Additionally, IDP-DB pairs showing bidirectional causality were excluded from the results to ensure clarity in determining directional influences’ from the revised manuscript. We thank the reviewer for the helpful comment. |

## REF 2.7 – Overinterpretation about genetic constraint of shared causal eGenes

| *Referee Comment* | *“Genetic constraint of shared causal eGenes” is confounded as currently framed. The claim that DB-implicated genes are “more conserved” than IDP-implicated genes is difficult to interpret due to class breadth, power asymmetry, and eGene detectability biases (highly constrained genes often lack strong common cis-eQTLs).* |
| --- | --- |
| Author Response | Thank you for the suggestion. We agree that comparing genetic constraint between DB- and IDP-implicated genes may be confounded by multiple factors including class heterogeneity, differences in power, and biases in eQTL detectability. To avoid overinterpretation, we have removed the relevant conclusive statement in the revised manuscript (**Lines 276-284**). |
| Excerpt 2.7 (Main Text) | **Lines 276-284 (Results):**  Next, we explored the genetic constraint and enriched biological functions of the shared causal eGenes between pairs of traits within the same trait class (middle panel in **Fig. 2b**). We observed that the causal eGenes shared between the neurological disorder and psychiatric disorder groups showed the highest pLI scores (*μ* = 0.409, *σ^2^* = 0.235), in contrast to those shared between brain regional volume and white matter microstructure groups, which presented the lowest scores (*μ* = 0.153, *σ^2^* = 0.125). Consistently, the gene-set enrichment analysis revealed that the causal eGenes shared between the neurological disorder and psychiatric disorder groups were involved in biological functions, such as cell maintenance and cell signaling (e.g., plasma membrane organization; **Fig. 2c** and **S9 Table**), which have been linked to the etiology of neuropsychiatric disorders [59, 60]. |

## REF 2.8 – Misused phrase of “mutually causal”

| *Referee Comment* | *“Mutually causal” might be a misused phrase where pleiotropy/overlap across traits might be intended. Reserve “mutual/bidirectional” for true A↔B MR that passes predefined criteria.* |
| --- | --- |
| Author Response | Thank you for the suggestion. In light of your comment, we have changed the phrase “were found to be mutually causal across” to “showed pleiotropic causal associations across” which is more suitable (**Lines 228-232**). |
| Excerpt 2.8 (Main Text) | **Lines 228-232 (Results):**  For the DB class, four causal eGenes — *DDHD2* in excitatory neurons, *MAP2K5* in inhibitory neurons, *XKR6* in inhibitory neurons, and *LIN28B* in excitatory neurons — showed pleiotropic causal associations across neurological disorder, behavioral-cognitive phenotype and psychiatric disorder groups (**S8 Table**). |

## REF 2.9 – Explain inconsistency with previous literature

| *Referee Comment* | *Lines 180–190: The authors report* *excitatory and inhibitory neurons as predominant cell types, even for AD. This contrasts with Mathys et al. 2023, which emphasized microglia. How do the authors explain this inconsistency?* |
| --- | --- |
| Author Response | Thank you for the comment. Our study focuses on identifying genes whose expression levels causally affect AD risk using MR, whereas *Mathys et al. 2023.* focused on identifying DGEs associated with AD(24). DGE reflects correlation and potential downstream effects of disease progression, while MR seeks to infer causal directionality from gene expression to disease risk. Therefore, the two studies address different biological questions. Notably, microglia have shown strong transcriptional changes in response to AD pathology. A recent study has shown that microglia were the only cell type where most gene regulatory differences were driven by AD disease status, suggesting that microglial changes may be a consequence rather than a driver of AD risk(15). This could explain why our MR analysis did not prioritize microglia as a key causal cell type. We also compared the 14 AD-associated cell type-specific causal eGenes identified in our study with significant DEGs from *Mathys et al. 2023*, and found 12 of them were overlapped (**R1 Table**, also described in **REF 2.1**). Moreover, *Mathys et al. 2023* also highlighted the involvement of excitatory neurons, oligodendrocytes and inhibitory neurons in AD, which aligns with the cell types highlighted in our findings. **R1 Table. Replication results for the predicted cell type-specific causal eGenes for AD using differentially expressed genes from *Mathys et al. 2023*.** **Cell type-specific causal eGenes detected for AD in our study** \| \| **Replicated in *Mathys et al. 2023*** \|; **Cell type** \| **eGene** \| \|; Pericytes \| *DNAH11* \| TRUE \|; Excitatory neurons \| *MSH3* \| TRUE \|; Oligodendrocytes \| *NUP43* \| FALSE \|; Oligodendrocytes \| *ADAM10* \| TRUE \|; Excitatory neurons \| *SYT14* \| TRUE \|; Excitatory neurons \| *ICA1L* \| TRUE \|; Astrocytes \| *STXBP6* \| TRUE \|; Excitatory neurons \| *RGS14* \| TRUE \|; Oligodendrocytes \| *CSGALNACT2* \| TRUE \|; OPCs \| *CSGALNACT2* \| FALSE \|; Inhibitory neurons \| *C17orf97* \| TRUE \|; Excitatory neurons \| *C17orf97* \| TRUE \|; Oligodendrocytes \| *CR1* \| TRUE \|; Excitatory neurons \| *ZSCAN31* \| TRUE \| |
| Excerpt 2.9 (Main Text) | **Lines 197-199 (Results):**  Among the 14 AD-associated cell type-specific causal eGenes identified in our analysis, 12 were supported by Mathys et al. |

## REF 2.10 – Explanation of the expression of causal genes

| *Referee Comment* | *The authors report higher expression of causal eGenes in glial cells, yet most causal genes were identified in neurons. How can this be reconciled?* |
| --- | --- |
| Author Response | Thank you for the comment. The apparent discrepancy arises from the distinction between genetic regulatory causality and steady-state transcriptional abundance. In our study, cell type-specific causal eGenes were identified using a single-cell Mendelian randomization framework that leverages cis-eQTLs as genetic instruments. Therefore, a gene being “identified” in a given cell type reflects the presence of significant genetic regulatory effects on its expression within that cell type, rather than its absolute expression level.  In contrast, the analysis presented in **Fig. 5** characterizes the steady-state expression patterns of these causal eGenes using independent single-cell RNA-seq data. Higher expression levels in glial cells do not necessarily imply stronger or more detectable genetic regulation in glia. Indeed, regulatory effect size, genetic variance, and instrument strength can differ substantially across cell types, independent of baseline transcriptional abundance.  Therefore, our findings suggest that genetic liability for brain-associated traits may be preferentially mediated through neuronal regulatory architecture, while the transcriptional output of these genes may be higher in glial populations. We have clarified this distinction in the revised manuscript to avoid potential confusion. |
| Excerpt 2.10 (Main Text) | **Lines 523-529 (Results):**  Importantly, the spatiotemporal expression analyses provide an additional biological layer to complement the genetically inferred causal relationships. While the identification of cell type-specific causal eGenes reflects cis-eQTL–driven regulatory architecture, their developmental and cell type-specific transcriptional dynamics offer independent evidence of biological plausibility. The observed enrichment of causal eGene expression in glial cells and their distinct prenatal-postnatal trajectories suggest coordinated regulatory and transcriptional mechanisms underlying brain-associated complex traits. |

## REF 2.11 – Potential underrepresentation of developmental progenitor populations

| *Referee Comment* | *Figure 5: Progenitor cells are not included in the prenatal data. Would that be a missed opportunity?* |
| --- | --- |
| Author Response | Thank you for the comment. Our spatiotemporal expression analyses were specifically performed for the causal eGenes identified through the single-cell Mendelian randomization framework. Since progenitor cells were not included as a distinct cell type in the cis-eQTL dataset used for MR inference, no progenitor cell-specific causal eGenes were identified. Consequently, progenitor cells were not included in the downstream expression analyses, which were designed to evaluate the transcriptional patterns of MR-inferred eGenes in a cell type-matched manner.  Therefore, this does not represent a missed opportunity but rather reflects the methodological scope of our study. We agree that incorporating progenitor cell-specific eQTL resources in the future would be valuable for extending this framework to early developmental contexts. |

## REF 2.12 – Explain inconsistent chains

| *Referee Comment* | *For key gene–trait–cell-type triplets, focus on sign-consistency chains (eQTL allele → expression → MR effect on trait), and explain inconsistent chains.* |
| --- | --- |
| Author Response | Thank you for the suggestion. Since our goal is to robustly infer causal relationships between gene expression and traits, we employed four complementary MR methods, each using distinct instrumental SNP sets. As a result, the number and identity of instrumental SNPs vary greatly across genes (some having only a few instruments and others having hundreds). Importantly, SNPs included in MR only serve as instruments rather than biological drivers. Since these MR methods aggregate signals across all selected SNPs for a given gene, the causal inference is made at the gene level, making it challenging to interpret directly from individual eQTL alleles.  To better illustrate putative regulatory chains, we focused on previously derived 56 SNP → cell type-specific eGene → Trait1 (DB or IDP) → Trait2 (IDP or DB) chains shown in **S12 Table** (also illustrated in **REF 2.5.2**), where the SNP is colocalized with both the eGene and Trait1. For each chain, we further examined the effect direction across the three links: 1) the effect of SNP on gene expression (*cis*-eQTL effect), 2) the MR causal estimate of the eGene on Trait1, and 3) the MR causal estimate of Trait1 on Trait2. For example, the chain rs3130455 → *CCHCR1* in excitatory neurons → SCZ →posterior thalamic radiation (PTR) shows consistent positive effects (+ → + → +), suggesting that increased dosage of the SNP may upregulate *CCHCR1* expression in excitatory neurons, thereby elevating SCZ risk, and ultimately enhancing white matter connectivity in PTR. Another chain rs55938136 → *MAPT* in astrocytes → GCC → SCZ shows a + → – → + pattern, where increased SNP dosage raises *MAPT* expression in astrocytes, which reduces structural connectivity of GCC, and in turn increases SCZ susceptibility(33, 34). Although the signs differ across the path, this still forms a biologically plausible indirect causal path.  In the revised manuscript, we summarized the directionality of all 56 such chains in **S13 Table** and have incorporated this information into the Results section **(Lines 468-481)**. |
| Excerpt 2.12 (Main Text) | **Lines 468-481 (Results):**  To further trace potential regulatory variants underlying these 11 causality routes shown in **Fig. 4**, we conducted a focused SNP-level fine-mapping, colocalization, and regulatory annotation analysis (see **S2 Text** for details). We reconstructed 56 SNP–cell type-specific causal eGene–Trait1 (DB or IDP)–Trait2 (IDP or DB) chains. For example, one such chain is rs3130455–*CCHCR1* in excitatory neurons–SCZ–PTR (effect direction: +, +, +), where rs3130455 is marked by active promoter chromatin states (ChromHMM 1_TssA) across neural lineages and disrupts transcription factor motifs implicated in neurodevelopment such as *Klf4* and *Klf7*. It suggests that increased SNP dosage may elevate the expression level of *CCHCR1* in excitatory neurons, thereby elevating SCZ risk, and further enhancing white matter connectivity in PTR. Another example is rs55938136–*MAPT* in astrocytes–genu of corpus callosum (GCC)–SCZ (effect direction: +, –, +), where the intronic SNP rs55938136 showed perfect colocalization with *MAPT* expression in astrocytes and the FA value of GCC (PP.H4 = 1, PP.H4.snp = 1). This indicates that increased SNP dosage may raise *MAPT* expression in astrocytes, and reduce structural connectivity of GCC, and consequently increase SCZ susceptibility [113, 114]. The full list of chains is summarized in **S13 Table**. |

# *Reviewer #3:*

*The authors present a careful set of analyses and results aimed at delineating causal relationships between the eGene expression in specific cell types and (1) brain disorders and behaviors (mostly psychiatric disorders) and (2) image-derived phenotypes. To do so, they used cis-eQTLs derived in 8 brain cell types (previously published by Bryois et al) and published results from GWAS/TWAS studies. Causal inference between eGenes and disorders/phenotypes were performed using the machinery behind Mendelian randomization, with a key novel hallmark of this study being the fact that MR was performed at the level of distinct cell types (in contrast to previous studies of this general nature). The authors identified hundreds of candidate causal genes for dozens of different brain-related traits of biomedical interest. Further downstream analyses of the causal eGenes was performed to investigate pleiotropy, and they also evaluate the potential causal routes at play. Spatiotemporal expression data from the identified genes were used to examine links between the studied phenotypes and expression.*

**REF3 – Response to REF3:** We would like to thank you for the thoughtful review of our manuscript, and your suggestions are very precious to our work. Our responses are summarized point-by-point below. We believe that the additional changes we have made improved our manuscript.

**REF3 – Point to point response to REF3**

*Major comments:*

## REF 3.1 – Clarifying the principles of MR and causal inference

| *Referee Comment* | *The most important limitation in this study is not in the narrative, results, and analyses, but rather in the exposition and delineation of the underlying principles underlying MR and causal inference. To be more specific -- although the authors could refer to published reviews, textbooks, protocols etc to explain the mechanics of how Mendelian randomization works (for ex - see ref: Sanderson, Eleanor, M. Maria Glymour, Michael V. Holmes, Hyunseung Kang, Jean Morrison, Marcus R. Munafò, Tom Palmer et al. "Mendelian randomization." Nature reviews Methods primers 2, no. 1 (2022): 6.), I feel that the paper would be greatly improved if the authors provided a dedicated section to explain the principles behind MR more clearly (this section could just consist of a few paragraphs and a few schematics, and it can even be placed in in the Supplement if needed). Although a small schematic does appear within a sub-panel of Fig. 1a(ii), a fuller and clearer explanation would substantially boost the accessibility of this manuscript on the whole.*  *On that note, the authors can include 1 or 2 paragraphs (also in the supplement) to explain the underlying theory behind causal inference -- for example: the machinery governing how one can infer causality routes among cell type-specific the causal eGenes, DBs, and IDPs. These explanations would not need to be overly formal or even mathematically dense. It's the intuition that is needed (I’d also ask the authors to emphasize the importance of potential confounders in this type of analysis).* |
| --- | --- |
| Author Response | Thank you for your suggestion. We totally agree that a more explicit and intuitive explanation of MR would greatly enhance the accessibility of our study, particularly for readers unfamiliar with MR. In the revised manuscript, we have added a new section (**S3 Text**) that provides an intuitive introduction to MR and illustrates the causal inference framework in this study using plain language and simple formulas. |
| Excerpt 3.1 (Supplementary Text) | **Lines 813-814 (Methods):**  We also included a brief introduction to MR for non-expert readers in **S3 Text**.  **S3 Text:**  Overview of Mendelian Randomization  A key challenge in human genetics is determining whether a biological factor causes a disease or is simply correlated with it. In observational studies, exposures (i.e., risk factors) and outcomes are often influenced by confounding factors such as age, lifestyle, environment, or cellular composition, which obscure the true direction of causality.  Randomized controlled trials are considered as the gold standard for testing causality. However, RCTs are often impractical due to ethical constraints. As an alternative, Mendelian randomization (MR) introduces the concept of instrumental variables (IVs) from econometrics, and uses genetic variants as IVs to estimate the causal effect of an exposure (e.g., gene expression) on an outcome (e.g., disease or behavior). Because genetic variants are fixed at conception and largely unaffected by environmental influences, they offer a source of exogenous variation that is less prone to confounding factors^5^. Therefore, if the genetically predicted exposure variation is associated with an outcome, it supports a putative causal effect of the exposure on the outcome.  In general, effective MR methods rely on three core assumptions: (i) Relevance: each genetic variant is associated with the exposure; (ii) Independence: each genetic variant is independent of confounding factors; (iii) Exclusion restriction: each genetic variant influences the outcome only through the exposure. For MR methods such as MR-Egger that explicitly model horizontal pleiotropy (variants influence the outcome directly), they relax the third assumption and instead require the weaker InSIDE (Instrument Strength Independent of Direct Effect) condition^6^. Because violations of these assumptions can bias causal estimates, MR analyses typically include tests for horizontal pleiotropy, weak instrument bias, and heterogeneity to ensure robustness^6-9^. As long as the genetic instruments satisfy the three core IV assumptions and an additional point-estimate-identifying assumption (e.g., the genetic variant affects the exposure in the same direction across all individuals, typically assumed in standard MR models), the null hypothesis of no causal effect is sufficient to test validly and the resulting exposure-outcome relationship is biologically interpretable^5^.  The question of whether to select independent SNPs or correlated SNPs as IVs remains a pending issue in MR. On the one hand, exposure variance can be affected by multiple SNPs which are in potential linkage disequilibrium (LD) with each other. On the other hand, incorporating more SNPs can elevate the risk of bias in MR estimations caused by weak instrument bias or pleiotropy as irrelevant SNPs are also included in analyses. To balance this, our study applied multiple complementary MR methods with different IV selection strategies and pleiotropy assumptions.  MR methods can be categorized into one-sample MR and two-sample MR. In one-sample MR, individual-level data for the genotype, exposure, and outcome are obtained from the same cohort. The causal effect is typically estimated using two-stage least squares (2SLS) framework. In the first stage, the exposure is predicted from the IVs using the following linear model: $\boldsymbol{X}=\pi_{0}\boldsymbol{I}+\pi_{1}\boldsymbol{G}+\boldsymbol{\epsilon}_{1}$where $\boldsymbol{G}$ is the genotype matrix for instrumental SNPs, $\boldsymbol{X}$ is the observation vector for the exposure (assuming a single exposure for simplicity), $\pi_{1}$ is the regression coefficient, $\pi_{0}$ is the intercept, and $\boldsymbol{\epsilon}_{1}$ is the error term. In the second stage, the outcome is regressed on the predicted exposure: $\boldsymbol{Y}=\beta_{0}\boldsymbol{I}+\beta_{1}\hat{\boldsymbol{X}}+\boldsymbol{\epsilon}_{2}$where $\hat{\boldsymbol{X}}$ is the genetically predicted exposure from the first stage, $\boldsymbol{Y}$ denotes the observation vector for the outcome, $\beta_{1}$ is the causal effect, $\beta_{0}$ is the intercept, and $\boldsymbol{\epsilon}_{2}$ is the error term.    **Schematic of Mendelian Randomization.**  Two-sample MR uses GWAS summary statistics from two independent datasets: one for the exposure and one for the outcome. This enables causal inference in the absence of individual-level data. The most basic estimator in two-sample MR is the inverse-variance weighted (IVW) method^10-12^. Suppose $k$ is the number of independent SNPs selected as IVs, and $\hat{\beta}_{X_{i}}$ and $\hat{\beta}_{Y_{i}}$ denote the GWAS estimate of SNP $i$ on the exposure and the outcome, respectively. The causal effect estimate for SNP $i$ is then given by the Wald ratio: $\hat{\beta}_{{XY}_{i}}=\frac{\hat{\beta}_{Y_{i}}}{\hat{\beta}_{X_{i}}}$Its standard error can be approximated as^13^: $\mathrm{se}\left( \hat{\beta}_{{XY}_{i}} \right)\approx\frac{\mathrm{se}\left( \hat{\beta}_{Y_{i}} \right)}{\hat{\beta}_{X_{i}}}$The IVW estimator then combines the SNP-specific causal effects using inverse-variance weighting: $\hat{\beta}^{\mathrm{IVW}}=\frac{\sum_{i=1}^{k} \frac{\hat{\beta}_{{XY}_{i}}}{\mathrm{Var}\left( \hat{\beta}_{{XY}_{i}} \right)}}{\sum_{i=1}^{k} \frac{1}{\mathrm{Var}\left( \hat{\beta}_{{XY}_{i}} \right)}}=\frac{\sum_{i=1}^{k} \frac{{\hat{\beta}_{X_{i}}\hat{\beta}}_{Y_{i}}}{\mathrm{Var}\left( \hat{\beta}_{Y_{i}} \right)}}{\sum_{i=1}^{k} \frac{\hat{\beta}_{X_{i}}^{2}}{\mathrm{Var}\left( \hat{\beta}_{Y_{i}} \right)}}$It has been proven that 2SLS and IVW are asymptotically equivalent under ideal conditions (two samples are ancestry-matched, mutually independent, and of sufficiently large sample size)^14^. So two-sample MR can be viewed as an practical approximation to one-sample MR.  Causal inference framework in this study  In this study, the aim is to identify causal relationships between cell type-specific gene expression and a broad range of brain-associated complex traits. Due to the lack of individual-level data, we adopted a two-sample MR framework based on publicly available GWAS and eQTL summary statistics. In this context, *cis*-eQTLs measured in eight major brain cell types serve as IVs, gene expression serves as the exposure, and brain imaging-derived phenotypes (IDPs) and brain disorders or behaviors (DBs) serve as the outcomes.  The intuition is straightforward: *cis*-eQTLs provide exogenous variation in gene expression that is unlikely to be influenced by environmental or behavioral confounders. Then if a *cis*-eQTL-driven change in expression consistently predicts variation in a trait (IDP or DB) across multiple MR methods, it supports a putative causal effect of gene expression on the trait. Building on this idea, we further characterized putative causal routes among eGenes, IDPs, and DBs by integrating significant associations across eGene-IDP, eGene-DB and IDP-DB pairs. These routes provide hypotheses on how gene regulation in specific brain cell types may influence brain structure and thereby affect disease risk, or how gene regulation may influence disease-related processes and in turn alter brain structure. We caution that these routes should not be interpreted as definitive causal pathways. They highlight possible regulatory mechanisms underlying brain-associated complex traits, and require further experimental validation. |

## REF 3.2 – Explain on sample exclusion due to UKB overlap

| *Referee Comment* | *It is not clear why the inclusion of data from UK Biobank would entail removing samples (eg, see lines 568-569).* |
| --- | --- |
| Author Response | Thanks for the comment. In two-sample MR analyses, it is critical to make sure the GWAS datasets used for the exposure and outcome are mutually independent, as sample overlap can bias the causal effect estimates and inflate false positives(35, 36). Since the GWAS summary statistics for IDPs in our study were from UK Biobank, we carefully screened all DB GWAS datasets to exclude those also included UK Biobank samples. Given UK Biobank is widely used in large-scale GWAS studies, this filtering step was necessary to avoid sample overlap and ensure the validity of our causal inferences between IDPs and DBs. We acknowledge that this step reduced the completeness of results, and have noted this limitation in the manuscript (**Lines 646-652**). |
| Excerpt 3.2 (Main Text) | **Lines 646-652 (Discussion):**  Second, when performing MR between IDPs and DBs, half of the GWASs of DBs were removed due to the inclusion of samples from the popular dataset UK Biobank (UKB) [134], thereby limiting the completeness of inferred putative causality routes among eGenes, IDPs, and DBs (**Fig. 4**). At the same time, shared causal eGenes identified across IDPs and/or DBs might be influenced by unavoidable sample overlap between some GWAS datasets. Future work could aim to collect more fully independent GWAS resources for brain-related traits to improve the robustness and coverage of these causal inferences. |

## REF 3.3 – Choice of single-cell eQTL dataset and use of brainSCOPE

| *Referee Comment* | *The Bryois et al dataset contains scQTLs in 8 cell types from 192 individuals. But the Emani et al data contains scQTLs in >15 cell types from a starting dataset size 388 individuals, which would presumably boost both statistical power and biological resolution (with more annotated cell types). If the authors decide to keep the Bryois set of QTLs (which admittedly has the advantage of a more interpretable story with more broadly-defined cell types), then they can perhaps point to the Emani data as an avenue for future/extended studies of this nature. -- ref: Emani, Prashant S., et al. "Single-cell genomics and regulatory networks for 388 human brains." Science 384.6698 (2024): eadi5199.* |
| --- | --- |
| Author Response | Thank you for this valuable suggestion. We fully agree that larger single-cell QTL resources with more individuals and finer cell-type annotation can improve both statistical power and biological resolution. We didn’t use dataset from *Emani et al. 2024* (the *brainSCOPE* resource) as discovery dataset because the 388 samples include a small proportion of non-European individuals(25), whereas two-sample MR analyses require ancestry-matched exposure and outcome datasets (described in **REF 3.1**). In the revised manuscript, rather than leaving *Emani et al. 2024* as a future direction, we have incorporated this dataset as an independent replication resource to validate the predicted cell type-specific causal eGenes identified using *Bryois et al*. *2022*.  After harmonizing the cell type definitions between *brainSCOPE* and the *Bryois et al* dataset used for our main analysis, we evaluated the six major cell types: astrocytes, excitatory neurons, inhibitory neurons, oligodendrocytes, OPCs, and microglia. The same MR analysis pipeline was applied to *brainSCOPE* eQTL summary statistics to estimate causal effects of cell type-specific gene expression on 149 brain-associated complex traits. An eGene-cell type-trait triple was considered replicated if it reached nominal significance in at least one MR method, and showed consistent effect directions across all MR methods and with the discovery analysis. The results are as follows:   - 41.9% (315/751; *P* < 10^-16^, hypergeometric test) of eGene-cell type-IDP triples were replicated at *P* < 0.05, and 33.7% (253/751; *P* < 10^-16^, hypergeometric test) were replicated at FDR < 0.05 (**S3 Fig**). - 28.7% (84/293; *P* < 10^-16^, hypergeometric test) of eGene-cell type-DB triples were replicated at *P* < 0.05, and 23.5% (69/293; *P* < 10^-16^, hypergeometric test) were replicated at FDR < 0.05 (**S3 Fig**).   These results support that a substantial proportion of the cell-type specificity of our predicted causal eGenes can be independently validated using a larger single-cell eQTL resource. Relevant changes have been made in **Results** (**Lines 164-170, Lines 188-199**), **Methods** (**Lines 848-860**), **Discussion (Lines 639-646), S3 Fig, S5 Table**, and **S7 Table**.    **S3 Fig. Replication results for the predicted gene-cell type-IDP and gene-cell type-DB associations.** Reproducibility of eGene-cell type-IDP triples was assessed using cell type-specific eQTL summary statistics from brainSCOPE(25). Reproducibility of eGene-cell type-DB triples was assessed using cell type-specific eQTLs from brainSCOPE(25) and differentially expressed genes from Mathys et al (only AD-associated causal eGenes were tested)(24). Significance of overlap was tested by hypergeometric test. |
| Excerpt 3.3 (Main Text) | **Lines 164-170 (Results):**  To confirm the reproducibility of the predicted eGene-IDP pairs and eGene-cell type-IDP triples, we repeated MR analyses using cortical eQTL summary statistics from BrainMeta [36] and cell type-specific eQTL summary statistics from brainSCOPE [37], respectively. Among the eGene-IDP pairs, 64.9% (430/663) and 49.2% (326/663) were replicated in BrainMeta at nominal and FDR < 0.05 levels, respectively (both *P* < 10^-16^, hypergeometric test; **S5 Table** and **S2 Fig**). Among the eGene-cell type-IDP triples, 41.9% (315/751) and 33.7% (253/751) were replicated in brainSCOPE at nominal and FDR < 0.05 levels, respectively (both *P* < 10^-16^, hypergeometric test; **S5 Table** and **S3 Fig**).  **Lines 188-199 (Results):**  As we did for the IDPs, we confirmed the reproducibility of eGene-DB pairs using three datasets: the cortical eQTL summary statistics from BrainMeta [36], differentially expressed genes and transcripts from PsychENCODE [29], and documented gene-trait associations from GWAS Catalog [1]. Replication rates were 35.7% (87/244; at nominal significance level) and 23.0% (56/244; at FDR < 0.05 level) in BrainMeta, 29.5% (23/78) in PsychENCODE, and 23.8% (57/240) in the GWAS Catalog (all *P* < 10^-4^, hypergeometric test; **S7 Table** and **S2 Fig**). We further confirmed the reproducibility of eGene-cell type-DB triples using two datasets: the cell type-specific eQTL summary statistics from brainSCOPE [37], and AD-related differentially expressed genes from Mathys et al [42]. We found 41.9% (315/751) and 33.7% (253/751) triples were replicated in brainSCOPE at nominal and FDR < 0.05 levels, respectively (both with *P* < 10^-16^, hypergeometric test; **S7 Table** and **S3 Fig**). Among the 14 AD-associated cell type-specific causal eGenes identified in our analysis, 12 were supported by Mathys et al.  **Lines 848-860 (Methods):**  To validate the predicted eGene–cell type–trait triples, two external datasets were used. (1) We obtained cell type-specific eQTL summary statistics from the brainSCOPE resource (https://brainscope.gersteinlab.org/integrative_files.html), which was generated using single cell-resolution data from 388 adult DLPFC samples [37]. Six major cell types were considered: astrocytes, excitatory neurons, inhibitory neurons, oligodendrocytes, OPCs, and microglia. The same MR analysis procedure was applied to this data to infer the causal relationships between cell type-specific gene expression and 149 brain-associated complex traits. An eGene–cell type–trait triple was considered replicated if it achieved nominal significance in at least one MR method, and showed consistent effect directions across all MR methods and with the discovery analysis. (2) We extracted differentially expressed genes associated with AD pathology in each cell type from Mathys et al. [42]. For each external dataset, we calculated the replication rate as the number of replicated associations divided by the total number of associations tested, and assessed the significance by hypergeometric test. Replication was assessed only for traits that appeared in both the discovery and replication studies.  **Lines 639-646 (Discussion):**  First, the lower number of *cis*-eQTLs in endothelial cells and pericytes, compared to other cell types (**S3 Table**) [12], likely limited the number of causal genes identified in these cell types (**Fig. 1b**). This imbalance in eQTL availability may partially affect the observed cell-type specificity patterns of eGene-IDP and eGene-DB associations, particularly for cell types with limited instrument availability. With larger brain single-cell-derived gene expression datasets that have increased sample sizes and finer cell type annotation, we are expected to improve the identification of biologically meaningful genes involved in brain illnesses within these cell types. |

## REF 3.4 – Ambiguous usage of phrase

| *Referee Comment* | *Throughout -- the language "cell type-specific eGenes" and "cell type-specific causal eGenes" is ambiguous. In some of the literature, the term "cell type-specific eGenes" denotes eGenes that show up exclusively in one cell type only (and not any others) -- whereas elsewhere, the term may be more loosely defined as showing up in a small number of cell types (but more than one). In yet other published work, the term is very loosely defined to simply mean eGenes that are identified using snRNAseq data -- even if the eGene shows up as significant in a large number of cell types.* |
| --- | --- |
| Author Response | Sorry for the ambiguity. In the revised manuscript, we added this definition of the term “cell type-specific causal eGenes” as genes whose expression exhibit statistically significant causal associations with a trait in a specific cell type in MR analysis, **regardless of whether the same gene may also be causal in other cell types**. To avoid confusion, we have replaced all the phrase “cell type-specific eGenes” with “cell type-specific causal eGenes” throughout the manuscript. |
| Excerpt 3.4 (Main Text) | **Lines 138-141 (Results):**  We analyzed the trait pleiotropy of these cell type-specific causal eGenes (defined as genes that show statistically significant causal associations with a trait in a specific cell type in MR analysis) across different classes and groups of these brain-associated complex traits. |

## REF 3.5 – Comorbidity between disorders

| *Referee Comment* | *Regarding the results on cell type-specific causal eGenes that are common between psychiatric disorders (for example, the results showcased in Fig 3b) -- an analysis (or at least a discussion) regarding comorbidity between the different psychiatric disorders may really add value. The logic here is that if two disorders share many causal eGenes, one may expect that this pair of disorders exhibits high comorbidity. Do the authors observe this to be the case (of course, this is not guaranteed to be the case -- shared causal eGenes would imply comorbidity of the eSNPs are also shared). But the authors may be able to check for this without having to carry out the comorbidity analysis themselves -- instead, catalogs of pairwise comorbidity can probably just be checked in recent studies. Many of those results should be available in the literature -- for just handful of examples, maybe see:*  *- McGrath et al., 2020 — “Comorbidity within mental disorders.” World Psychiatry. : https://pmc.ncbi.nlm.nih.gov/articles/**PMC7443806/?utm_source=chatgpt.com*  *- Bourque et al., 2024 — “Genetic and phenotypic similarity across major psychiatric disorders.” Molecular Psychiatry (systematic review). : https://www.nature.com/articles/s41398-024-02866-3?utm_source=chatgpt.com*  *- Solmi et al., 2023 — “Meta-analytic prevalence of comorbid mental disorders in clinical high-risk for psychosis (CHR-P).” Molecular Psychiatry (meta-analysis). : https://www.nature.com/articles/s41380-023-02029-8?utm_source=chatgpt.com*  *- Kessler et al., 2005 — NCS-R 12-month prevalence & comorbidity (JAMA Psychiatry). -- https://jamanetwork.com/journals/jamapsychiatry/fullarticle/208671?utm_source=chatgpt.com*  *Maybe see also:*  *- Platona, Rita Ioana, et al. "The impact of psychiatric comorbidities associated with depression: a literature review." Medicine and pharmacy reports 97.2 (2024): 143.*  *- Lee, Sool, et al. "Massively parallel reporter assay investigates shared genetic variants of eight psychiatric disorders." Cell 188.5 (2025): 1409-1424.*  *- Raznahan, Armin, et al. "Convergence and divergence of rare genetic disorders on brain phenotypes: a review." JAMA psychiatry 79.8 (2022): 818-828.* |
| --- | --- |
| Author Response | We thank the reviewer for this valuable suggestion. To explore whether disorders that share cell type-specific causal eGenes also exhibit comorbidity in previous studies, we first constructed a trait × trait Jaccard similarity matrix based on shared cell type-specific causal eGenes between each pair of traits (see **S1 Text**). Then we applied hierarchical clustering (R package *pvclust*, 10,000 bootstraps) for this similarity matrix(27), and identified 12 robust trait clusters (**S11 Fig** and **S10 Table**).  **S11 Fig. Hierarchical clustering of the aggregate trait similarity matrix derived from shared cell type-specific causal eGenes.** For each of the cell types, we constructed a trait × trait Jaccard similarity matrix based on shared causal eGenes. These were averaged to generate an aggregate similarity matrix, followed by hierarchical clustering. The resulting 12 trait clusters (C1 ~ C12) are annotated on the heatmap. To evaluate possible comorbidity, we focused on all disorder-disorder pairs (psychiatric-psychiatric, psychiatric-neurological or neurological- neurological pairs) that co-occurred within the same cluster. The assumption is straightforward: disorders that are grouped together based on shared causal eGenes are more likely to share upstream biological mechanisms, and this may reflect clinical comorbidity. For each of these disorder pairs, we manually searched the literature in PubMed to identify support for (i) explicit comorbidity and (ii) suggestive association, including longitudinal risk studies, Mendelian randomization, and genetic correlation evidence. A total of 22 disorder-disorder pairs were identified across clusters (**S11 Table**). Among them, all 9 psychiatric disorder pairs had strong comorbidity support in previous studies, such as the pair of bipolar disorder (BIP) and schizophrenia (SCZ), and BIP and anxiety disorder (ANX)(37, 38). For neurological-psychiatric and neurological-neurological disorder pairs, although less frequently examined together, several pairs still showed strong comorbidity support or suggestive association. For example, the pair of epilepsy and PTSD in cluster 6 is supported by clinical evidence of comorbidity(39). The pair of ischemic stroke (IS) and obsessive-compulsive disorder (OCD) in cluster 7 is supported by a longitudinal study reporting elevated stroke risk among OCD patients(40). The pair of Parkinson’s disease (PD) and alcohol use disorder (AUD) in cluster 12 is supported by epidemiological evidence reporting increased AUD prevalence among PD patients(41). These findings suggest that shared cell type-specific causal eGenes not only highlight known comorbidity relationships but may also reveal underexplored links worthy of further investigation. **S11 Table.** Literature support for disorder-disorder pairs co-clustered based on shared cell type-specific causal eGenes. This table summarizes disorder pairs that appear in the same cluster derived from the aggregate trait × trait Jaccard similarity matrix (**S11 Fig**), along with evidence from previous literature. NEUD: Neurological disorder; PSYD: Psychiatric disorder. **Cluster** \| **Trait1** \| **Group1** \| **Trait2** \| **Group2** \| **Evidence Type** \| **PMID**; C4 \| BIP \| PSYD \| SCZ \| PSYD \| explicit comorbidity \| 34225788; C4 \| BIP \| PSYD \| ANX \| PSYD \| explicit comorbidity \| 34225788; C4 \| BIP \| PSYD \| MDD \| PSYD \| explicit comorbidity \| 34225788; C4 \| SCZ \| PSYD \| ANX \| PSYD \| explicit comorbidity \| 34225788; C4 \| SCZ \| PSYD \| MDD \| PSYD \| explicit comorbidity \| 34225788; C4 \| ANX \| PSYD \| MDD \| PSYD \| explicit comorbidity \| 32203749; C6 \| EPI \| NEUD \| PTSD \| PSYD \| explicit comorbidity \| 39919539; C6 \| EPI \| NEUD \| ADHD \| PSYD \| explicit comorbidity \| 37326829; C6 \| PTSD \| PSYD \| ADHD \| PSYD \| explicit comorbidity \| 32782057; C7 \| IS \| NEUD \| ASD \| PSYD \| suggestive association \| 38640796; C7 \| IS \| NEUD \| OCD \| PSYD \| suggestive association \| 34039028; C7 \| IS \| NEUD \| TS \| PSYD \| \|; C7 \| ICH \| NEUD \| IS \| NEUD \| suggestive association \| 40267100; C7 \| ICH \| NEUD \| ASD \| PSYD \| \|; C7 \| ICH \| NEUD \| OCD \| PSYD \| \|; C7 \| ICH \| NEUD \| TS \| PSYD \| \|; C7 \| ASD \| PSYD \| OCD \| PSYD \| \|; C7 \| ASD \| PSYD \| TS \| PSYD \| explicit comorbidity \| 27375503; C7 \| OCD \| PSYD \| TS \| PSYD \| explicit comorbidity \| 27375503; C12 \| ALS \| NEUD \| PD \| NEUD \| suggestive association \| 37534731; C12 \| ALS \| NEUD \| AUD \| PSYD \| suggestive association \| 25641323; C12 \| PD \| NEUD \| AUD \| PSYD \| suggestive association \| 34336520 We have added a new section in Results (**Lines 387-414**) and Supplementary Text (**S1 Text**), and included **S11 Table** summarizing all disorder pairs and literature support. |
| Excerpt 3.5 (Main Text and Supplementary Text) | **Lines 387-414 (Results):**  **Cell type-informed clustering of traits reveals biologically meaningful patterns**  To explore the common regulatory basis across traits, we computed a trait × trait Jaccard similarity matrix based on shared cell type-specific causal eGenes (**S11 Fig;** also see **S1 Text**). Oligodendrocytes, excitatory neurons, and astrocytes emerged as the top contributors to the global trait similarity pattern (**S12 Fig**), in line with their higher numbers of trait-associated causal eGenes (**Fig. 1b-c**). Hierarchical clustering of the similarity matrix revealed 12 robust trait clusters (**S11 Fig** and **S10 Table**). Notably, eight clusters (C2 ~ C9 and C12) contained traits from both IDP and DB classes (**S13 Fig**). Clusters C6 and C7 were the most heterogeneous, each spanning all five trait groups. In both C6 and C7, inhibitory neurons emerged as the top contributors, highlighting a key role in mediating shared molecular regulation across diverse phenotypic categories. Cluster C4 exhibited significant enrichment for white matter microstructure phenotypes (FDR = 0.0014, hypergeometric tests) and a nominal enrichment for psychiatric disorders (*P* = 0.03, hypergeometric tests), with microglia emerging as the dominant cell type, pointing to cell type-specific regulatory mechanisms underlying this cluster.  Given that comorbidity is common and clinically important among mental disorders [95-97], we further asked whether these trait clusters also reflect known comorbidity patterns among disorders. We focused on all disorder-disorder pairs grouped into the same cluster, and manually searched the literature to identify support for explicit comorbidity and suggestive association. Among the 22 disorder pairs identified (**S11 Table**), all 9 psychiatric disorder pairs had strong comorbidity support in previous studies, such as the pair of BIP and SCZ, and BIP and anxiety disorder (ANX) [98, 99]. For neurological-psychiatric and neurological-neurological disorder pairs, although less studied together, several pairs still showed strong comorbidity support or suggestive association in previous longitudinal or genetic studies. For instance, the pair of epilepsy and PTSD in cluster 6 is supported by clinical evidence of comorbidity [100]. The pair of ischemic stroke (IS) and obsessive-compulsive disorder (OCD) in cluster 7 is supported by a longitudinal study reporting elevated stroke risk among OCD patients [101]. The pair of PD and alcohol use disorder (AUD) in cluster 12 is supported by epidemiological evidence reporting increased AUD prevalence among PD patients [102]. These findings indicate that our trait clusters defined by shared cell type-specific causal eGenes not only capture molecular similarity but may also reflect clinically meaningful comorbidity patterns.  S1 Text:  Trait similarity matrix construction and clustering analysis  For each brain cell type, we constructed a trait × trait Jaccard similarity matrix based on the predicted causal eGenes. The Jaccard index for each trait pair was calculated as the number of their shared causal eGenes divided by the number of their causal eGenes union. These cell type-specific matrices were then averaged to produce an aggregate similarity matrix representing global trait similarity across cell types. To evaluate which cell types mostly drive this global similarity pattern, we computed the Pearson correlation between each cell type-specific matrix and the aggregate matrix.  Hierarchical clustering was next performed on the aggregate similarity matrix using R package *pvclust* (version 2.2) with average linkage and correlation distance^1^. Cluster stability was assessed through 10,000 bootstrap replicates, and clusters with approximately unbiased *P*-values ≥ 0.95 were considered significant. To evaluate which cell types mostly drive within-cluster similarity pattern, we calculated the Pearson correlation between each cell type-specific matrix and the aggregate matrix and restricted to the traits within a given cluster. We further performed trait group enrichment within clusters using hypergeometric tests and corrected for multiple testing using the false discovery rate (FDR). |

*Minor comments:*

## REF 3.6 – Misuse of author's first name

| *Referee Comment* | *"Julien et al[12]" --> "Bryois et al[12]" (Julien is this author's first name; Bryois is the last name)* |
| --- | --- |
| Author Response | Thanks for pointing this out. We have corrected “Julien et al” to “Bryois et al” in our revised manuscript. |

## REF 3.7 – Cite the proper reference

| *Referee Comment* | *For GWAS -- maybe just cite: Uffelmann, Emil, et al. "Genome-wide association studies."* *Nature Reviews Methods Primers 1.1 (2021): 59.* |
| --- | --- |
| Author Response | Thanks for this comment. In the revised manuscript, we have added a citation to this literature in the first sentence of Introduction. |
| Excerpt 3.7 (Main Text) | **Lines 47-48 (Introduction):**  Recent large-scale genome-wide association studies (GWASs) have identified thousands of genetic variants associated with complex traits of human brain [1, 2]. |

## REF 3.8 – Provide clear figures

| *Referee Comment* | *The figure resolutions are not ideal (but this could potentially be because of the review-stage PDF)* |
| --- | --- |
| Author Response | In the revision stage, we have uploaded all figures as high-resolution TIFF files to ensure image clarity. |

## REF 3.9 – Modification of phrase

| *Referee Comment* | *"**an analytic framework for causality identification" --> "an analytic framework for causal inference"* |
| --- | --- |
| Author Response | In light of the comment, we have modified the phrase “an analytic framework for causality identification” as “an analytic framework for genetic causal inference” in our revised manuscript (**Lines 54-57**). |
| Excerpt 3.9 (Main Text) | **Lines 54-57 (Introduction):**  Therefore, the integration of *cis*-eQTLs and GWAS findings for identifying the causal genes could be promising for understanding the biological mechanisms underlying these complex traits, which can be achieved by Mendelian randomization (MR), an analytic framework for genetic causal inference. |

## REF 3.10 – Constraint genes

| *Referee Comment* | *line 50-52: w.r.t. "It has been revealed that cell type-level cis-eQTLs could have larger effect sizes and target more constrained genes than tissue-level cis-eQTLs[12]." --> By "constrained", are the authors referring to evolutionary constraint?* |
| --- | --- |
| Author Response | Yes, by “constrained genes,” we refer to genes under strong evolutionary constraint, typically quantified by metrics such as pLI or LOEUF, which indicate intolerance to loss-of-function mutations. |
| Excerpt 3.10 (Main Text) | **Lines 62-64 (Introduction):**  It has been revealed that cell type-level *cis*-eQTLs could have larger effect sizes and target more evolutionarily constrained genes than tissue-level *cis*-eQTLs [12]. |

## REF 3.11 – Modification of sentence

| *Referee Comment* | *"There is still a long road lays ahead in early treatment" --> "There is still a long road that lays ahead in early treatment"* |
| --- | --- |
| Author Response | Thank you for the comment. We have revised as “There is still a long road that lays ahead in early treatment” in the revised manuscript (**Lines 654-655**). |

# References

1.	Sanderson E, Glymour MM, Holmes MV, Kang H, Morrison J, Munafò MR, et al. Mendelian randomization. Nat Rev Methods Primers. 2022;2.

2.	Han Z, Zhu Y, Xia Z, Deng Q, He H, Yin Q, et al. Genetic analyses identify circulating genes related to brain structures associated with Parkinson's disease. NPJ Parkinsons Dis. 2025;11(1):17.

3.	Zhou D, Jiang Y, Zhong X, Cox NJ, Liu C, Gamazon ER. A unified framework for joint-tissue transcriptome-wide association and Mendelian randomization analysis. Nat Genet. 2020;52(11):1239-46.

4.	Zhao B, Shan Y, Yang Y, Yu Z, Li T, Wang X, et al. Transcriptome-wide association analysis of brain structures yields insights into pleiotropy with complex neuropsychiatric traits. Nature communications. 2021;12(1):2878.

5.	Yang A, Rolls ET, Dong G, Du J, Li Y, Feng J, et al. Longer screen time utilization is associated with the polygenic risk for Attention-deficit/hyperactivity disorder with mediation by brain white matter microstructure. EBioMedicine. 2022;80:104039.

6.	Geng X, Li G, Lu Z, Gao W, Wang L, Shen D, et al. Structural and Maturational Covariance in Early Childhood Brain Development. Cereb Cortex. 2017;27(3):1795-807.

7.	Hofer E, Roshchupkin GV, Adams HHH, Knol MJ, Lin H, Li S, et al. Genetic correlations and genome-wide associations of cortical structure in general population samples of 22,824 adults. Nature communications. 2020;11(1):4796.

8.	Zhao B, Li T, Smith SM, Xiong D, Wang X, Yang Y, et al. Common variants contribute to intrinsic human brain functional networks. Nat Genet. 2022;54(4):508-17.

9.	Zhao X, Yang A, Zhang Z-C, Yang YT, Zhao X-M. Deciphering the genetic architecture of human brain structure and function: a brief survey on recent advances of neuroimaging genomics. Briefings in bioinformatics. 2023;24(2):bbad060.

10.	Zhao B, Li T, Yang Y, Wang X, Luo T, Shan Y, et al. Common genetic variation influencing human white matter microstructure. Science (New York, NY). 2021;372(6548).

11.	Bulik-Sullivan B, Finucane HK, Anttila V, Gusev A, Day FR, Loh PR, et al. An atlas of genetic correlations across human diseases and traits. Nat Genet. 2015;47(11):1236-41.

12.	Qi T, Wu Y, Fang H, Zhang F, Liu S, Zeng J, et al. Genetic control of RNA splicing and its distinct role in complex trait variation. Nat Genet. 2022;54(9):1355-63.

13.	Gandal MJ, Zhang P, Hadjimichael E, Walker RL, Chen C, Liu S, et al. Transcriptome-wide isoform-level dysregulation in ASD, schizophrenia, and bipolar disorder. Science. 2018;362(6420).

14.	Sollis E, Mosaku A, Abid A, Buniello A, Cerezo M, Gil L, et al. The NHGRI-EBI GWAS Catalog: knowledgebase and deposition resource. Nucleic Acids Res. 2023;51(D1):D977-D85.

15.	Haglund A, Zuber V, Abouzeid M, Yang Y, Ko JH, Wiemann L, et al. Cell state-dependent allelic effects and contextual Mendelian randomization analysis for human brain phenotypes. Nat Genet. 2025;57(2):358-68.

16.	Yuan Z, Zhu H, Zeng P, Yang S, Sun S, Yang C, et al. Testing and controlling for horizontal pleiotropy with probabilistic Mendelian randomization in transcriptome-wide association studies. Nature communications. 2020;11(1):3861.

17.	Yuan Z, Liu L, Guo P, Yan R, Xue F, Zhou X. Likelihood-based Mendelian randomization analysis with automated instrument selection and horizontal pleiotropic modeling. Sci Adv. 2022;8(9):eabl5744.

18.	Guo J, Yu K, Dong SS, Yao S, Rong Y, Wu H, et al. Mendelian randomization analyses support causal relationships between brain imaging-derived phenotypes and risk of psychiatric disorders. Nat Neurosci. 2022;25(11):1519-27.

19.	Song W, Qian W, Wang W, Yu S, Lin GN. Mendelian randomization studies of brain MRI yield insights into the pathogenesis of neuropsychiatric disorders. BMC genomics. 2021;22(Suppl 3):342.

20.	Yang A, Yang YT, Zhao XM. An augmented Mendelian randomization approach provides causality of brain imaging features on complex traits in a single biobank-scale dataset. PLoS Genet. 2023;19(12):e1011112.

21.	Barbeira AN, Dickinson SP, Bonazzola R, Zheng J, Wheeler HE, Torres JM, et al. Exploring the phenotypic consequences of tissue specific gene expression variation inferred from GWAS summary statistics. Nature communications. 2018;9(1):1825.

22.	Haglund A, Zuber V, Yang Y, Abouzeid M, Feleke R, Ko JH, et al. Single-cell Mendelian randomisation identifies cell-type specific genetic effects on human brain disease and behaviour. bioRxiv. 2022:2022.11. 28.517913.

23.	Fujita M, Gao Z, Zeng L, McCabe C, White CC, Ng B, et al. Cell subtype-specific effects of genetic variation in the Alzheimer's disease brain. Nat Genet. 2024;56(4):605-14.

24.	Mathys H, Peng Z, Boix CA, Victor MB, Leary N, Babu S, et al. Single-cell atlas reveals correlates of high cognitive function, dementia, and resilience to Alzheimer's disease pathology. Cell. 2023;186(20):4365-85 e27.

25.	Emani PS, Liu JJ, Clarke D, Jensen M, Warrell J, Gupta C, et al. Single-cell genomics and regulatory networks for 388 human brains. Science (New York, NY). 2024;384(6698):eadi5199.

26.	Bryois J, Calini D, Macnair W, Foo L, Urich E, Ortmann W, et al. Cell-type-specific cis-eQTLs in eight human brain cell types identify novel risk genes for psychiatric and neurological disorders. Nature neuroscience. 2022;25(8):1104-12.

27.	Suzuki R, Shimodaira H. Hierarchical clustering with P-values via multiscale bootstrap resampling. R package. 2013.

28.	Davey Smith G, Hemani G. Mendelian randomization: genetic anchors for causal inference in epidemiological studies. Hum Mol Genet. 2014;23(R1):R89-98.

29.	Wang G, Sarkar A, Carbonetto P, Stephens M. A simple new approach to variable selection in regression, with application to genetic fine mapping. J R Stat Soc Series B Stat Methodol. 2020;82(5):1273-300.

30.	Wallace C. A more accurate method for colocalisation analysis allowing for multiple causal variants. PLoS Genet. 2021;17(9):e1009440.

31.	Ward LD, Kellis M. HaploReg: a resource for exploring chromatin states, conservation, and regulatory motif alterations within sets of genetically linked variants. Nucleic Acids Res. 2012;40(Database issue):D930-4.

32.	Xu Q, Ni JJ, Han BX, Yan SS, Wei XT, Feng GJ, et al. Causal Relationship Between Gut Microbiota and Autoimmune Diseases: A Two-Sample Mendelian Randomization Study. Front Immunol. 2021;12:746998.

33.	Patel S, Mahon K, Wellington R, Zhang J, Chaplin W, Szeszko PR. A meta-analysis of diffusion tensor imaging studies of the corpus callosum in schizophrenia. Schizophr Res. 2011;129(2-3):149-55.

34.	Sivagnanasundaram S, Crossett B, Dedova I, Cordwell S, Matsumoto I. Abnormal pathways in the genu of the corpus callosum in schizophrenia pathogenesis: a proteome study. Proteomics Clin Appl. 2007;1(10):1291-305.

35.	Burgess S, Davies NM, Thompson SG. Bias due to participant overlap in two-sample Mendelian randomization. Genet Epidemiol. 2016;40(7):597-608.

36.	Inoue A, Solon G. Two-sample instrumental variables estimators. The Review of Economics and Statistics. 2010;92(3):557-61.

37.	Dong G, Feng J, Sun F, Chen J, Zhao XM. A global overview of genetically interpretable multimorbidities among common diseases in the UK Biobank. Genome Med. 2021;13(1):110.

38.	Hossain MM, Khan N, Sultana A, Ma P, McKyer ELJ, Ahmed HU, et al. Prevalence of comorbid psychiatric disorders among people with autism spectrum disorder: An umbrella review of systematic reviews and meta-analyses. Psychiatry Res. 2020;287:112922.

39.	Ertan D, Tarrada A, El-Hage W, Sanchez S, Four E, Mezouar N, et al. Prevalence of posttraumatic stress disorder in adults with epilepsy: A meta-analysis. Seizure. 2025;126:32-42.

40.	Chen MH, Tsai SJ, Su TP, Li CT, Lin WC, Chen TJ, et al. Increased Risk of Stroke in Patients With Obsessive-Compulsive Disorder: A Nationwide Longitudinal Study. Stroke. 2021;52(8):2601-8.

41.	Kaur J, Sandhu RK, Kubra KT, Canenguez Benitez JS, Onyeaka HK, Akter S, et al. Substance Use Disorders in Patients With Parkinson's Disease and Adverse Hospitalization Outcomes: A National Inpatient Study. Cureus. 2021;13(6):e16033.
