# Supplementary material for: Deciphering cell type-specific causal genetic effects on brain imaging-derived phenotypes and disorders with single-cell Mendelian randomization
Source: PLoS Comput Biol. 2026 Jun 17;22(6):e1014422. doi: 10.1371/journal.pcbi.1014422 (PMC13289931; doi:10.1371/journal.pcbi.1014422)
Supplement: Attachment — Submitted filename: Response_to_reviewers.docx [file pcbi.1014422.s039.docx]

College of Biomedical Engineering

Fudan University

Shanghai 200438, China

May 12, 2026

Dear Editor,

Thank you for your feedback on our manuscript entitled “**Deciphering cell type-specific causal genetic effects on brain imaging-derived phenotypes and disorders with single-cell Mendelian randomization**” (PCOMPBIOL-D-25-00949). We sincerely thank all reviewers for their second round of constructive and valuable feedback that we have used to improve the quality of our manuscript.

The reviewers’ comments have been numbered and laid out in italicized font, while our point-to-point response in normal font. Notably, any changes or modifications in the main text or supplementary text have been given in red. Reference numbers appearing within quoted manuscript excerpts follow the numbering in the Revised Manuscript, whereas references cited in our response text use a separate numbering system.

We are hopeful that the revised manuscript would be regarded as appropriate for **PLOS Computational Biology** publication. Thank you again for your consideration.

Best regards,

**Dr. Xing-Ming Zhao**

Professor

College of Biomedical Engineering

Fudan University

Shanghai, P.R. China

Tel. 86-21-55665546

Email: xmzhao@fudan.edu.cn

*Response Letter to*

**Deciphering cell type-specific causal genetic effects on brain imaging-derived phenotypes and disorders with single-cell Mendelian randomization**

**Reviewers' comments:**

# *Reviewer #1:*

*Summary: The authors address many of the concerns of the paper, though statistical issues in their approach persist. I do believe that these issues can be addressed largely through textual revisions and careful statement of these concerns throughout the text.*

**REF1 – Response to REF1:** We sincerely thank you for taking the time to review our work. Your comments are valuable to us, and we have taken them into second consideration for any further improvements. The detailed point-by-point responses are listed below.

**REF1 – Point to point response to REF1**

## REF 1.1 – Round2 - Issue of tissue matching in MR

| *Referee Comment* | *Round 2:* *The authors defend their use of eQTLs from one brain region by stating that this approach is (1) defensible via the assumptions of mendelian randomization, and (2) is common in MR, TWAS, and colocalization literature. (1) Examination of their mendelian randomization framework reveals potential issues in the independence assumption via this approach, as the association between eQTLs in tissue 1 and phenotype in tissue 2 will be confounded by the potential eQTL in tissue 2. (2) Moreover, while this approach has been somewhat common in the past, time has revealed multiple issues in this paradigm (PMID:* *39407214,* *35113864). As a result, I recommend that the authors either remove these off-target associations from their study, or describe the above issues in the results and discussion.*  *Round 1 recap：*  *One potential flaw in the analytical workflow is that the authors use cell type-specific eQTLs from prefrontal cortex, temporal cortex, and deep white matter to infer associations about other brain regions. The literature on whether this is appropriate is currently in flux. Currently the data suggests that the same cell type in different cortical regions in generally consistent (PMID: 39578476), but this is not necessarily the case subcortically (PMID: 37824663). Since there are few resources for single-cell data for most brain regions (excluding the dorsolateral prefrontal cortex), I recommend the authors limit their broader analyses to the brain regions utilized in the eQTL data and validate particular associations in subcortical brain regions by other means.* |
| --- | --- |
| Author Response | We thank the reviewer for this important follow-up comment. We fully agree that our previous defense on ‘common practice’ was insufficient in light of recent evidence.  We now make it clear in the manuscript that when cell type-specific *cis*-eQTLs from one brain region are used to study the phenotype from another brain region, the resulting associations should **not** be interpreted as evidence of region-matched regulatory effects. More broadly, the drug target MR literature has emphasized that MR estimates the effect of a genetically proxied perturbation and should not be over-interpreted as identifying a precise biological context, mechanism, or site of action^1^. In our setting, the main issue is less how the analysis is performed, and more how the results should be interpreted. Specifically, associations based on eQTLs from one brain tissue should not be interpreted as evidence that the effect acts locally in another unmatched brain region.  Following the reviewer’s recommendation, we revised the manuscript in three ways:  (1) We removed or rephrased statements that implied subcortical specificity or region-specific mechanistic inference.   - We removed this text in the previous revised manuscript, because it over-interpreted non-region-matched associations as region-specific findings:   “~~Since the literature has suggested the influence of region-specific inhibitory activity of neurons on risky behaviors [71], we further looked at the specific brain regions that significantly share cell type-specific causal eGenes with these risky behaviors. In particular, we found that these regions were generally involved in executive and visual functions, including fornix-stria terminalis (FXST), IFO, uncinate fasciculus (UNC), corona radiata, orbitofrontal cortex, isthmus cingulate, and ventral diencephalon [72-79]. Our results suggest a genetic link between risky behaviors and brain regions related to executive and visual functions in inhibitory neurons.~~”   - We removed all the subcortical region examples in **Results**.   For example (**Lines 485-489**): “Beyond these known links, we detected significant expression associations between several regional volumes and psychiatric disorders (FDR < 0.05, Spearman’s rank correlation test). The strongest was between major depressive disorder (MDD) and the left rostral middle frontal cortex (FDR = 4.01×10-45, Spearman’s rank correlation test; **S19 Fig**)~~, and SCZ and the accumbens (FDR = 0.004, Spearman's rank correlation test)~~.”   - We removed the following sentence and the corresponding figure (**former S8 Fig**):   “~~Coupled with the observed genetic link between risky behaviors and brain regions related to executive and visual functions in inhibitory neurons (~~**~~Fig. 3a~~** ~~and~~ **~~S8 Fig~~**~~), our results imply that the expression level of~~ *~~XKR6~~* ~~in inhibitory neurons may affect brain areas related to executive and visual functions; this potentially sensitizes individuals to external visual stimulation and increasing risk-tolerant willingness.~~”   - We also removed the following text and the corresponding figure (**former S18 Fig**):   “~~Additionally, we further explored the regional specificity of brain volume in different cell types using the causal eGenes, and our results showed that both subcortical and cortical regions exhibited stronger specificity to glial cells (~~**~~S18 Fig~~**~~). These results suggest that glial cells, such as astrocytes and microglia, have a significant impact on the volume of brain regions in adults [119, 120].~~”  (2) We added explicit text in **Results** and **Discussion** to state that non-region-matched associations should be interpreted cautiously.   - We added the following sentence at the end of subsection “Putative causal effects of eGenes on IDPs and DBs” in Results (**Lines 174-178**):   Collectively, these results nominate candidate cell type–specific regulators of brain structural phenotypes. Nevertheless, because the cis-eQTL data were derived from the prefrontal cortex, temporal cortex, and deep white matter—not all anatomical regions represented by the imaging outcomes—these findings should not be interpreted as pinpointing the precise brain region where each regulatory effect operates [42-44].   - We also added the following text to the subsection “Interpretation of cell type-specific associations” in **Discussion** (**Lines 563-571**):   It is also important to note that the cis-eQTL data used here were derived from a limited set of brain regions (prefrontal cortex, temporal cortex, and deep white matter) rather than the full complement of anatomical regions represented by the imaging phenotypes. Given the context dependence of molecular eQTL effects and known transcriptomic divergence across brain regions—especially between cortical and subcortical structures [44]—associations involving unmatched regions should be treated with caution. More broadly, MR captures the effect of a genetically proxied expression perturbation and does not identify the exact biological context or site of action [42]. Our results are therefore best regarded as prioritizing putative cell type–specific genes for brain phenotypes, rather than pinpointing the precise brain regions in which each regulatory effect occurs.  (3) We rewrote **Discussion** using more cautious language.   - In the “Shared genetic signals between risky behaviors and IDPs” subsection in **Discussion**, we replaced the previous brain region mechanism interpretation with the following more conservative text (**Lines 604-616**):   **Shared genetic signals between risky behaviors and IDPs**  Our analysis supports genetically driven links between risky behaviors and multiple IDPs at the cellular level. We observed significant sharing of causal eGenes within inhibitory neurons between risky behaviors (GRT and ASP) and both brain regional volumes and white matter microstructure phenotypes (**Fig. 3a**). Among these shared signals, XKR6 in inhibitory neurons was implicated in both risky behaviors (GRT and ASP) and several IDPs, including cortical volumetric phenotypes and white matter phenotypes such as the FXST, IFO and UNC. Using external expression data across cell types, we also detected significant expression-profile correlations between GRT and IFO, and between GRT and FXST (**S18 Fig**), pointing to a mediating role for cell type–specific causal eGenes in these associations. These results suggest a shared cell type–specific genetic architecture linking risky behaviors to selected IDPs. They should not, however, be taken as evidence for the precise brain regions in which these effects operate; that anatomical context awaits further validation.  We believe these revisions directly address the reviewer’s concern and clarify the interpretation of our results in light of current drug-target MR guidance^1^. |
| Excerpt 1.1 | (has been included in the above response) |

## REF 1.4 – Round2 - Correlational structure of IDPs

| *Referee Comment* | *Round2:*  *This comment has been largely addressed. However, I would like to see a scatterplot comparing IDP genetic correlation and overlapping causal eGenes, in case the association is not specifically captured by the top genetically correlated pair.*  *Round1* *recap:*  *Regarding the FDR correction, I would also be interested in understanding the correlation structure of the IDPs. Could the authors provide a supplementary figure detailing this? If it’s found that the correlational structure is interfering with the FDR correction, perhaps dimensionality reduction can be used to create representative categories of multiple IDPs? This is also especially critical to understand within class trait pleiotropy, as detailed in the section, "Pervasive trait pleiotropy of causal eGenes".* |
| --- | --- |
| Author Response | We thank the reviewer for this suggestion. We agree that a single extreme example was insufficient to show the overall pattern. In the revised manuscript, we therefore added a scatterplot comparing pairwise IDP genetic correlation and the number of overlapping causal eGenes across all 7,503 IDP pairs among the 123 IDPs (**S7b Fig**).  Across all 7,503 IDP pairs, we observed a statistically significant but weak positive association between the absolute pairwise genetic correlation (\|*rg*\| estimated by LDSC) and causal eGene overlap (Spearman’s *ρ* = 0.092, *P* = 1.95×10^-15^ when overlap was measured by raw count). We repeated the analysis using the Jaccard index, which expresses overlap as a proportion rather than a raw count, and obtained a very similar result (Spearman’s *ρ* = 0.089, *P* = 1.55×10^-14^).  We further examined this relationship separately by IDP pair type (i.e., between-group, within-BRV, and within-WMMP). The association remained weak for between-group pairs (Spearman’s *ρ* = 0.073, *P* = 6.10 × 10^-4^) and for within-BRV pairs (Spearman’s *ρ* = 0.063, *P* = 8.25 × 10^-6^), but was stronger for within-WMMP pairs (Spearman’s *ρ* = 0.602, *P* < 2.2 × 10^-16^). Overall, these results indicate that pairwise genetic correlation explains only a limited proportion of causal eGene overlap, particularly for between-group comparisons, which are the main focus of our study. We have revised the **Results** section accordingly.    **S7b Fig.** Relationship between pairwise genetic correlation and overlap of cell type–specific causal eGenes among IDPs. The x-axis shows absolute pairwise genetic correlation (\|rg\|), and the y-axis shows the number of overlapping cell type–specific causal eGenes identified by MR analysis. Each point represents one IDP pair, colored by pair type: between-group (one brain regional volume and one white matter microstructure phenotype), within-BRV (both brain regional volumes), or within-WMMP (both white matter microstructure phenotypes). The black curve shows a LOESS-smoothed trend, with the shaded area indicating the 95% confidence interval. |
| Excerpt 1.4 (Main Text) | **Lines 215-221 (Results):**  Notably, this pleiotropy was not fully explained by genetic correlations among IDPs. Across 7,503 IDP pairs, the absolute genetic correlation was only weakly associated with eGene overlap (Spearman’s rank correlation coefficient *r* = 0.092, *P* = 1.95×10^-15^; **S7b Fig**), and the association remained weak for between-group pairs (Spearman’s rank correlation coefficient *r* = 0.073, *P* = 6.10 × 10^-4^). Indeed, even the most strongly correlated between-group pair (PLIC_FA and right lateral ventricle, *r_g_* = 0.49) shared no causal eGenes. |

## REF 1.6 – Round2 - Clarifying the cell type specificity of eGene-trait pairs

| *Referee Comment* | *Round2:*  *Can the authors comment on the high degree of* *cell type specificity found via their analysis? While they cite literature with a consistent rate, the value seems to be much lower in other literature (PMID:* *35915177, for example). This can be explained by lower power in comparison to other studies or different statistical measures for specificity. This should be commented on in the discussion.*  *Round1 recap:*  *"Our predicted eGene-IDP pairs exhibited strong cell type specificity, with 91.4% (605 out of 662) existing only in a particular cell type, which is consistent with previous literature showing that the formation of brain structures is modulated by genes in a cell type-specific manner[37-39]." Can the authors clarify whether this rate is above what we would expect considering the underlying cell type specificity of the eQTLs themselves? Also, please clarify this for both IDP and DB analyses.* |
| --- | --- |
| Author Response | We thank the reviewer for this important comment. We agree that the reported cell type specificity rate in our study appears higher and warrants further discussion.  First, we would like to clarify that the rates being compared are not identical. In our study, the reported rates (91.4% for eGene-IDP pairs and 82.1% for eGene-DB pairs) refer to the **cell type specificity of significant eGene-trait associations** after TWAS preselection and multi-method MR filtering. In contrast, the study cited by the reviewer and a recent single-cell eQTL study reported lower specificity (~46-47%) at the level of **detected eGenes/eQTLs themselves**^2,3^. These are related but distinct measures: our results reflect the downstream specificity of genetically supported gene-trait associations, whereas the latter reflects the upstream specificity of gene regulation itself. Notably, the same study also found that, for most GWAS loci, only one gene colocalized in one cell type**^3^**. This supports the idea that downstream gene-trait associations may show higher cell type specificity than eQTL effects alone.  Second, as we showed in the previous revision, this high specificity is not only driven by genes being testable in only one cell type. When restricting to genes with valid *cis*-eQTL instruments in at least two cell types, a large proportion of associations still remained specific to a single cell type (80.6% for IDPs and 69.2% for DBs; **S4 Fig**).  Finally, we also totally agree that methodological factors may also contribute to the high specificity estimates in our study, including differences in how specificity is defined, the use of significance filtering (TWAS + MR), and unequal *cis*-eQTL availability across cell types (e.g. the lower number of *cis*-eQTLs in endothelial cells and pericytes). In the revised manuscript, we have added a paragraph in **Discussion** (**Lines 542-549**) to clarify these points and to interpret the observed specificity more cautiously. |
| Excerpt 1.6 (Main Text) | **Discussion (Lines 542-549):**  Interpretation of cell type-specific associations  The high cell type specificity observed here (91.4% for eGene–IDP pairs and 82.1% for eGene–DB pairs) should be interpreted cautiously. These rates reflect the specificity of significant eGene–phenotype associations after TWAS and MR filtering, not the inherent specificity of eQTL effects. This definitional distinction may partly explain why our estimates exceed those reported in studies focused on cell type–specific eGenes or eQTLs [13, 122]. In addition, methodological factors—such as significance filtering (TWAS + MR) and uneven *cis*-eQTL availability across cell types (e.g., the comparatively small number in endothelial cells and pericytes)—may inflate the apparent specificity. |

## REF 1.8 – Round2 - lack trait heritability in the text

| *Referee Comment* | *Round2: Mostly addressed. The authors provide an excerpt where not all traits are annotated with heritability (ALS, for example). The authors should describe why that is, or annotate them all with heritability.*  *Round1 recap:*  *As the article focuses on a multitude of traits with differing power, it would be useful to annotate the traits in the text with their heritability.* |
| --- | --- |
| Author Response | Thank you for this follow-up comment. For the three traits of ALS, ICH, and IS, the corresponding source papers did not provide a reported heritability estimate. We have therefore revised the **Methods** section (**Lines 696-697, 699-700, 702-703**) to indicate these cases explicitly as “not reported in the original GWAS publication,” rather than leaving them unannotated. |
| Excerpt 1.8 (Main Text) | **Lines 614-615, 617-618, 620-621 (Methods):**  (2) Amyotrophic lateral sclerosis (ALS): 20,806 cases and 59,804 controls (heritability: not reported in the original GWAS publication) [130]  (4) Intracerebral hemorrhage (ICH): 1,545 cases and 1,481 controls (heritability: not reported in the original GWAS publication) [132]  (6) Ischemic stroke (IS): 67,162 cases and 454,450 controls (heritability: not reported in the original GWAS publication) [134] |

# *Reviewer #2:*

*All my concerns have been properly addressed.*

**REF2 – Response to REF2:** We sincerely thank the reviewer for the positive feedback and for taking the time to review our work. We are pleased that our previous revisions have successfully addressed all the concerns.

# *Reviewer #3:*

*The authors addressed my main concerns. Some minor items remain, but they are very easily rectified, and I otherwise do not have additional concerns. Please note that most of my comments below relate to what appear to be incorrect cited references.*

**REF3 – Response to REF3:** Thank you for taking the time to review our revised paper, and we're pleased to hear that most of your concerns have been addressed. We hope that the remaining concerns are appropriately addressed in this round of revision. The detailed point-by-point responses are listed below.

**REF3 – Point to point response to REF3**

## REF 3.1 – Modification of sentence

| *Referee Comment* | *"Randomized controlled trials are considered as the gold standard for testing causality." --> "**Randomized controlled trials (RCTs) are considered to be the gold standard for testing causality."* |
| --- | --- |
| Author Response | Thanks for the suggestion. We have revised the sentence accordingly. |
| Excerpt 3.1 (Supplementary Text) | **S3 Text:**  Randomized controlled trials (RCTs) are considered to be the gold standard for testing causality. |

## REF 3.2 –Simplifying the description

| *Referee Comment* | *I would maybe devote one sentence to explaining what is meant by the word "exogenous" (ie, with respect to exogenous variation)*  *"Because genetic variants are fixed at conception and largely unaffected by environmental influences, they offer a source of exogenous variation that is less prone to confounding factors" --> "Because genetic variants are fixed at conception and largely unaffected by environmental influences, they offer a source of variation that is less prone to confounding factors"* |
| --- | --- |
| Author Response | Thanks for the comment. In the revised manuscript, we have modified the sentence to use more intuitive language and removed this technical word, which is now more accessible without any loss of meaning (**S3 Text**). |
| Excerpt 3.2 (Supplementary Text) | **S3 Text:**  Because genetic variants are fixed at conception and largely unaffected by environmental influences, they provide a source of variation that is naturally independent of confounding factors^5^. Therefore, if genetically predicted exposure variation is associated with an outcome, it supports a putative causal effect of the exposure on the outcome. |

## REF 3.3 – Issue of reference numbering system

| *Referee Comment* | *I would just ask the authors to double-check that all references are correctly cited within REF 3.1. My apologies if I'm mistaken, but some of the references seem like they might be mixed up. For example, the authors cite reference #5 several times (eg, "Because genetic variants ... confounding factors5", "As long as the genetic instruments ... biologically interpretable5."). I would just double-check to confirm that reference #5 (ie, Yang et al, 2022) is indeed the correct intended reference. Also, the authors cite Gandal et al (reference #13) when givein the approximated SE for the Wald ratio. Also, the authors cite reference #14 (Sollis et al, 2023) when stating that a proof has been given to show that 2SLS and IVW are asymptotically equivalent under ideal conditions.*  *These references (ie, references #36 and #37) are not correct in the following: "To confirm the reproducibility of the predicted eGene-IDP pairs and eGene-cell type-IDP triples, we repeated MR analyses using cortical eQTL summary statistics from BrainMeta [36] and cell type-specific eQTL summary statistics from brainSCOPE [37], respectively."*  *I think these references should perhaps also be changed: "As we did for the IDPs, we confirmed the reproducibility of eGene-DB pairs using three datasets: the cortical eQTL summary statistics from BrainMeta [36], differentially expressed genes and transcripts from PsychENCODE [29]"*  *Also this reference #37: "was generated using single cell-resolution data from 388 adult DLPFC samples [37]"* |
| --- | --- |
| Author Response | We sincerely apologize for the confusion regarding the citations. This happened because the numbers in our "Excerpts" (the copied parts of the paper) in *Response to Reviewers* letter were copied directly from the **Main Text** or **Supplementary Text** to keep them the same as the actual paper. However, these numbers did not match the separate list at the end of the Response Letter.  For example,  **1. In the Supplementary Text (S3 Text):**  Reference #5: This is *Sanderson et al. (2022)*, the 5^th^ literature in the Reference section in Supplementary Text ^4^. It is a textbook-style paper that explains the basic rules and ideas of MR.  Reference #13: This is *Thomas et al. (2007)*, the 13^th^ literature in the Reference section in Supplementary Text ^5^. This paper provides the math formula we used to calculate the approximated standard error for the Wald ratio.  Reference #14: This is *Burgess et al. (2017)*, the 14^th^ literature in the Reference section in Supplementary Text ^6^. This paper proves that the two statistical methods (2SLS and IVW) are asymptotically equivalent under ideal conditions.  **2. In the Main Text:**  Reference #29: This is *Gandal et al. (2018)*, the 29^th^ literature in Reference section in Main Text ^7^. This paper differentially expressed genes and transcripts from PsychENCODE.  Reference #36: This is *Qi et al. (2022)*, the 36^th^ literature in Reference section in Main Text ^8^*.* This paper provided the online tool of BrainMeta which provided cortical eQTL summary statistics.  Reference #37: This is *Emani et al. (2024)*, the 37^th^ literature in Reference section in Main Text ^9^. This paper provided brainSCOPE resource which provided cell type-specific eQTL summary statistics based on 388 human brain samples.  To ensure complete clarity, we have updated this *Response to Reviewers* letter to explicitly distinguish between the two separate reference numbering system at Page 1: “Reference numbers appearing within quoted manuscript excerpts follow the numbering in the Revised Manuscript, whereas references cited in our response text use a separate numbering system”. |

# References

1	Gill, D. *et al.* Common pitfalls in drug target Mendelian randomization and how to avoid them. *BMC Med* **22**, 473, doi:10.1186/s12916-024-03700-9 (2024).

2	Yazar, S. *et al.* Single-cell eQTL mapping identifies cell type-specific genetic control of autoimmune disease. *Science (New York, N.Y.)* **376**, eabf3041, doi:10.1126/science.abf3041 (2022).

3	Bryois, J. *et al.* Cell-type-specific cis-eQTLs in eight human brain cell types identify novel risk genes for psychiatric and neurological disorders. *Nature neuroscience* **25**, 1104-1112 (2022).

4	Sanderson, E. *et al.* Mendelian randomization. *Nat Rev Methods Primers* **2**, doi:10.1038/s43586-021-00092-5 (2022).

5	Thomas, D. C., Lawlor, D. A. & Thompson, J. R. Re: Estimation of bias in nongenetic observational studies using" Mendelian triangulation" by Bautista et al. (2007).

6	Burgess, S., Zuber, V., Valdes-Marquez, E., Sun, B. B. & Hopewell, J. C. Mendelian randomization with fine-mapped genetic data: Choosing from large numbers of correlated instrumental variables. *Genet Epidemiol* **41**, 714-725, doi:10.1002/gepi.22077 (2017).

7	Gandal, M. J. *et al.* Transcriptome-wide isoform-level dysregulation in ASD, schizophrenia, and bipolar disorder. *Science (New York, N.Y.)* **362**, eaat8127 (2018).

8	Qi, T. *et al.* Genetic control of RNA splicing and its distinct role in complex trait variation. *Nat Genet* **54**, 1355-1363, doi:10.1038/s41588-022-01154-4 (2022).

9	Emani, P. S. *et al.* Single-cell genomics and regulatory networks for 388 human brains. *Science (New York, N.Y.)* **384**, eadi5199, doi:10.1126/science.adi5199 (2024).
