# Supplementary material for: Deciphering cell type-specific causal genetic effects on brain imaging-derived phenotypes and disorders with single-cell Mendelian randomization
Source: PLoS Comput Biol. 2026 Jun 17;22(6):e1014422. doi: 10.1371/journal.pcbi.1014422 (PMC13289931; doi:10.1371/journal.pcbi.1014422)
Supplement: Peer review history

# Author comment: Author response to Decision Letter 0

**DOI:** 10.1371/journal.pcbi.1014422.r001

**Transfer Alert**

This paper was transferred from another journal. As a result, its full editorial history (including decision letters, peer reviews and author responses) may not be present.

16 May 2025

# Peer review: Decision Letter 0

**DOI:** 10.1371/journal.pcbi.1014422.r002

**Authors:** Daifeng Wang, Ferhat Ay

7 Oct 2025

PCOMPBIOL-D-25-00949

Deciphering cell type-specific causal genetic effects on brain imaging-derived phenotypes and disorders with single-cell Mendelian randomization

PLOS Computational Biology

Dear Dr. Zhao,

Thank you for submitting your manuscript to PLOS Computational Biology. After careful consideration, we feel that it has merit but does not fully meet PLOS Computational Biology's publication criteria as it currently stands. Therefore, we invite you to submit a revised version of the manuscript that addresses the points raised during the review process.

Please submit your revised manuscript within 60 days Dec 07 2025 11:59PM. If you will need more time than this to complete your revisions, please reply to this message or contact the journal office at ploscompbiol@plos.org. When you're ready to submit your revision, log on to https://www.editorialmanager.com/pcompbiol/ and select the 'Submissions Needing Revision' folder to locate your manuscript file.

Please include the following items when submitting your revised manuscript:

* A rebuttal letter that responds to each point raised by the editor and reviewer(s). You should upload this letter as a separate file labeled 'Response to Reviewers'. This file does not need to include responses to formatting updates and technical items listed in the 'Journal Requirements' section below.

* A marked-up copy of your manuscript that highlights changes made to the original version. You should upload this as a separate file labeled 'Revised Manuscript with Track Changes'.

* An unmarked version of your revised paper without tracked changes. You should upload this as a separate file labeled 'Manuscript'.

If you would like to make changes to your financial disclosure, competing interests statement, or data availability statement, please make these updates within the submission form at the time of resubmission. Guidelines for resubmitting your figure files are available below the reviewer comments at the end of this letter

We look forward to receiving your revised manuscript.

Kind regards,

Ferhat Ay, Ph.D

Section Editor

PLOS Computational Biology

Ferhat Ay

Section Editor

PLOS Computational Biology

**Journal Requirements:**

If the reviewer comments include a recommendation to cite specific previously published works, please review and evaluate these publications to determine whether they are relevant and should be cited. There is no requirement to cite these works unless the editor has indicated otherwise.

1) Please provide an Author Summary. This should appear in your manuscript between the Abstract (if applicable) and the Introduction, and should be 150-200 words long. The aim should be to make your findings accessible to a wide audience that includes both scientists and non-scientists. Sample summaries can be found on our website under Submission Guidelines:

https://journals.plos.org/ploscompbiol/s/submission-guidelines#loc-parts-of-a-submission

2) We do not publish any copyright or trademark symbols that usually accompany proprietary names, eg ©,  ®, or TM  (e.g. next to drug or reagent names). Therefore please remove all instances of trademark/copyright symbols throughout the text, including:

- © on page: 50.

3) Please upload all main figures as separate Figure files in .tif or .eps format. For more information about how to convert and format your figure files please see our guidelines:

https://journals.plos.org/ploscompbiol/s/figures

4) We have noticed that you have uploaded Supporting Information files, but you have not included a list of legends. Please add a full list of legends for your Supporting Information files after the references list.

5) Some material included in your submission may be copyrighted. According to PLOSu2019s copyright policy, authors who use figures or other material (e.g., graphics, clipart, maps) from another author or copyright holder must demonstrate or obtain permission to publish this material under the Creative Commons Attribution 4.0 International (CC BY 4.0) License used by PLOS journals. Please closely review the details of PLOSu2019s copyright requirements here: PLOS Licenses and Copyright. If you need to request permissions from a copyright holder, you may use PLOS's Copyright Content Permission form.

Please respond directly to this email and provide any known details concerning your material's license terms and permissions required for reuse, even if you have not yet obtained copyright permissions or are unsure of your material's copyright compatibility. Once you have responded and addressed all other outstanding technical requirements, you may resubmit your manuscript within Editorial Manager.

Potential Copyright Issues:

i) Figures 1A, and S1. Please confirm whether you drew the images / clip-art within the figure panels by hand. If you did not draw the images, please provide (a) a link to the source of the images or icons and their license / terms of use; or (b) written permission from the copyright holder to publish the images or icons under our CC BY 4.0 license. Alternatively, you may replace the images with open source alternatives. See these open source resources you may use to replace images / clip-art:

- https://commons.wikimedia.org

- https://openclipart.org/.

6) Please amend your detailed Financial Disclosure statement. This is published with the article. It must therefore be completed in full sentences and contain the exact wording you wish to be published.

1) State what role the funders took in the study. If the funders had no role in your study, please state: "The funders had no role in study design, data collection and analysis, decision to publish, or preparation of the manuscript."

2) If any authors received a salary from any of your funders, please state which authors and which funders..

If you did not receive any funding for this study, please simply state: u201cThe authors received no specific funding for this work.u201d

**Reviewers' comments:**

Reviewer's Responses to Questions

**Comments to the Authors:**

**Please note here if the review is uploaded as an attachment.**

Reviewer #1: Summary: Overall, a very interesting article with findings pertinent to the field. While I have some analytical concerns, I believe these can be addressed, and overall, I recommend this article to be accepted with minor revisions.

1. One potential flaw in the analytical workflow is that the authors use cell type-specific eQTLs from prefrontal cortex, temporal cortex, and deep white matter to infer associations about other brain regions. The literature on whether this is appropriate is currently in flux. Currently the data suggests that the same cell type in different cortical regions in generally consistent (PMID: 39578476), but this is not necessarily the case subcortically (PMID: 37824663). Since there are few resources for single-cell data for most brain regions (excluding the dorsolateral prefrontal cortex), I recommend the authors limit their broader analyses to the brain regions utilized in the eQTL data and validate particular associations in subcortical brain regions by other means.

2. Regarding figure 1B., it is difficult to visually identify between 0 and 1 eGenes according to the color scale. I recommend using a distinct color for 0 and then using a color bar to annotate between 1 and the maximum value.

3. "As shown in Fig. 1b and S4 Table, we identified 254 eGenes that may have causal effects on 112 IDPs in eight cell types (FDR < 0.05)..." Can the authors clarify how FDR correction was applied? I would apply it across all IDPs+DBs and cell types.

4. Regarding the FDR correction, I would also be interested in understanding the correlation structure of the IDPs. Could the authors provide a supplementary figure detailing this? If it’s found that the correlational structure is interfering with the FDR correction, perhaps dimensionality reduction can be used to create representative categories of multiple IDPs? This is also especially critical to understand within class trait pleiotropy, as detailed in the section, "Pervasive trait pleiotropy of causal eGenes".

5. "found that 76.3% (505 out of 662; P < 10-16, hypergeometric test) of the eGene-IDP pairs could be successfully replicated ..." It would be helpful to state that this replication is at a nominal level. Additionally, if any of the methods provide directionality, it would be helpful to confirm the directionality matches.

6. "Our predicted eGene-IDP pairs exhibited strong cell type specificity, with 91.4% (605 out of 662) existing only in a particular cell type, which is consistent with previous literature showing that the formation of brain structures is modulated by genes in a cell type-specific manner[37-39]." Can the authors clarify whether this rate is above what we would expect considering the underlying cell type specificity of the eQTLs themselves? Also, please clarify this for both IDP and DB analyses.

7. "In contrast, the causal eGenes shared within the brain regional volume group were generally involved in protein binding, such as Hsp90 protein binding and MHC class I protein binding (Fig. 2c and S7 Table), which aligns with previous findings showing that the expression of Hsp90 and MHC class I proteins could regulate synapse formation and brain development[55, 56]." Could the authors clarify whether SNPs within the MHC were used to identify eGenes? The high linkage disequilibrium of this region requires careful analysis to make sure genetic associations are not spurious.

8. As the article focuses on a multitude of traits with differing power, it would be useful to annotate the traits in the text with their heritability.

9. For analyses comparing between and within DBs and IDPs, can the authors clarify if there is sample overlap between the compared GWASs? If there is, can overlapping samples be excluded, or accounted for with external replication of findings?

10. Regarding the TWAS analysis, could the authors clarify the filtering used for gene imputation models? While there is some diversity in the literature, a common threshold is CV R2 >= 0.01 and CV q <= 0.05. While the threshold used as an initial filter was TWAS p <= 0.05, it is important to note how imputable those genes are in the first place.

11. In Figure 4, is there any logic for the Sankey chart crossovers (for example, there is a crossover from "Astrocytes" to "MAPT" and "ZSCAN31"? Or is that just an aesthetic choice? I would remove this if this is purely aesthetic, and if it is not, please make sure it is clearly labeled in the legend.

12. Please clear up the labelling in figure 5A's legend. The shading to indicate prenatal vs postnatal is unintuitive. This could be corrected by applying patterning to indicate prenatal vs postanal, or fully explaining each color in the legend, rather than explaining 2 colors and 2 shades.

13. The y-axis labeling in figure 6 seems to be low resolution compared to the rest of the text.

Reviewer #2: This manuscript by Yang et al is a very dense and interesting paper, connecting brain cell-type–specific cis-eQTLs with imaging-derived phenotypes (IDPs) and diseases/behaviors (DBs). The authors report catalogs of causal eGenes and emphasize extensive cell-type specificity (e.g., ~91% of eGene–IDP pairs in only one cell type). The direction is timely and potentially impactful. However, the central claim—cell-type–specific causality—remains insufficiently validated. Replication analyses largely eGene–trait concordance, not cell-type attribution; single-cell QC is under-specified; reliance on an external eQTL map is not audited; and several statistics (e.g., “91.4% unique”) are likely inflated by input data bias, power asymmetries, and thresholding.

1) Replication does not test cell-type specificity, while their core claim is about “cell type-specific causal genetic effects”. The current “reproducibility” checks (BrainMeta tissue-level eQTLs, PsychENCODE DE, GWAS Catalog) validate gene-to-trait links, not which cell type is causal. Please consider:

• It is concerning when the cell-type specificity cannot be validated. Why not use larger single-cell eQTL resources (e.g., Fujita et al. 2024; Mathys et al. 2023)?

• In the replication test, direction concordance and adjust P-values in the replication stage should be reported.

• If the cell-type specificity cannot be confirmed, the newly discovered eGenes and their associated IDPs, DBs are still interesting. But it is important to be certain.

2) Similarly, “91.4% unique to one cell type (for eGene-IDP)” is likely inflated by bias/thresholding, and might be misleading without validating the cell-type specificity. The 91.4% can easily arise from unequal eQTL effect size, underpowered samples, instrument availability/strength, QC differences, and per–cell-type thresholding.

Figure 3 indicates that SCZ and BD have very distinct cell type composition regarding eGenes, and also share a few eGenes. This is contradictory to the common knowledge that SCZ and BD share significant genetics. I suspect the inflated cell-type-specificity is the explanation.

3) Reliance on an external cell-type eQTL resource might be a concern. Your eQTL and sc gene expression data all come entirely from a prior publication. You may need to audit those data and show that their QC/procedures do not manufacture specificity. It is particularly a concern for scRNA-seq data. Given high noise and dropout in snRNA-seq, QC will likely affect result consistency, such as Ambient RNA decontamination (e.g., SoupX/DecontX/other): tool/versions/parameters; marker gene uses; missingness/dropout filters: per-cell thresholds (min genes/UMIs, max mito%, doublet score, missing-rate), per-gene thresholds (min expressing-cell fraction within a cell type); noise level in the data, etc.

5) Systematically map DB–IDP relationships through cell-type and SNPs. Current sharing/overlap is primarily at the gene level. To deliver the promise on “bridging cell type/region/behavior” additional analyses might be useful.

• Build a phenotype × phenotype similarity matrix using cell-type–resolved overlap (Jaccard/overlap, optionally IV-weighted) of causal eGenes within cell types, then aggregate across cell types. Cluster phenotypes; report cluster stability (bootstrap), enrichment of trait classes within clusters, and which cell types drive cohesion.

• Variant-resolved extension is recommended: fine-map eQTLs per cell type and GWAS loci; perform variant-level colocalization; repeat the clustering using shared functional SNPs (more specific than genes). Annotate SNPs to cell-type/region enhancers and TF motifs to provide a mechanistic chain (SNP→element→gene→cell type→brain region→phenotype). Include a short list of sentinel variants per cluster suitable for MPRA/CRISPRi/a follow-up.

7) Bidirectional IDP↔DB effects could be quantified and classified. Don’t drop it. Pairs showing bidirectional MR were excluded “for clarity” in current manuscript, but they are informative.

8) “Genetic constraint of shared causal eGenes” is confounded as currently framed. The claim that DB-implicated genes are “more conserved” than IDP-implicated genes is difficult to interpret due to class breadth, power asymmetry, and eGene detectability biases (highly constrained genes often lack strong common cis-eQTLs).

9) “Mutually causal” might be a misused phrase where pleiotropy/overlap across traits might be intended. Reserve “mutual/bidirectional” for true A↔B MR that passes predefined criteria.

11) Lines 180–190: The authors report excitatory and inhibitory neurons as predominant cell types, even for AD. This contrasts with Mathys et al. 2023, which emphasized microglia. How do the authors explain this inconsistency?

12) Lines 417–421: The authors report higher expression of causal eGenes in glial cells, yet most causal genes were identified in neurons. How can this be reconciled?

13) Figure 5: Progenitor cells are not included in the prenatal data. Would that be a missed opportunity?

14) For key gene–trait–cell-type triplets, focus on sign-consistency chains (eQTL allele → expression → MR effect on trait), and explain inconsistent chains.

Reviewer #3: The authors present a careful set of analyses and results aimed at delineating causal relationships between the eGene expression in specific cell types and (1) brain disorders and behaviors (mostly psychiatric disorders) and (2) image-derived phenotypes. To do so, they used cis-eQTLs derived in 8 brain cell types (previously published by Bryois et al) and published results from GWAS/TWAS studies. Causal inference between eGenes and disorders/phenotypes were performed using the machinery behind Mendelian randomization, with a key novel hallmark of this study being the fact that MR was performed at the level of distinct cell types (in contrast to previous studies of this general nature). The authors identified hundreds of candidate causal genes for dozens of different brain-related traits of biomedical interest. Further downstream analyses of the causal eGenes was performed to investigate pleiotropy, and they also evaluate the potential causal routes at play. Spatiotemporal expression data from the identified genes were used to examine links between the studied phenotypes and expression.

Major comments:

The most important limitation in this study is not in the narrative, results, and analyses, but rather in the exposition and delineation of the underlying principles underlying MR and causal inference. To be more specific -- although the authors could refer to published reviews, textbooks, protocols etc to explain the mechanics of how Mendelian randomization works (for ex - see ref: Sanderson, Eleanor, M. Maria Glymour, Michael V. Holmes, Hyunseung Kang, Jean Morrison, Marcus R. Munafò, Tom Palmer et al. "Mendelian randomization." Nature reviews Methods primers 2, no. 1 (2022): 6.), I feel that the paper would be greatly improved if the authors provided a dedicated section to explain the principles behind MR more clearly (this section could just consist of a few paragraphs and a few schematics, and it can even be placed in in the Supplement if needed). Although a small schematic does appear within a sub-panel of Fig. 1a(ii), a fuller and clearer explanation would substantially boost the accessibility of this manuscript on the whole.

On that note, the authors can include 1 or 2 paragraphs (also in the supplement) to explain the underlying theory behind causal inference -- for example: the machinery governing how one can infer causality routes among cell type-specific the causal eGenes, DBs, and IDPs. These explanations would not need to be overly formal or even mathematically dense. It's the intuition that is needed (I'd also ask the authors to emphasize the importance of potential confounders in this type of analysis)

It is not clear why the inclusion of data from UK Biobank would entail removing samples (eg, see lines 568-569).

The Bryois et al dataset contains scQTLs in 8 cell types from 192 individuals. But the Emani et al data contains scQTLs in >15 cell types from a starting dataset size 388 individuals, which would presumably boost both statistical power and biological resolution (with more annotated cell types). If the authors decide to keep the Bryois set of QTLs (which admittedly has the advantage of a more interpretable story with more broadly-defined cell types), then they can perhaps point to the Emani data as an avenue for future/extended studies of this nature. -- ref: Emani, Prashant S., et al. "Single-cell genomics and regulatory networks for 388 human brains." Science 384.6698 (2024): eadi5199.

Throughout -- the language "cell type-specific eGenes" and "cell type-specific causal eGenes" is ambiguous. In some of the literature, the term "cell type-specific eGenes" denotes eGenes that show up exclusively in one cell type only (and not any others) -- whereas elsewhere, the term may be more loosely defined as showing up in a small number of cell types (but more than one). In yet other published work, the term is very loosely defined to sipmly mean eGenes that are identified using snRNAseq data -- even if the eGene shows up as significant in a large number of cell types.

Regarding the results on cell type-specific causal eGenes that are common between psychiatric disorders (for example, the results showcased in Fig 3b) -- an analysis (or at least a discussion) regarding comorbidity between the different psychiatric disorders may really add value. The logic here is that if two disorders share many causal eGenes, one may expect that this pair of disorders exhibits high comorbidity. Do the authors observe this to be the case (of course, this is not guaranteed to be the case -- shared causal eGenes would imply comorbidity of the eSNPs are also shared). But the authors may be able to check for this without having to carry out the comorbidity analysis themselves -- instead, catalogs of pairwise comorbidity can probably just be checked in recent studies. Many of those results should be available in the literature -- for just handful of examples, maybe see:

- McGrath et al., 2020 — “Comorbidity within mental disorders.” World Psychiatry. : https://pmc.ncbi.nlm.nih.gov/articles/PMC7443806/?utm_source=chatgpt.com

- Bourque et al., 2024 — “Genetic and phenotypic similarity across major psychiatric disorders.” Molecular Psychiatry (systematic review). : https://www.nature.com/articles/s41398-024-02866-3?utm_source=chatgpt.com

- Solmi et al., 2023 — “Meta-analytic prevalence of comorbid mental disorders in clinical high-risk for psychosis (CHR-P).” Molecular Psychiatry (meta-analysis). : https://www.nature.com/articles/s41380-023-02029-8?utm_source=chatgpt.com

- Kessler et al., 2005 — NCS-R 12-month prevalence & comorbidity (JAMA Psychiatry). -- https://jamanetwork.com/journals/jamapsychiatry/fullarticle/208671?utm_source=chatgpt.com

Maybe see also:

- Platona, Rita Ioana, et al. "The impact of psychiatric comorbidities associated with depression: a literature review." Medicine and pharmacy reports 97.2 (2024): 143.

- Lee, Sool, et al. "Massively parallel reporter assay investigates shared genetic variants of eight psychiatric disorders." Cell 188.5 (2025): 1409-1424.

- Raznahan, Armin, et al. "Convergence and divergence of rare genetic disorders on brain phenotypes: a review." JAMA psychiatry 79.8 (2022): 818-828.

Minor comments:

"Julien et al[12]" --> "Bryois et al[12]" (Julien is this author's first name; Bryois is the last name)

For GWAS -- maybe just cite: Uffelmann, Emil, et al. "Genome-wide association studies." Nature Reviews Methods Primers 1.1 (2021): 59.

The figure resolutions are not ideal (but this could potentially be because of the review-stage PDF)

"an analytic framework for causality identification" --> "an analytic framework for causal inference"

line 50-52: w.r.t. "It has been revealed that cell type-level cis-eQTLs could have larger effect sizes and target more constrained genes than tissue-level cis-eQTLs[12]." --> By "constrained", are the authors referring to evolutionary constraint?

"There is still a long road lays ahead in early treatment" --> "There is still a long road that lays ahead in early treatment"

**********

**Have the authors made all data and (if applicable) computational code underlying the findings in their manuscript fully available?**

The PLOS Data policy requires authors to make all data and code underlying the findings described in their manuscript fully available without restriction, with rare exception (please refer to the Data Availability Statement in the manuscript PDF file). The data and code should be provided as part of the manuscript or its supporting information, or deposited to a public repository. For example, in addition to summary statistics, the data points behind means, medians and variance measures should be available. If there are restrictions on publicly sharing data or code —e.g. participant privacy or use of data from a third party—those must be specified.

Reviewer #1: Yes

Reviewer #2: Yes

Reviewer #3: Yes

**********

PLOS authors have the option to publish the peer review history of their article (what does this mean?). If published, this will include your full peer review and any attached files.

If you choose “no”, your identity will remain anonymous but your review may still be made public.

**Do you want your identity to be public for this peer review?**       For information about this choice, including consent withdrawal, please see our Privacy Policy.

Reviewer #1: No

Reviewer #2: No

Reviewer #3: No

**Figure resubmission:**

While revising your submission, we strongly recommend that you use PLOS’s NAAS tool (https://ngplosjournals.pagemajik.ai/artanalysis) to test your figure files. NAAS can convert your figure files to the TIFF file type and meet basic requirements (such as print size, resolution), or provide you with a report on issues that do not meet our requirements and that NAAS cannot fix.

After uploading your figures to PLOS’s NAAS tool - https://ngplosjournals.pagemajik.ai/artanalysis, NAAS will process the files provided and display the results in the "Uploaded Files" section of the page as the processing is complete. If the uploaded figures meet our requirements (or NAAS is able to fix the files to meet our requirements), the figure will be marked as "fixed" above. If NAAS is unable to fix the files, a red "failed" label will appear above. When NAAS has confirmed that the figure files meet our requirements, please download the file via the download option, and include these NAAS processed figure files when submitting your revised manuscript.

**Reproducibility:**

To enhance the reproducibility of your results, we recommend that authors of applicable studies deposit laboratory protocols in protocols.io, where a protocol can be assigned its own identifier (DOI) such that it can be cited independently in the future. Additionally, PLOS ONE offers an option to publish peer-reviewed clinical study protocols. Read more information on sharing protocols at https://plos.org/protocols?utm_medium=editorial-email&utm_source=authorletters&utm_campaign=protocols

# Author comment: Author response to Decision Letter 1

**DOI:** 10.1371/journal.pcbi.1014422.r003

17 Feb 2026

Attachment Submitted filename: Response to Reviewers.docx

# Peer review: Decision Letter 1

**DOI:** 10.1371/journal.pcbi.1014422.r004

**Authors:** Daifeng Wang, Ferhat Ay, Daifeng Wang, Ferhat Ay

18 Mar 2026

PCOMPBIOL-D-25-00949R1

Deciphering cell type-specific causal genetic effects on brain imaging-derived phenotypes and disorders with single-cell Mendelian randomization

PLOS Computational Biology

Dear Dr. Zhao,

Thank you for submitting your manuscript to PLOS Computational Biology. After careful consideration, we feel that it has merit but does not fully meet PLOS Computational Biology's publication criteria as it currently stands. Therefore, we invite you to submit a revised version of the manuscript that addresses the points raised during the review process.

Please submit your revised manuscript by May 18 2026 11:59PM. If you will need more time than this to complete your revisions, please reply to this message or contact the journal office at ploscompbiol@plos.org. When you're ready to submit your revision, log on to https://www.editorialmanager.com/pcompbiol/ and select the 'Submissions Needing Revision' folder to locate your manuscript file.

Please include the following items when submitting your revised manuscript:

* A letter that responds to each point raised by the editor and reviewer(s). You should upload this letter as a separate file labeled 'Response to Reviewers'. This file does not need to include responses to formatting updates and technical items listed in the 'Journal Requirements' section below.

* A marked-up copy of your manuscript that highlights changes made to the original version. You should upload this as a separate file labeled 'Revised Manuscript with Track Changes'.

* An unmarked version of your revised paper without tracked changes. You should upload this as a separate file labeled 'Manuscript'.

If you would like to make changes to your financial disclosure, competing interests statement, or data availability statement, please make these updates within the submission form at the time of resubmission. Guidelines for resubmitting your figure files are available below the reviewer comments at the end of this letter.

We look forward to receiving your revised manuscript.

Kind regards,

Daifeng Wang, Ph.D.

Academic Editor

PLOS Computational Biology

Ferhat Ay

Section Editor

PLOS Computational Biology

**Reviewers' comments:**

Reviewer's Responses to Questions

**Comments to the Authors:**

**Please note here if the review is uploaded as an attachment.**

Reviewer #1: Summary: The authors address many of the concerns of the paper, though statistical issues in their approach persist. I do believe that these issues can be addressed largely through textual revisions and careful statement of these concerns throughout the text.

1. The authors defend their use of eQTLs from one brain region by stating that this approach is (1) defensible via the assumptions of mendelian randomization, and (2) is common in MR, TWAS, and colocalization literature. (1) Examination of their mendelian randomization framework reveals potential issues in the independence assumption via this approach, as the association between eQTLs in tissue 1 and phenotype in tissue 2 will be confounded by the potential eQTL in tissue 2. (2) Moreover, while this approach has been somewhat common in the past, time has revealed multiple issues in this paradigm (PMID: 39407214, 35113864). As a result, I recommend that the authors either remove these off-target associations from their study, or describe the above issues in the results and discussion.

2. Addressed

3. Addressed

4. This comment has been largely addressed. However, I would like to see a scatterplot comparing IDP genetic correlation and overlapping causal eGenes, in case the association is not specifically captured by the top genetically correlated pair.

5. Addressed

6. Can the authors comment on the high degree of cell type specificity found via their analysis? While they cite literature with a consistent rate, the value seems to be much lower in other literature (PMID: 35915177, for example). This can be explained by lower power in comparison to other studies or different statistical measures for specificity. This should be commented on in the discussion.

7. Addressed

8. Mostly addressed. The authors provide an excerpt where not all traits are annotated with heritability (ALS, for example). The authors should describe why that is, or annotate them all with heritability.

9. Addressed

10. Addressed

11. Addressed

12. Addressed

13. Addressed

Reviewer #2: All my concerns have been properly addressed.

Reviewer #3: The authors addressed my main concerns. Some minor items remain, but they are very easily rectified, and I otherwise do not have additional concerns. Please note that most of my comments below relate to what appear to be incorrect cited references.

"Randomized controlled trials are considered as the gold standard for testing causality." --> "Randomized controlled trials (RCTs) are considered to be the gold standard for testing causality."

I would maybe devote one sentence to explaining what is meant by the word "exogenous" (ie, with respect to exogenous variation)

"Because genetic variants are fixed at conception and largely unaffected by environmental influences, they offer a source of exogenous variation that is less prone to confounding factors" --> "Because genetic variants are fixed at conception and largely unaffected by environmental influences, they offer a source of variation that is less prone to confounding factors"

I would just ask the authors to double-check that all references are correctly cited within REF 3.1. My apologies if I'm mistaken, but some of the references seem like they might be mixed up. For example, the authors cite reference #5 several times (eg, "Because genetic variants ... confounding factors5", "As long as the genetic instruments ... biologically interpretable5."). I would just double-check to confirm that reference #5 (ie, Yang et al, 2022) is indeed the correct intended reference. Also, the authors cite Gandal et al (reference #13) when givein the approximated SE for the Wald ratio. Also, the authors cite reference #14 (Sollis et al, 2023) when stating that a proof has been given to show that 2SLS and IVW are asymptotically equivalent under ideal conditions.

These references (ie, references #36 and #37) are are not correct in the following: "To confirm the reproducibility of the predicted eGene-IDP pairs and eGene-cell type-IDP triples, we repeated MR analyses using cortical eQTL summary statistics from BrainMeta [36] and cell type-specific eQTL summary statistics from brainSCOPE [37], respectively."

I think these references should perhaps also be changed: "As we did for the IDPs, we confirmed the reproducibility of eGene-DB pairs using three datasets: the cortical eQTL summary statistics from BrainMeta [36], differentially expressed genes and transcripts from PsychENCODE [29]"

Also this reference #37: "was generated using single cell-resolution data from 388 adult DLPFC samples [37]"

**********

**Have the authors made all data and (if applicable) computational code underlying the findings in their manuscript fully available?**

The PLOS Data policy requires authors to make all data and code underlying the findings described in their manuscript fully available without restriction, with rare exception (please refer to the Data Availability Statement in the manuscript PDF file). The data and code should be provided as part of the manuscript or its supporting information, or deposited to a public repository. For example, in addition to summary statistics, the data points behind means, medians and variance measures should be available. If there are restrictions on publicly sharing data or code —e.g. participant privacy or use of data from a third party—those must be specified.

Reviewer #1: Yes

Reviewer #2: None

Reviewer #3: Yes

**********

PLOS authors have the option to publish the peer review history of their article (what does this mean?). If published, this will include your full peer review and any attached files.

If you choose “no”, your identity will remain anonymous but your review may still be made public.

**Do you want your identity to be public for this peer review?**       For information about this choice, including consent withdrawal, please see our Privacy Policy.

Reviewer #1: No

Reviewer #2: **Yes:**     Chunyu Liu

Reviewer #3: No

[NOTE: If reviewer comments were submitted as an attachment file, they will be attached to this email and accessible via the submission site. Please log into your account, locate the manuscript record, and check for the action link "View Attachments". If this link does not appear, there are no attachment files.]

**Figure resubmission:**

-->While revising your submission, we strongly recommend that you use PLOS’s NAAS tool (https://ngplosjournals.pagemajik.ai/artanalysis) to test your figure files. NAAS can convert your figure files to the TIFF file type and meet basic requirements (such as print size, resolution), or provide you with a report on issues that do not meet our requirements and that NAAS cannot fix.

After uploading your figures to PLOS’s NAAS tool - https://ngplosjournals.pagemajik.ai/artanalysis, NAAS will process the files provided and display the results in the "Uploaded Files" section of the page as the processing is complete. If the uploaded figures meet our requirements (or NAAS is able to fix the files to meet our requirements), the figure will be marked as "fixed" above. If NAAS is unable to fix the files, a red "failed" label will appear above. When NAAS has confirmed that the figure files meet our requirements, please download the file via the download option, and include these NAAS processed figure files when submitting your revised manuscript.

**Reproducibility:**

To enhance the reproducibility of your results, we recommend that authors of applicable studies deposit laboratory protocols in protocols.io, where a protocol can be assigned its own identifier (DOI) such that it can be cited independently in the future. Additionally, PLOS ONE offers an option to publish peer-reviewed clinical study protocols. Read more information on sharing protocols at https://plos.org/protocols?utm_medium=editorial-email&utm_source=authorletters&utm_campaign=protocols

# Author comment: Author response to Decision Letter 2

**DOI:** 10.1371/journal.pcbi.1014422.r005

15 May 2026

Attachment Submitted filename: Response_to_reviewers.docx

# Peer review: Decision Letter 2

**DOI:** 10.1371/journal.pcbi.1014422.r006

**Authors:** Daifeng Wang, Ferhat Ay, Daifeng Wang, Ferhat Ay, Daifeng Wang, Can Yang

7 Jun 2026

Dear Professor Yang,

We are pleased to inform you that your manuscript 'Deciphering cell type-specific causal genetic effects on brain imaging-derived phenotypes and disorders with single-cell Mendelian randomization' has been provisionally accepted for publication in PLOS Computational Biology.

Before your manuscript can be formally accepted you will need to complete some formatting changes, which you will receive in a follow up email. A member of our team will be in touch with a set of requests.

Please note that your manuscript will not be scheduled for publication until you have made the required changes, so a swift response is appreciated.

IMPORTANT: The editorial review process is now complete. PLOS will only permit corrections to spelling, formatting or significant scientific errors from this point onwards. Requests for major changes, or any which affect the scientific understanding of your work, will cause delays to the publication date of your manuscript.

Should you, your institution's press office or the journal office choose to press release your paper, you will automatically be opted out of early publication. We ask that you notify us now if you or your institution is planning to press release the article. All press must be co-ordinated with PLOS.

Thank you again for supporting Open Access publishing; we are looking forward to publishing your work in PLOS Computational Biology.

Best regards,

Daifeng Wang, Ph.D.

Academic Editor

PLOS Computational Biology

Can Yang

Section Editor

PLOS Computational Biology

***********************************************************

Reviewer's Responses to Questions

**Comments to the Authors:**

**Please note here if the review is uploaded as an attachment.**

Reviewer #1: All of my concerns have been properly addressed.

Reviewer #3: All my concerns have been sufficiently addressed.

**********

**Have the authors made all data and (if applicable) computational code underlying the findings in their manuscript fully available?**

The PLOS Data policy requires authors to make all data and code underlying the findings described in their manuscript fully available without restriction, with rare exception (please refer to the Data Availability Statement in the manuscript PDF file). The data and code should be provided as part of the manuscript or its supporting information, or deposited to a public repository. For example, in addition to summary statistics, the data points behind means, medians and variance measures should be available. If there are restrictions on publicly sharing data or code —e.g. participant privacy or use of data from a third party—those must be specified.

Reviewer #1: Yes

Reviewer #3: Yes

**********

PLOS authors have the option to publish the peer review history of their article (what does this mean?). If published, this will include your full peer review and any attached files.

If you choose “no”, your identity will remain anonymous but your review may still be made public.

**Do you want your identity to be public for this peer review?**       For information about this choice, including consent withdrawal, please see our Privacy Policy.

Reviewer #1: No

Reviewer #3: No

# Editor report: Acceptance letter

**DOI:** 10.1371/journal.pcbi.1014422.r007

**Authors:** Daifeng Wang, Ferhat Ay, Daifeng Wang, Ferhat Ay, Daifeng Wang, Can Yang

PCOMPBIOL-D-25-00949R2

Deciphering cell type-specific causal genetic effects on brain imaging-derived phenotypes and disorders with single-cell Mendelian randomization

Dear Dr Yang,

I am pleased to inform you that your manuscript has been formally accepted for publication in PLOS Computational Biology. Your manuscript is now with our production department and you will be notified of the publication date in due course.

The corresponding author will soon be receiving a typeset proof for review, to ensure errors have not been introduced during production. Please review the PDF proof of your manuscript carefully, as this is the last chance to correct any errors. Please note that major changes, or those which affect the scientific understanding of the work, will likely cause delays to the publication date of your manuscript.

Soon after your final files are uploaded, unless you have opted out, the early version of your manuscript will be published online. The date of the early version will be your article's publication date. The final article will be published to the same URL, and all versions of the paper will be accessible to readers.

For Research, Software, and Methods articles, you will receive an invoice from PLOS for your publication fee after your manuscript has reached the completed accept phase. If you receive an email requesting payment before acceptance or for any other service, this may be a phishing scheme. Learn how to identify phishing emails and protect your accounts at https://explore.plos.org/phishing.

Thank you again for supporting PLOS Computational Biology and open-access publishing. We are looking forward to publishing your work!

With kind regards,

Anita Estes

PLOS Computational Biology | Carlyle House, Carlyle Road, Cambridge CB4 3DN | United Kingdom ploscompbiol@plos.org | Phone +44 (0) 1223-442824 | ploscompbiol.org | @PLOSCompBiol
